# Prevalence, Awareness, and Treatment of Hypertension in 37 African Countries: Trends From 2003 to 2022

**DOI:** 10.1016/j.jacc.2025.09.1600

**Published:** 2025-12-09

**Authors:** Aboubakari Nambiema, Kouamivi Mawuenyegan Agboyibor, Jean-Marie Dangou, Marie Antignac, Cheick Bady Diallo, Joseph Chukwudi Okeibunor, Xavier Jouven, Farshad Farzadfar, Jean-Philippe Empana, African STEPS Group, Leanne Riley, Lubna Bhatti, Stafan Savin, Cheick Bady Diallo, Djamila Nadir-Azirou, Dismand Stéphane Houinato, Heluf G. Medhin, Bame Shatera, Isaïe Medah, Emília Castro Monteiro, René Charles Sylva, Alfred K. Njamnshi, Max Roge Koula, Justin Ndoyo, Mathias Roger Djidina, Mohamed Msaidie, Gisèle Kimbally-Kaky, Kouamelan Doua, Benjamin Longo-Mbenza, Goitom Mebrahtu Medhani, Lindiwe Tsabedze, Xolisile Dlamini, Fikru Tesfaye, Abebe Bekele Belayneh, Comlan Pearl Nsie Obame, Omar Badjie, Willian Kofi Bosu, Naby Moussa Baldé, Kibachio Joseph Muiruri Mwangi, John Nkonyana, C. Sanford Wesseh, Luke L. Bawo, Henri Fidele Marie Raharivohitra, Kelias Msyamboza, Nazoum Diarra, Albertino António Damasceno, Moussa Habibou, Adamou Amadou, Nyandwi Alypio, Marie Aimee Muhimpundu, Adolph Karenzi, Rosete Nahimana, Jean Baptiste Koama, Jean de Dieu Ngirabega, Andre Rusanganwa, Marie Fidele Mukazayire, Adolph Karenzi, Rosete Nahimana, Francois Uwinkindi, Gil Santana, Elizabeth Barros, Idalio Luis, Pascal Bovet, Mohamed Samai, Faiza Kassim, Mary Mayige, Kokou Agoudavi, Mofou Belo, Gerald Mutungi, Seter Siziya, Wilbroad Mutale

**Affiliations:** a Université Paris Cité, Inserm, PARCC, Paris, France; b CHU SO Lomé, Unité de Recherche en Santé des Populations (URESAP), Epidémiologie des maladies chroniques non transmissibles (EpiMaCnT), Lomé, Togo; c Université Paris Cité, Institut Santé Globale de Paris (ISGP), Paris, France; d World Health Organization Regional Office for Africa, Brazzaville, Congo; e Department of Pharmacy, Saint Antoine hospital, HUEP (Hôpitaux Universitaires Est Parisien), AP-HP (Assistance Publique-Hôpitaux de Paris), Paris, France; f World Health Organization Headquarters, Geneva, Switzerland; g African Research Network (ARN), Dakar, Senegal

**Keywords:** awareness, control, hypertension, pooled cross-sectional studies, treatment, WHO African Region

## Abstract

**Background:**

Despite the high burden of hypertension in Africa, multicountry and representative studies addressing hypertension-related outcomes are scarce.

**Objectives:**

We estimated the distribution, time trends, and determinants of hypertension status, awareness, treatment, and control in the World Health Organization (WHO) African Region (AFRO) countries.

**Methods:**

We analyzed individual-level data from 61 national, subnational, or community surveys, including WHO STEPwise approach to NCD risk factor Surveillance surveys and Demographic and Health Surveys, conducted between 2003 and 2022 in 37 AFRO countries. Prevalence rates and their 95% CIs were age-standardized and accounted for survey and study-level weights. ORs were estimated using hierarchical logistic regression analysis with random effects for country and survey year.

**Results:**

Of the 251,761 participants, 59.5% were female, mean age: 37.1 ± 12.3 in females and 38.6 ± 12.6 in males. Hypertension was diagnosed in 28.9% (95% CI: 27.6%-30.2%) of females and 26.1% (95% CI: 24.5%-27.7%) of males. Among hypertensive individuals, awareness was 62.4% (95% CI: 58.5%-66.3%) for females and 50.5% (95% CI: 44.5%-56.4%) for males; antihypertensive treatment was 15.0% (95% CI: 13.1%-16.9%) in females and 8.4% (95% CI: 6.7%-10.2%) in males; and control rates were 7.3% (95% CI: 5.8%-8.8%) for females and 3.7% (95% CI: 2.4%-4.9%) for males. In multivariable hierarchical analysis, prevalence of hypertension and awareness were associated with older age, higher body mass index, retirement, former smoking (females only), higher education level (with awareness only), and higher alcohol consumption (with awareness in males only). Conversely, lower odds of treatment and control were associated with current smoking and high alcohol consumption. Hypertension-related outcomes were also influenced by contextual (country-level) factors including life expectancy at birth, Gender Development Index, grams of fat per day per capita, Gross Domestic Product per capita, employment in agriculture, and urbanization. Between 2003 and 2022, the overall number of hypertension cases nearly doubled from 241 to 390 million, while treatment and control rates rose from 11.8% to 18.4% and from 7.6% to 9.2%, respectively.

**Conclusions:**

Despite improvement in hypertension awareness, the burden of untreated and uncontrolled hypertension in adults remains substantial in the AFRO with notable sex disparities.

## Graphical abstract

**Figure:**
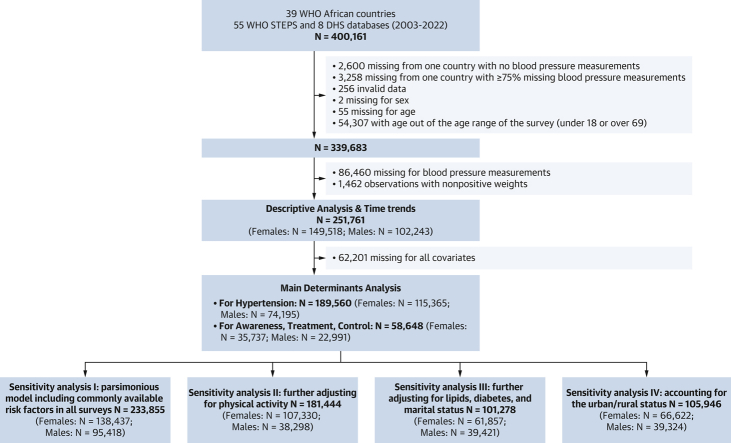

Hypertension, or high blood pressure, is 1 of the major contributors to the onset of cardiovascular disease, chronic kidney disease, and premature death worldwide.^1^

According to the World Health Organization (WHO) hypertension has been estimated to affect 1.3 billion adults worldwide in 2019, with 78% of them living in low- and middle-income countries.^2^ This is particularly true in Africa, where an epidemiological transition from infectious to noncommunicable diseases (NCD) is being operated, with the WHO African Region (AFRO) showing the highest prevalence of hypertension (27%) among low- and middle-income countries.^3^ Moreover, in 2021, hypertension has been responsible for approximately 10 million avoidable deaths worldwide, mainly due to stroke, ischemic heart disease, and kidney disease. More than 90% of these deaths were in low- and middle-income countries, with approximately 3 million deaths in the African continent.^4^ Accordingly, the reduction of hypertension prevalence and its effective control is 1 of the WHO’s central objective in the WHO Global Action Plan for the Prevention and Control of NCDs 2013-2030.^5^

Despite growing concerns, multi-country studies addressing simultaneously the 4 interconnected hypertension-related outcomes including the prevalence of hypertension, awareness, treatment, and control across multiple African nations and in representative samples remain scarce. In fact, only 3 studies, including NCD-RisC (NCD Risk Factor Collaboration),^6^ SevenCEWA (Seven Communities in East and West Africa)^7^ and H3Africa (Human Heredity and Health in Africa) AWI-Gen (Africa Wits-INDEPTH Partnership for the Genomic Studies),^8^ have examined the distribution of these 4 hypertension-related outcomes. Similarly, only 1 study, the SevenCEWA,^7^ has assessed the determinants of these 4 outcomes, while the remaining studies usually focused on 1 single hypertension-related outcome. Furthermore, although the NCD-RisC^6^ and the May Measurement Month Initiative (MMM)^9^^,^^10^ integrated data from >25 African countries, other pooled studies have focused on fewer than 10 countries.^7^^,^^8^ Understanding time trends in hypertension-related outcomes is crucial for evaluating intervention effectiveness and adapt future interventions. The NCD-RisC^6^ has examined the time trend of hypertension prevalence worldwide including Africa, but no studies have further analyzed the time trends of hypertension awareness, treatment, and control.

To the best of our knowledge, no prior multicountry study has assessed simultaneously the prevalence, associated factors, and 20-year time trends of hypertension, awareness, treatment, and control in Sub-Saharan Africa (SSA). To fill these gaps in knowledge, we pooled individual data from 61 national and subnational community-based surveys conducted between 2003 and 2022, and representing 37 countries of the AFRO. This represents the most comprehensive picture of hypertension related outcomes in the AFRO and a foundation to evaluate ongoing preventive programs but also inform on additional actions to be taken.

## Methods

### Study design and data sources

The AFRO comprises 47 member states, mostly SSA countries. We were granted access to the WHO's STEPwise approach to NCD risk factor Surveillance (STEPS) surveys (see later in the text) and Demographic and Health Surveys (DHS) conducted in 39 countries between 2003 and 2022. These 2 data sources have common study design, study protocol, and data collection. After excluding countries without blood pressure measurements, the individual data from 37 countries were analyzed. These data sets were sourced from 61 national, subnational (ie, covering 1 or more subnational regions), or community (1 or a small number [typically 1-5, depending on data availability] of communities), conducted under the WHO's STEPS or the DHS Program. These include 53 STEPS surveys and 8 DHS data sets. A list of data sources and their characteristics is provided in Supplemental Table 1. Reasons for noninclusion of some STEPS surveys, as well as the list of AFRO member countries, are presented in Supplemental Table 2.

STEPS is a population-based household survey of NCD risk factors among adults aged 18 to 69 years. The survey instrument is freely provided by WHO but is implemented voluntarily by countries, taking into account local and regional specificities (resources available), without compromising the comparability of data across the surveys. All WHO STEPS surveys^11^ follow a consistent sampling procedure and use standard questionnaires and protocols for data collection. The DHS are nationally representative household surveys that collect data on population, health, and nutrition. They usually include women aged 15 to 49 years and men aged 15 to 59 years, though age ranges may vary by country and survey module. Although each country implements the survey according to its needs and resources, all DHS follow standard methods, questionnaires, and sampling procedures to ensure comparability of data across countries and over time. These population-based surveys are accessible online on demand via official data repositories,^12^^,^^13^ for independent reanalysis and do not contain personal identifiers.

The current study applies only to the WHO AFRO, not to the entire African continent.

### Ethics statement

Each STEPS survey and DHS obtained ethical clearance from relevant national or institutional review boards in the respective countries. Informed consent was obtained from all participants before data collection. Administrative authorizations for the STEPS surveys and DHS data were obtained from the WHO Regional Office for Africa (institutional clearance number: AFR/ERC/2023/5.18) and the DHS Program, respectively.

### Hypertension-related outcomes

Standard measurement procedures were used by all investigators, who took 3 sitting blood pressure recordings at 3-minute intervals in the left arm ideally. For each participant, we calculated the average of the last 2 measurements of systolic blood pressure (SBP) or diastolic blood pressure (DBP) and excluded observations for which at least 1 of these measurements was missing.

*Hypertension* was defined as having SBP ≥140 mm Hg or DBP ≥90 mm Hg (or both) or taking medication for hypertension (treatment). We also reported the stage of hypertension according to the Hypertension Practice Guidelines of the International Society of Hypertension:^14^ normal (<130/85 mm Hg), high-normal (130-139/85-89 mm Hg), stage 1 hypertension (140-159/90-99 mm Hg), and stage 2 hypertension (≥160/100 mm Hg).

Undiagnosed hypertension was defined as participants who had a measured SBP ≥140 mm Hg and/or DBP ≥90 mm Hg during the survey but did not report any previous diagnosis of hypertension by a health professional.

*Awareness* was defined based on the responses to the following question: “Have you ever been told by a doctor or other health worker that you have raised blood pressure or hypertension?” or “In the past 2 weeks, have you taken any drugs (medication) for raised blood pressure prescribed by a doctor or other health worker?”

*Treatment* in STEPS surveys was defined based on the responses to the following 2 questions: 1) “In the past 2 weeks, have you taken any drugs (medication) for raised blood pressure prescribed by a doctor or other health worker?” 2) “During the past 2 weeks, have you been treated for raised blood pressure with drugs (medication) prescribed by a doctor or other health worker?” In DHS, treatment was defined based on the response to the following question: “Are you taking medication to control your blood pressure?”

*Control* was defined as having a measured SBP <140 mm Hg and DBP <90 mm Hg among those treated.

### Covariates

**Figure 1:**
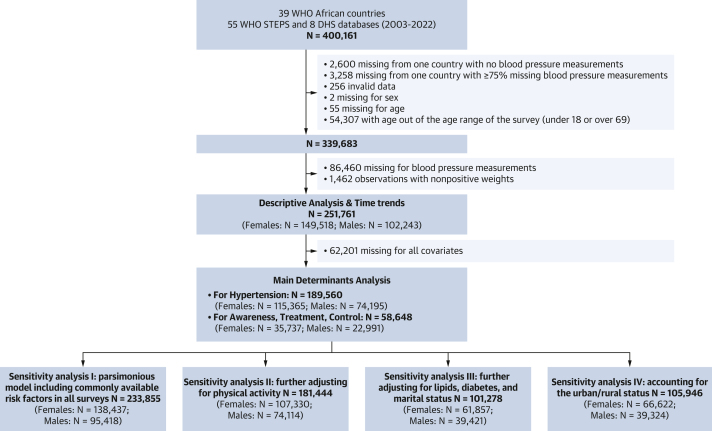
Study Flow Chart DHS = Demographic and Health Surveys; STEPS = World Health Organization's STEPwise approach to NCD risk factor Surveillance; WHO = World Health Organization.

The covariates at the individual level included age, body mass index (BMI), physical activity, smoking status, alcohol intake, highest level of education, work status, marital status, diabetes status, and measured high total cholesterol (mg/dL). Their definition is given in the Supplemental Text 1 and their level of availability in Figure 1 (study flow chart).

Contextual factors at the country level were considered in sensitivity analyses and included World Bank and United Nations Development Programme data. They consisted of unemployment rate, agricultural productivity, urbanization percentage, prevalence of undernourishment, gross domestic product per capita, life expectancy at birth, years of schooling, the Gender Development Index, grams of fat per day per capita, grams of protein per day per capita, and female participation in the workforce at the country level.

### Statistical analysis

Data were analyzed using SAS v9.4 TS Level 1M5 (SAS Institute) and RStudio 2024.09.0 Build 375 (Posit Software, PBC). All variables were recoded into identical categories and compiled into a combined dataset.

All analyses were systematically conducted by sex. Main analyses were conducted on the pooled data, whereas analyses by country are provided in the Supplemental Appendix.

The analyses of hypertension status (prevalence estimates, time-trends, and associated factors) was conducted in all participants, the analyses for hypertension awareness, treatment, and control were assessed only among those diagnosed with hypertension.

Continuous and categorical variables were described as mean (95% CI) and column percentages, respectively. The baseline characteristics were compared by sex and hypertension status using chi-square Rao-Scott test and Weighted Student’s, where appropriate.

### Prevalence of hypertension, awareness, treatment, and control

Prevalence estimates and their 95% CIs were age-standardized (direct method) using the WHO world population as the reference population.^15^ Prevalence estimates account for survey weights to reflect the survey sampling. To consider differences in sample size across the studies, an additional weight was assigned to each study and corresponded to the inverse of the study variance (or standard error).

### Trends in SBP, DBP, hypertension, awareness, treatment, and control

Trends from 2003 to 2022 in mean SBP and mean DBP, and in the prevalence of hypertension, awareness, treatment, and control were estimated using a spatiotemporal model.^16^^,^^17^ The detailed analysis is provided in Supplemental Text 2. Importantly, this analysis estimates yearly blood pressure values and prevalence estimates at the country and not at the individual level. Missing outcome data for a given year in a given country were handled through the spatiotemporal model, which used available data from the same country as well as data from neighboring countries, while accounting for clustering by country and survey year through random effects. Spatial correlation and temporal trends were modeled following the approach described by Bakka et al.^16^ Notably, the spatiotemporal analysis enabled us to extend the analysis to the 10 missing African countries, thereby providing trends for all 47 member states in the AFRO.

Furthermore, the yearly absolute number of adults aged 18 to 69 years with hypertension was computed by multiplying for each country the yearly weighted prevalence of hypertension by the available census for adults aged 18 to 69 years at that year according to the United Nations Population Division World Population Prospects (Supplemental Tables 3a and 3b).^18^

### Factors associated with hypertension prevalence, awareness, treatment, and control

Complete-case analyses were conducted to determine factors associated with the 4 hypertension-related outcomes. Separate logistic regression analysis with random effects for country and survey year (treated in discrete form) were used to estimate ORs and their 95% CIs. Models were adjusted for a priori defined key risk factors collected at the individual level including age, BMI, education level, smoking status, alcohol intake, and work status. Although being partially available in DHS surveys, BMI (women only in DHS) and alcohol intake (4 of 8 DHS) were retained due to their known importance as risk factors; in addition, these variables were available in the 2 countries without STEPS surveys (South Africa and Namibia).

We further expanded the analysis of the determinants of the 4 hypertension-related outcomes by incorporating contextual factors in the hierarchical mixed-effects logistic regression models. To minimize the risk of collinearity, only variables with a correlation coefficient below 0.7 were included in the regression models.^19^^,^^20^ For pairs of factors with a correlation coefficient of 0.7 or higher, the one with greater data coverage was retained.

### Sensitivity analyses

First, linear mixed models with random effects for country and survey year were applied to assess the factors associated with SBP and DBP.

Second, a more parsimonious model containing commonly available risk factors in DHS and STEPS (Figure 1) including age, education level, smoking status, and work status was conducted.

Third, the main models were further and sequentially adjusted for physical activity, which was available in 95% (n = 181,444) of the individuals, and then for diabetes, marital status, and high total cholesterol, which were available in 75% (n = 148,538), 78% (n = 142,219), and 63% (n = 119,324) of the population, respectively (Figure 1).

Fourth, we addressed the influence of rural-urban status in a subsample of 105,946 participants (55%) in whom the information was available. We described the distribution of the 4 hypertension-related outcomes by rural-urban status and repeated the analysis of their determinants further adjusting for the rural-urban status.

Last, we provided time trends of the 4 related hypertension outcomes and of the absolute number of hypertension cases using observed instead of imputed data. This information was available in at least 1 STEPS survey and/or DHS each year between 2003 and 2022 except 2018.

### Role of the funding source

There was no funding source for this study. Specific countries and the AFRO had full access to the data in the study.

## Results

### Study sample characteristics

After exclusions (Figure 1), the study population included 251,761 participants (59.5% females; mean age: 37.1 ± 12.3 years in females and 38.6 ± 12.6 years in males), representing 148 million adults in the AFRO from 37 countries.

Out of the 37 countries analyzed, 19 had data from 2 or more surveys representing 75% of the study sample. There were 12 countries (representing 32.4% of the total) from West Africa, 12 (32.4%) from Eastern Africa, 7 (18.9%) from Central Africa, 5 (13.5%) from Southern Africa, and 1 country (2.7%, Algeria) from North Africa. The country data characteristics are provided in Supplemental Table 1.

**Figure 2:**
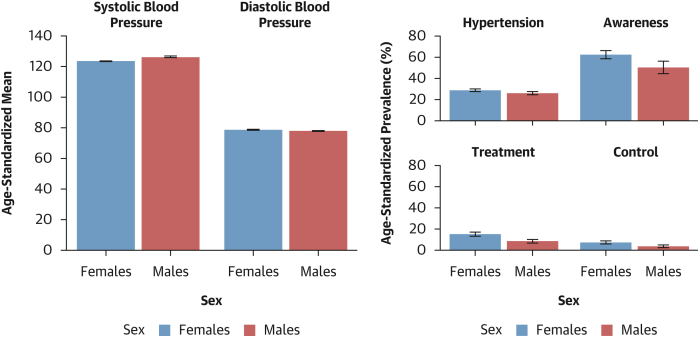
Sex-Stratified Age-Standardized Hypertension Prevalence, Awareness, Treatment, Control Rates, and Mean Blood Pressure Levels Abbreviations as in Figure 1.

The age-standardized mean SBP and DBP were 123.9 mm Hg (95% CI: 123.4-124.5 mm Hg) and 79.0 mm Hg (95% CI: 78.6-79.3 mm Hg) in females, and 126.5 mm Hg (95% CI: 125.8-125.8 mm Hg) and 78.2 mm Hg (95% CI: 77.7-78.7 mm Hg) in males, respectively (Figure 2).

The distribution of the characteristics by hypertension status in females and males are reported in Table 1. Females with hypertension were more frequently overweight or obese, physically inactive, unemployed, students, or unpaid workers, divorced, separated, or widowed, less likely to smoke or consume alcohol, and had lower education levels. By contrast, hypertensive males were more often underweight, employed, single, more physically active, more likely to smoke and consume alcohol, and had higher education levels. The distribution of contextual factors by sex according to hypertension status is provided in Supplemental Table 4.

**Table 1:** Characteristics of Participants by Sex According to Hypertension Status

	Overall Population	Females (n =149,518; 50.5%)			Males (n = 102,243; 49.5%)		
		Hypertension	No Hypertension	*P* Value^c^	Hypertension	No Hypertension	*P* Value^c^
Participants^a^	251,761 (100.0)	43,240 (25.9)	106,287 (74.1)		29,666 (24.3)	72,577 (75.7)	
Age, y	35.2 (35.2-35.3)	42.1 (42.0-42.2)	32.6 (32.5-32.8)	<0.0001	40.9 (40.8-41.1)	33.6 (33.5-33.7)	<0.0001
Age group, y				<0.0001			<0.0001
18-24	33,340 (22.0)	2,118 (10.1)	18,638 (25.3)		1,619 (12.8)	10,965 (25.9)	
25-34	80,223 (32.2)	8,573 (22.6)	41,312 (37.3)		6,045 (24.7)	24,293 (32.9)	
35-44	64,086 (21.8)	11,265 (23.4)	26,752 (21.2)		7,228 (22.4)	18,841 (21.6)	
45-54	42,376 (13.5)	10,827 (22.0)	12,895 (10.5)		7,166 (18.6)	11,488 (12)	
55-69	31,736 (10.4)	10,457 (21.9)	6,681 (5.7)		7,608 (21.5)	6,990 (7.5)	
Body mass index, kg/m^2^^b^				<0.0001			<0.0001
<18.5	20,817 (12.6)	2,559 (7.6)	9,553 (12.7)		2,026 (8.5)	6,679 (15.5)	
18.5-24.99	122,975 (63.4)	16,542 (47.3)	54,899 (61.9)		14,117 (62.7)	37,417 (70.7)	
25-29.99	45,323 (16.2)	10,968 (23.8)	21,109 (17.5)		5,760 (20.4)	7,486 (11.0)	
≥30	26,146 (7.8)	10,971 (21.3)	11,235 (7.9)		2,180 (8.4)	1,760 (2.8)	
Physical activity^b^				<0.0001			0.0016
None	29,589 (15.6)	6,953 (19.4)	13,225 (18.2)		3,455 (13.3)	5,956 (12.2)	
1 to 149 min/wk moderate or 1 to 74 min/wk vigorous or 1 to 149 min/wk combination	46,590 (21.4)	10,526 (28.3)	21,421 (26.0)		5,063 (17.9)	9,580 (15.7)	
≥150 min/wk moderate or ≥75 min/wk vigorous or ≥150 min/wk combination	119,380 (63.0)	19,163 (52.3)	45,304 (55.8)		16,162 (68.8)	38,751 (72.1)	
Smoking				<0.0001			<0.0001
Never or quit >12 mo	209,797 (85.5)	37,818 (93.3)	98,568 (96.0)		21,049 (74.2)	52,362 (75.8)	
Former, quit ≤12 mo	3,601 (1.8)	694 (1.5)	728 (0.7)		741 (3.9)	1,438 (2.3)	
Current	33,053 (12.8)	3,433 (5.2)	5,340 (3.4)		6,836 (22.0)	17,444 (21.9)	
Alcohol intake^b^				0.6841			0.0244
Never or quit >12 mo	122,102 (68.4)	25,161 (77.3)	54,838 (76.8)		12,394 (58.3)	29,709 (60.3)	
Current	49,834 (11.6)	7,980 (11.8)	20,768 (12.1)		5,735 (10.9)	15,351 (11.3)	
Heavy episodic drink	33,169 (19.9)	3,753 (10.8)	7,560 (11.1)		7,241 (30.8)	14,615 (28.4)	
Highest level of education				<0.0001			0.0014
No formal schooling/<primary school/primary school	162,423 (73.3)	30,308 (79.3)	71,098 (76.3)		17,125 (68.6)	43,892 (69.8)	
Secondary school	54,774 (16.1)	8,170 (12.0)	22,905 (14.6)		6,836 (17.5)	16,863 (18.6)	
High school	16,451 (3.6)	1,951 (2.8)	5,932 (3.1)		2,609 (4.5)	5,959 (4.1)	
University+	10,888 (7.0)	1,317 (5.9)	3,492 (6.0)		2,032 (9.5)	4,047 (7.5)	
Work status				<0.0001			<0.0001
Employed	45,950 (14.1)	6,572 (9.6)	16,449 (8.6)		7,566 (23.0)	15 363 (18.4)	
Self-employed	67,722 (33.5)	10,629 (29.2)	24,153 (28.4)		9,728 (40.2)	23,212 (38.1)	
Farmer/manual worker	26,582 (0.6)	2,128 (1.1)	9,343 (0.6)		2,912 (0.9)	12,199 (0.4)	
Unemployed	27,078 (9.9)	5,204 (11.8)	13,519 (11.0)		2,392 (8.1)	5,963 (8.8)	
Student/nonpaid	61,621 (40.0)	14,082 (46.4)	34,273 (50.9)		3,189 (22.1)	10,077 (32.7)	
Retired	3,647 (1.5)	776 (1.6)	381 (0.3)		1,586 (5.3)	904 (1.3)	
Others	3,481 (0.2)	622 (0.2)	1,586 (0.2)		325 (0.3)	948 (0.3)	
Marital status^b^				<0.0001			<0.0001
Single	39,811 (21.9)	4,492 (9.8)	15,838 (18.0)		4,009 (18.6)	15,472 (31.1)	
Married/cohabiting	132,246 (68.8)	20,854 (68.9)	57,449 (70.0)		16,014 (76.5)	37,929 (65.2)	
Divorced/separated/widowed	23,673 (9.2)	7,607 (21.3)	11,273 (12.0)		1,600 (4.8)	3,193 (3.7)	
Diabetes^b^				<0.0001			<0.0001
High total cholesterol, >190 mg/dL^b^	164,864 (95.5)	28,203 (89.8)	70,492 (97.3)		18,816 (91.7)	47,353 (97)	

Values are n (%) or mean (95% CI). Categorical variables were described as column percentages. Survey weights were incorporated into the estimates of all percentages.

DBP = diastolic blood pressure; SBP = systolic blood pressure.

a Variables were described as row percentages.

b For body mass index (BMI), alcohol use, physical activity, marital status, diabetes, and total cholesterol information were available in 85% (n = 215,261 individuals), 84% (n = 211,860), 78% (n = 195.559), 78% (n = 195,730), 74% (n = 187,168), and 50% (n = 125,212) of the population, respectively. In all DHS data, BMI was available among females only.

c *P* value of chi-square Rao-Scott test was used for categorical variables, and weighted *t*-test for continuous variables.

### Prevalence estimates of hypertension status, awareness, treatment, and control

**Figure 3:**
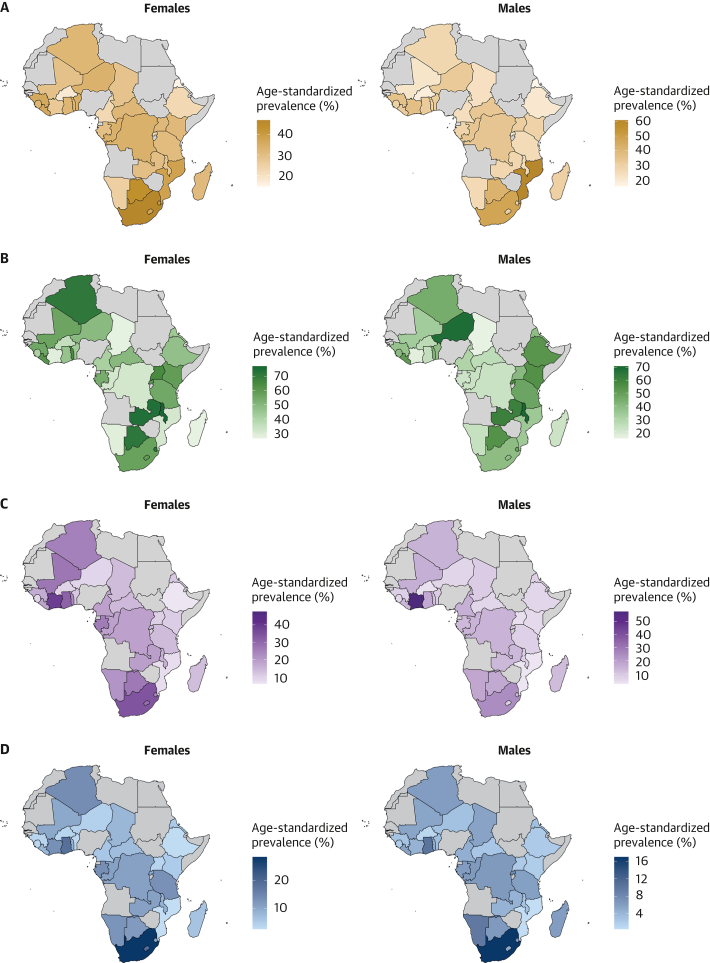
Sex-Stratified Maps of Age-Standardized Prevalence, Hypertension, Awareness, and Treatment Rates by Country (A) Sex-stratified maps of age-standardized hypertension prevalence rates by country. (B) Sex-stratified maps of age-standardized hypertension awareness rates by country. (C) Sex-stratified maps of age-standardized hypertension treatment rates by country. (D) Sex-stratified maps of age-standardized hypertension control rates by country. Abbreviations as in Figure 1.

**Figure 4:**
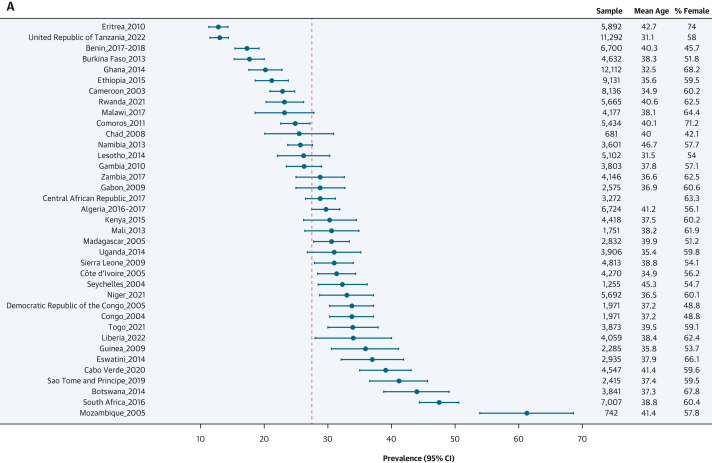

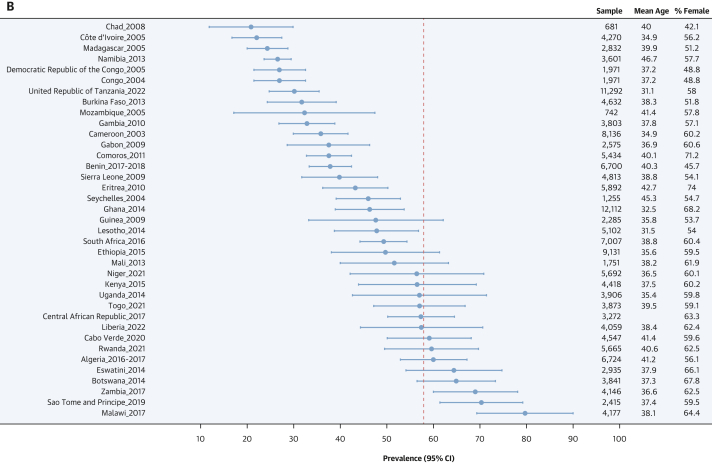

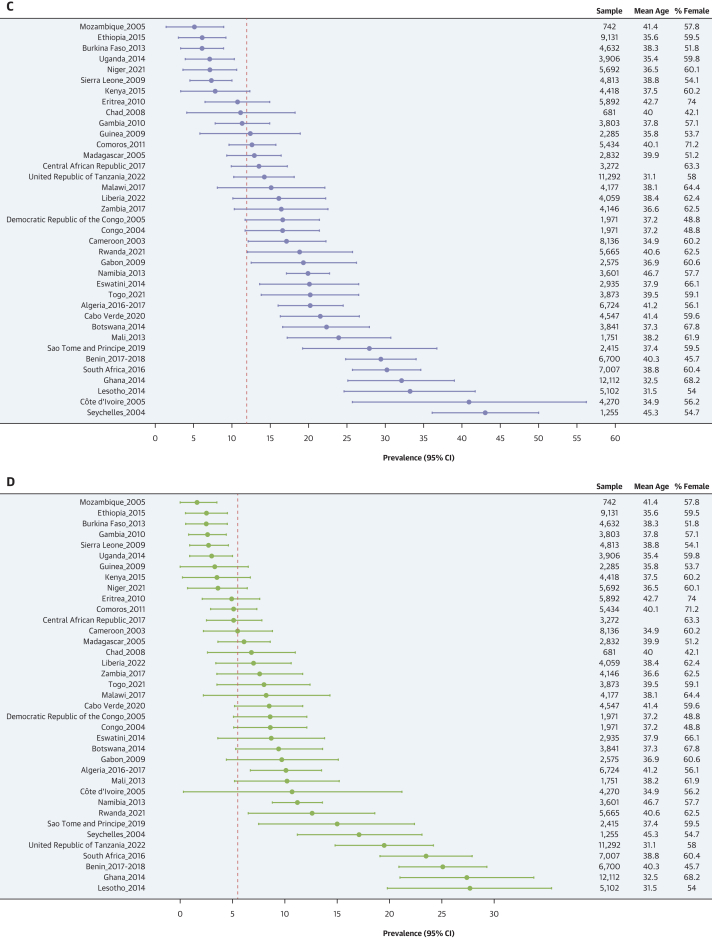
Country-Level Latest Estimates of Hypertension Prevalence, Awareness, Treatment, and Control Rates (A) Country-level latest estimates of hypertension prevalence rates. The plot represents the prevalence of hypertension for a given country across the World Health Organization (WHO) African region. Each dot represents the most recent estimate of hypertension prevalence rate for a given country, with horizontal lines indicating the 95% CI. The dotted vertical line represents the pooled estimate of hypertension prevalence rate and is included to aid visual interpretation. (B) Country-level latest estimates of hypertension awareness rates. The plot represents the prevalence of hypertension awareness among people with hypertension for a given country across the WHO African region. Each dot represents the most recent estimate of hypertension awareness rate for a given country, with horizontal lines indicating the 95% CI. The dotted vertical line represents the pooled estimate of hypertension awareness rate and is included to aid visual interpretation. (C) Country-level latest estimates of hypertension treatment rates. The plot represents the prevalence of hypertension treatment among people with hypertension for a given country across the WHO Africa region. Each dot represents the most recent estimate of hypertension treatment rates for a given country, with horizontal lines indicating the 95% CI. The dotted vertical line represents the pooled estimate of hypertension treatment rate and is included to aid visual interpretation. (D) Country-level latest estimates of hypertension control rates. The plot represents the prevalence of hypertension control among people with hypertension for a given country across the WHO Africa region. Each dot represents the most recent estimate of hypertension control rate for a given country, with horizontal lines indicating the 95% CI. The dotted vertical line represents the pooled estimate of hypertension control rate and is included to aid visual interpretation.

**Figure 5:**
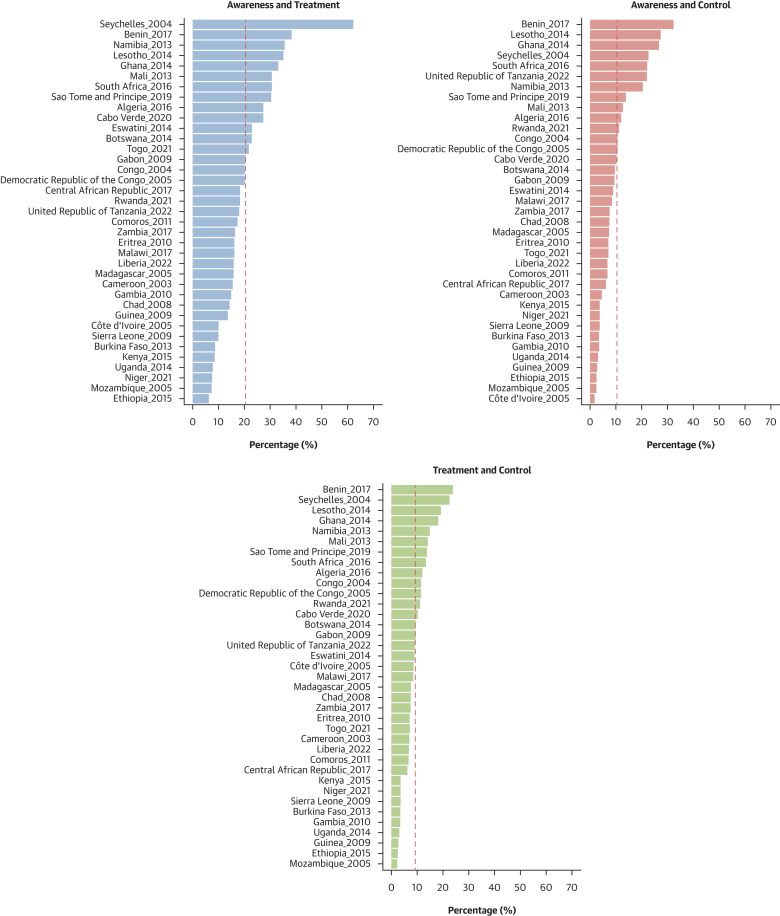
Joint Weighted Proportions of Hypertension Awareness, Treatment, and Control in Each Country This figure presents the latest available survey data for each country (males and females combined). The bars show the joint weighted proportions of “Awareness & Treatment,” “Awareness & Control,” and “Treatment & Control,” respectively. The red dashed line represents the average value of each joint proportion across all the countries.

As shown in Figure 2, females had higher age-standardized prevalence of hypertension than males: 28.9% (95% CI: 27.6%-30.2%) vs 26.1% (95% CI: 24.5%-27.7%) (*P* for sex difference = 0.001). The prevalence of undiagnosed hypertension was higher in females than in males: 25.0% (95% CI: 23.9%-26.0%) vs 22.4% (95% CI: 21.1%-23.7%) (*P* for sex difference = 0.003). Similarly, among participants with hypertension, awareness (62.4% [95% CI: 58.5%-66.3%] vs 50.5% [95% CI: 44.5%-56.4%]; *P* for sex difference < 0.001), treatment rates (15.0% [95% CI: 13.1%-16.9%] vs 8.4% [95% CI: 6.7%-10.2%]; *P* for sex difference < 0.001), and control rates (7.3% [95% CI: 5.8%-8.8%] vs 3.7% [95% CI: 2.4%-4.9%]; *P* for sex difference < 0.001) were systematically higher in females compared with males. These sex differences were seen for the 4 related outcomes across age (Supplemental Figure 1), education level (Supplemental Figure 2), country income level (Supplemental Figure 3), and rural/urban status (Supplemental Figure 4). The distribution of blood pressure categories by sex is illustrated in Supplemental Figure 5. Figure 3 presents maps of the prevalence of hypertension, awareness, treatment, and control rates by country and by sex. Figure 4 shows the forest plots of the latest prevalence estimates of the 4 related hypertension outcomes by country combining data for men and women. They are highlighting marked geographic variation across the African region. The joint weighted proportions of hypertension awareness, treatment, and control in each country show that countries that perform relatively well in 1 domain also tend to perform relatively well in the others (Figure 5). Nevertheless, the averaged joint weighted proportions of hypertension awareness and treatment, hypertension awareness and control, and hypertension treatment and control remained critically low, respectively 20%, 10%, and 10%.

### Time trends in hypertension prevalence, awareness, treatment, and control, and in blood pressure levels

**Figure 6:**
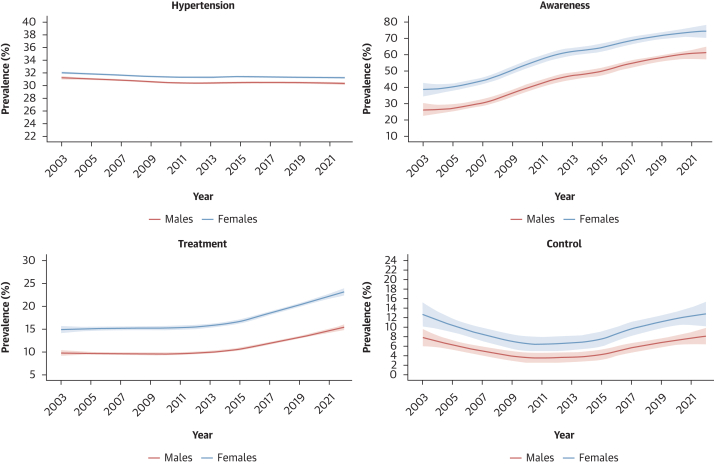
Sex-Stratified Trend in Age-Standardized Prevalence of Hypertension-Related Outcomes From 2003 to 2022 Trends are based on imputed data for all the 47 countries in the World Health Organization (WHO) African region.

**Figure 7:**
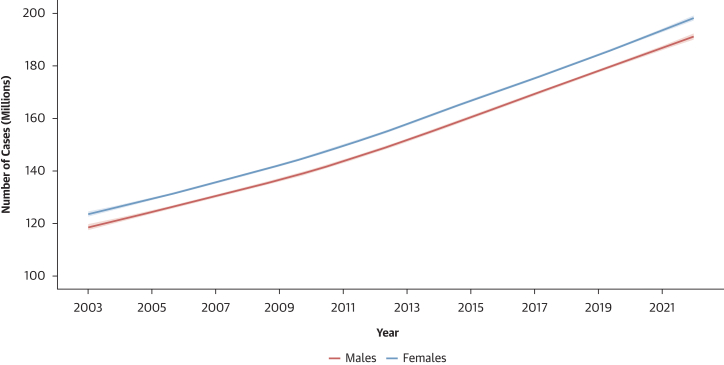
Sex-Stratified Trend in the Absolute Number of Hypertensive Individuals From 2003 to 2022 Trends are based on imputed data for all the 47 countries in the World Health Organization (WHO) African region.

The prevalence of hypertension remained stable, from 32.0% in 2003 to 31.4% in 2022 in females (*P* for trend = 0.597) and from 31.0% in 2003 to 30.2% in 2022 in males (*P* for trend = 0.525) (Figure 6). However, during the same period, the absolute number of individuals with hypertension almost doubled from 123 million (95% CI: 115-132 million) to 199 million (95% CI: 185-213 million) females and from 118 million (95% CI: 110-126 million) to 191 million (95% CI: 178-204 million) males (Figure 7). Hypertension awareness rates nearly doubled in both sexes, from 40.0% to 72.8% in females (*P* for trend < 0.001) and from 26.9% to 59.3% in males (*P* for trend < 0.001) (Figure 6). Treatment rates increased from 14.4% to 22.0% for females (*P* for trend < 0.001) and from 9.2% to 14.7% for males (*P* for trend < 0.001) (Figure 6). Control rates remained critically low, from 9.6% to 11.2% in females (*P* for trend < 0.001) and from 5.7% to 7.1% in males (*P* for trend = 0.004) (Figure 6).

During the same time interval, age-standardized SBP decreased from 128.6 to 124.4 mm Hg for females (*P* for trend < 0.001) and from 130.1 to 125.6 mm Hg for males (*P* for trend < 0.001), whereas DBP showed no clinically significant decrease from 80.0 to 79.5 mm Hg for females (*P* for trend = 0.001) and from 79.3 to 78.7 mm Hg for males (*P* for trend = 0.001), respectively (Supplemental Figures 6 and 7).

### Factors associated with the 4 hypertension-related outcomes

#### Factors associated with hypertension

**Figure 8:**
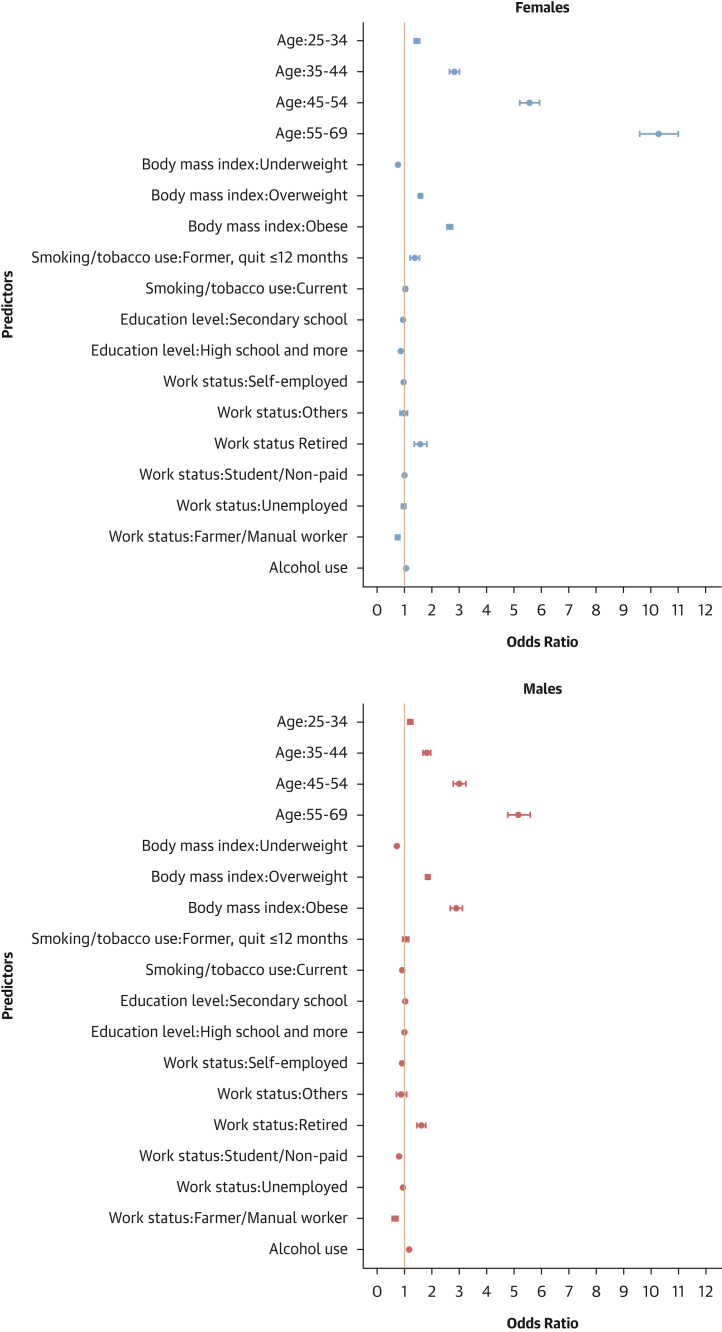
Determinants of Hypertension Status: Sex-Stratified Multivariable Hierarchical Analysis Logistic regression analyses with random effects for country and survey year were conducted to determine individual factors associated with hypertension. The analysis was conducted among all participants. Dots represent adjusted ORs with 95% CIs for each predictor. An OR >1 indicates a positive association (increased odds of hypertension), and an OR <1 indicates a negative association (reduced odds).

In both males and females, older age, being overweight or obese, and being retired were associated with higher odds of hypertension. Conversely, being underweight and farmer/manual worker was associated with lower odds of hypertension. In females, former smoking was related to higher odds of hypertension. In males, alcohol consumption was associated with higher odds of hypertension, whereas the opposite was found for a student/unpaid worker or for unemployed status (Figure 8).

#### Factors associated with hypertension awareness

**Figure 9:**
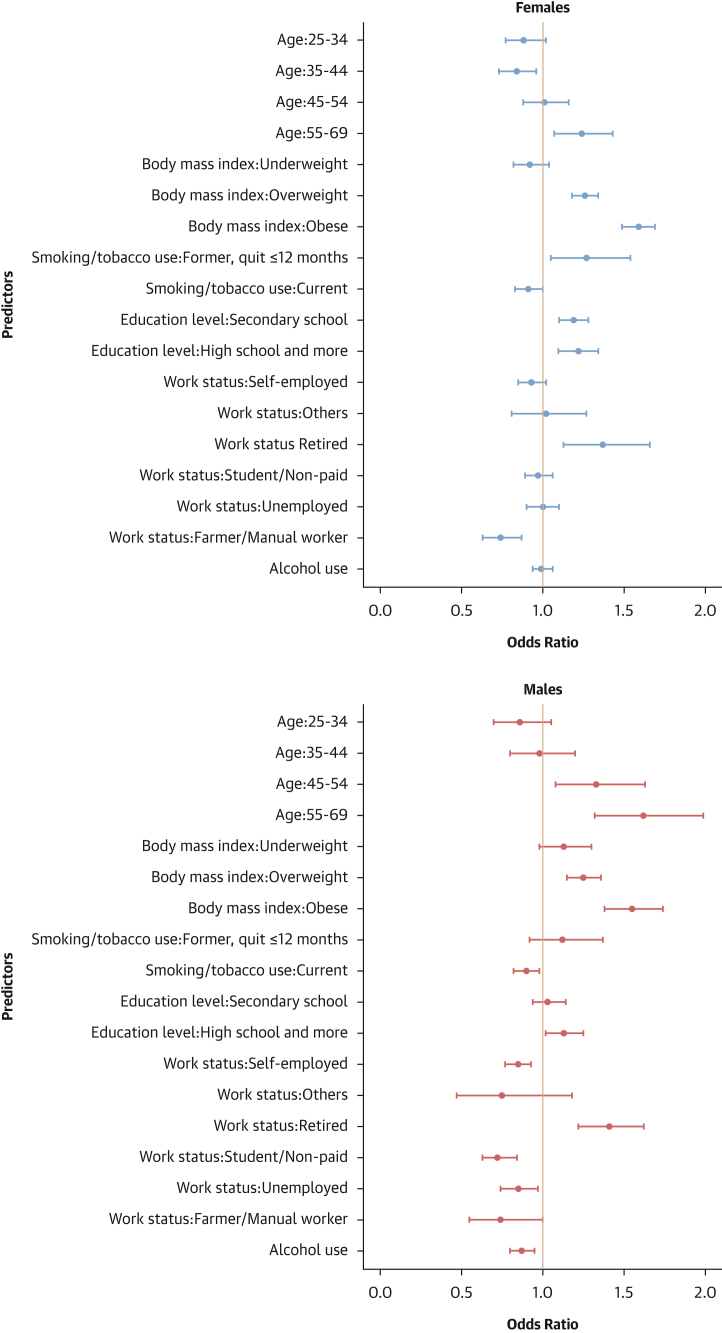
Determinants of Hypertension Awareness: Sex-Stratified Multivariable Hierarchical Analysis The analysis was conducted among hypertensive individuals only. Logistic regression analyses with random effects for country and survey year were used. Dots represent adjusted ORs with 95% CIs for each predictor. An OR >1 indicates a positive association (increased odds of hypertension awareness), and an OR <1 indicates a negative association (reduced odds).

In both sexes, older age, being overweight or obese, attaining high school education or higher, and being retired were associated with increased odds of hypertension awareness. In females, former smoking and achieving education beyond the primary level were linked to higher odds of hypertension awareness, whereas being a farmer or manual worker was associated with lower odds of awareness. In males, current smoking, self-employed, a student/unpaid worker, unemployed, or alcohol consumption were linked to reduced odds of hypertension awareness (Figure 9).

#### Factors associated with hypertension treatment

**Figure 10:**
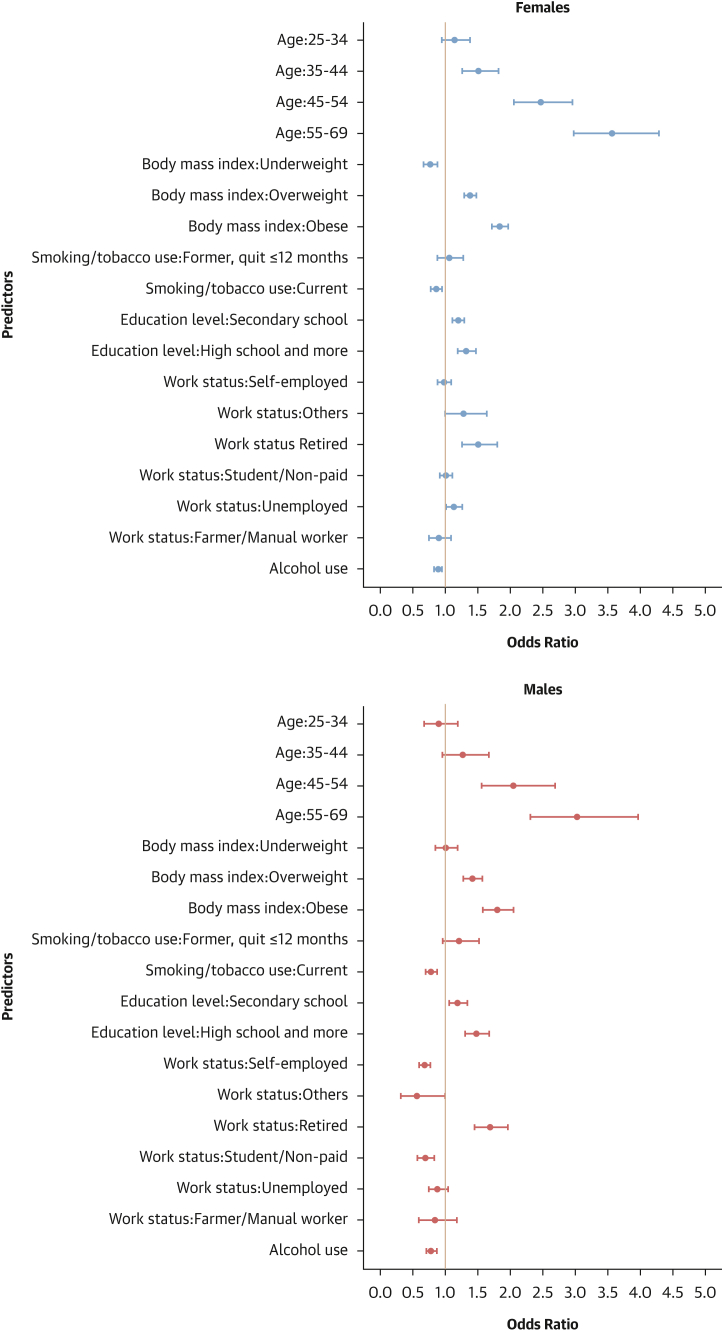
Determinants of Hypertension Treatment: Sex-Stratified Multivariable Hierarchical Analysis The analysis was conducted among hypertensive individuals only. Logistic regression analyses with random effects for country and survey year were used. Dots represent adjusted ORs with 95% CIs for each predictor. An OR >1 indicates a positive association (increased odds of hypertension treatment), and an OR <1 indicates a negative association (reduced odds).

Older age, being overweight or obese, attaining education beyond the primary level, and being retired were associated with higher odds of hypertension treatment in both sexes. Conversely, current smoking and alcohol consumption were linked to lower odds of hypertension treatment. In females, being underweight was associated with lower odds of treatment. In males, being self-employed and a student/unpaid worker were linked to reduced odds of treatment (Figure 10).

#### Factors associated with hypertension control

**Figure 11:**
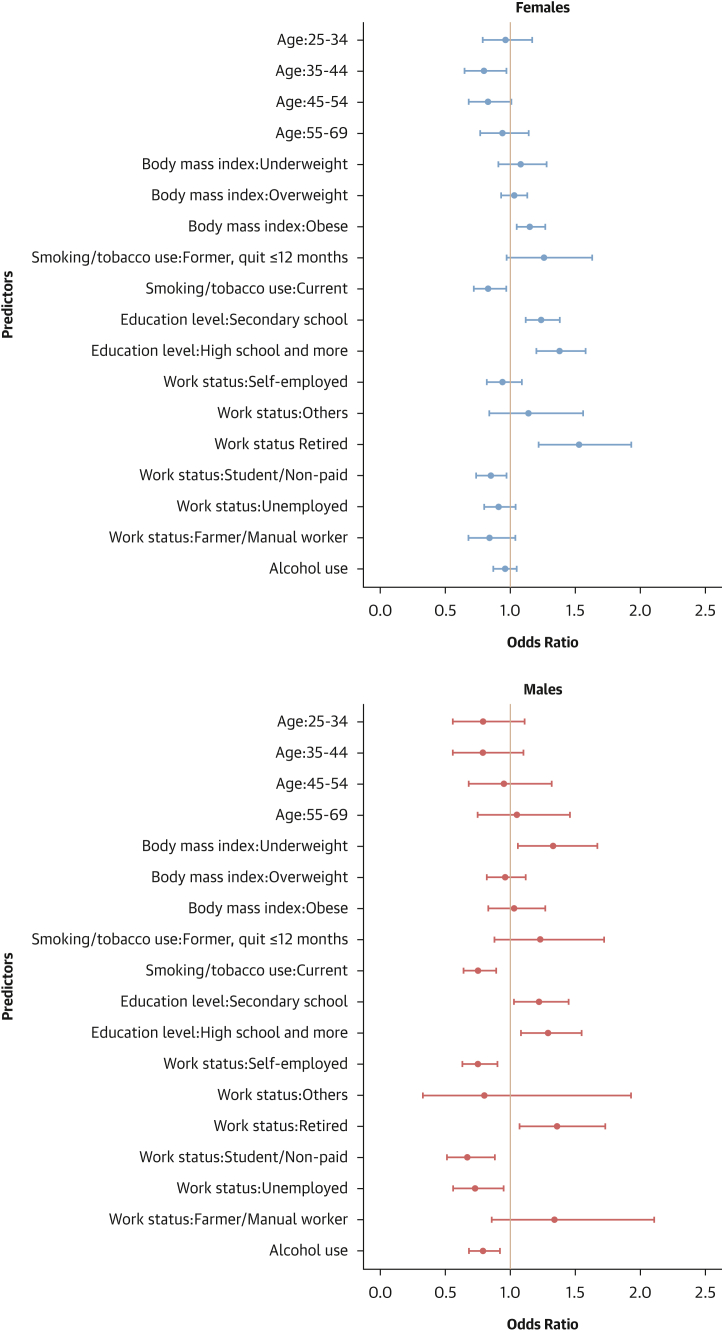
Determinants of Hypertension Control: Sex-Stratified Multivariable Hierarchical Analysis The analysis was conducted among hypertensive individuals only. Logistic regression analyses with random effects for country and survey year were used. Dots represent adjusted ORs with 95% CIs for each predictor. An OR >1 indicates a positive association (increased odds of hypertension control), and an OR <1 indicates a negative association (reduced odds).

Finally, attaining education beyond the primary level and being retired were associated with higher odds of hypertension control in both sexes. Conversely, current smoking and being a student or nonpaid worker were linked to lower odds of hypertension control. In females, obesity was linked to higher odds of hypertension control. In males, being underweight was associated with higher odds of hypertension control, whereas being self-employed, a student/unpaid worker, unemployed, and alcohol consumption were linked to reduced odds of hypertension control (Figure 11).

Random effects for survey years were statistically significant in all models (*P* < 0.0001).

#### Contextual factors associated with hypertension-related outcomes

**Figure 12:**
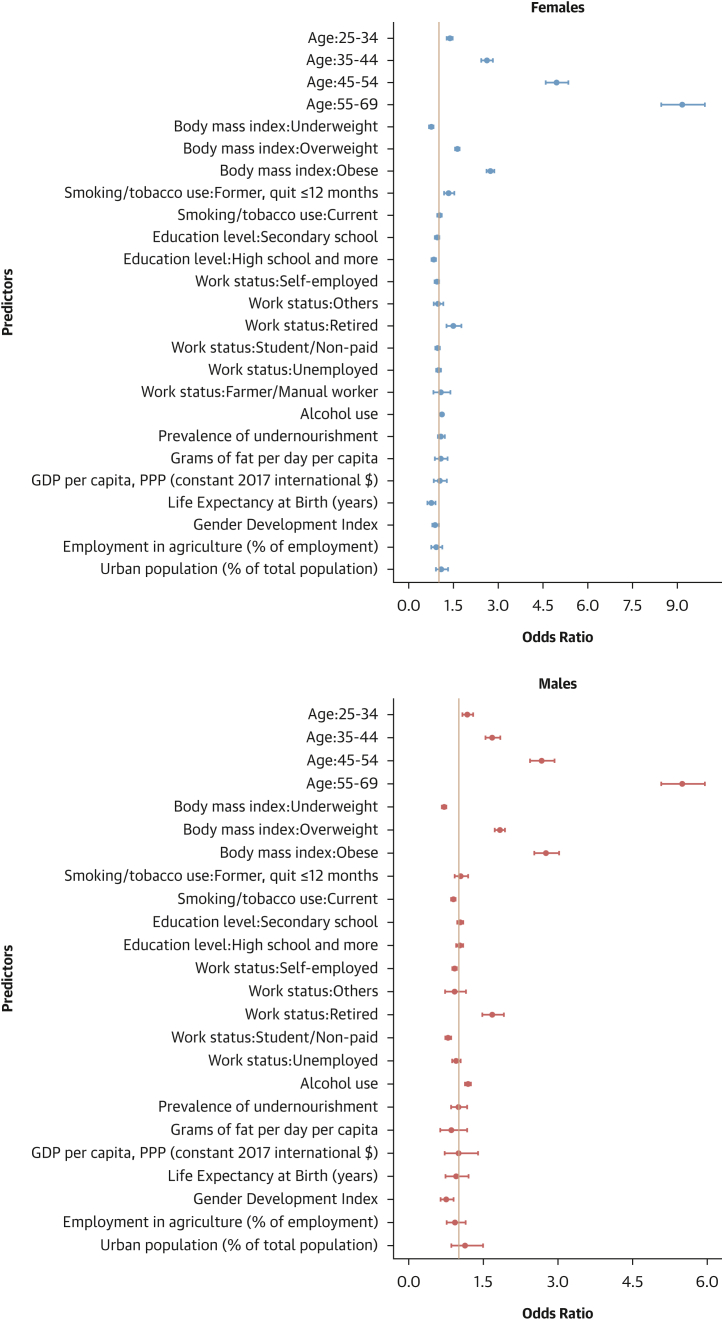
Determinants of Hypertension Status: Sex-Stratified Multivariable Hierarchical Analysis, Adjusted for Contextual Factors ORs and their 95% CIs were estimated via logistic regression analyses with random effects for country and survey year. The analysis was conducted among all participants. Dots represent adjusted ORs and horizontal lines their 95% CIs for each predictor. An OR >1 indicates a positive association (increased odds of hypertension), and an OR <1 indicates a negative association (reduced odds).

**Figure 13:**
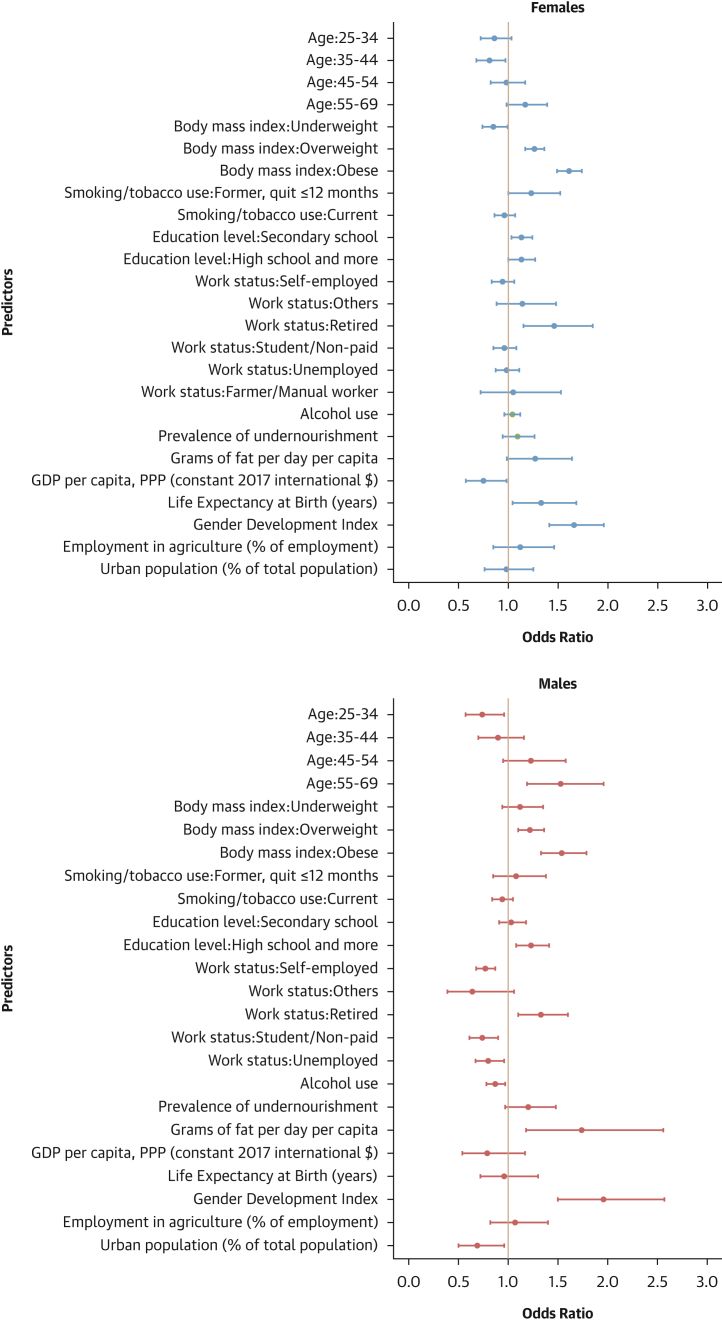
Determinants of Hypertension Awareness: Sex-Stratified Multivariable Hierarchical Analysis, Adjusted for Contextual Factors ORs and their 95% CIs were estimated via logistic regression analyses with random effects for country and survey year. The analysis was conducted among hypertensive individuals only. Dots represent adjusted ORs and horizontal lines their 95% CIs for each predictor. An OR >1 indicates a positive association (increased odds of hypertension awareness), and an OR <1 indicates a negative association (reduced odds).

**Figure 14:**
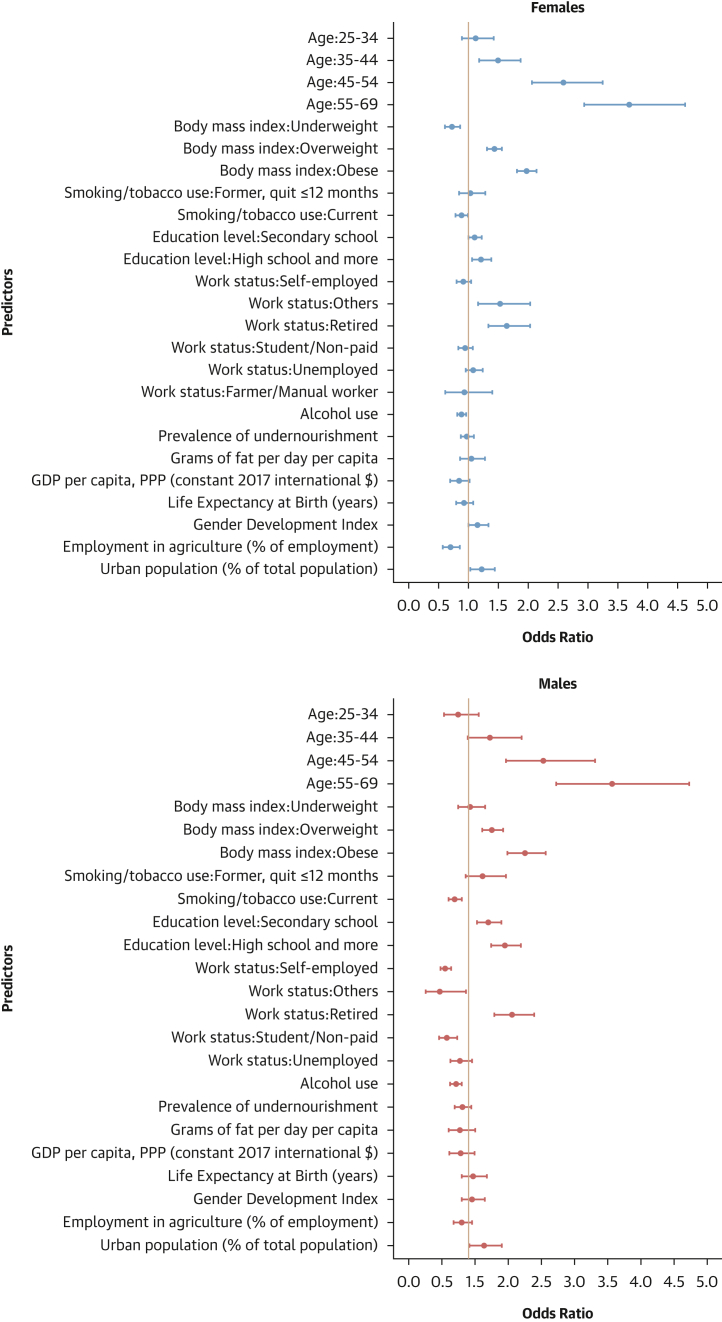
Determinants of Hypertension Treatment: Sex-Stratified Multivariable Hierarchical Analysis, Adjusted for Contextual Factors ORs and their 95% CIs were estimated via logistic regression analyses with random effects for country and survey year. The analysis was conducted among hypertensive individuals only. Dots represent adjusted ORs and horizontal lines their 95% CIs for each predictor. An OR >1 indicates a positive association (increased odds of hypertension treatment), and an OR <1 indicates a negative association (reduced odds).

**Figure 15:**
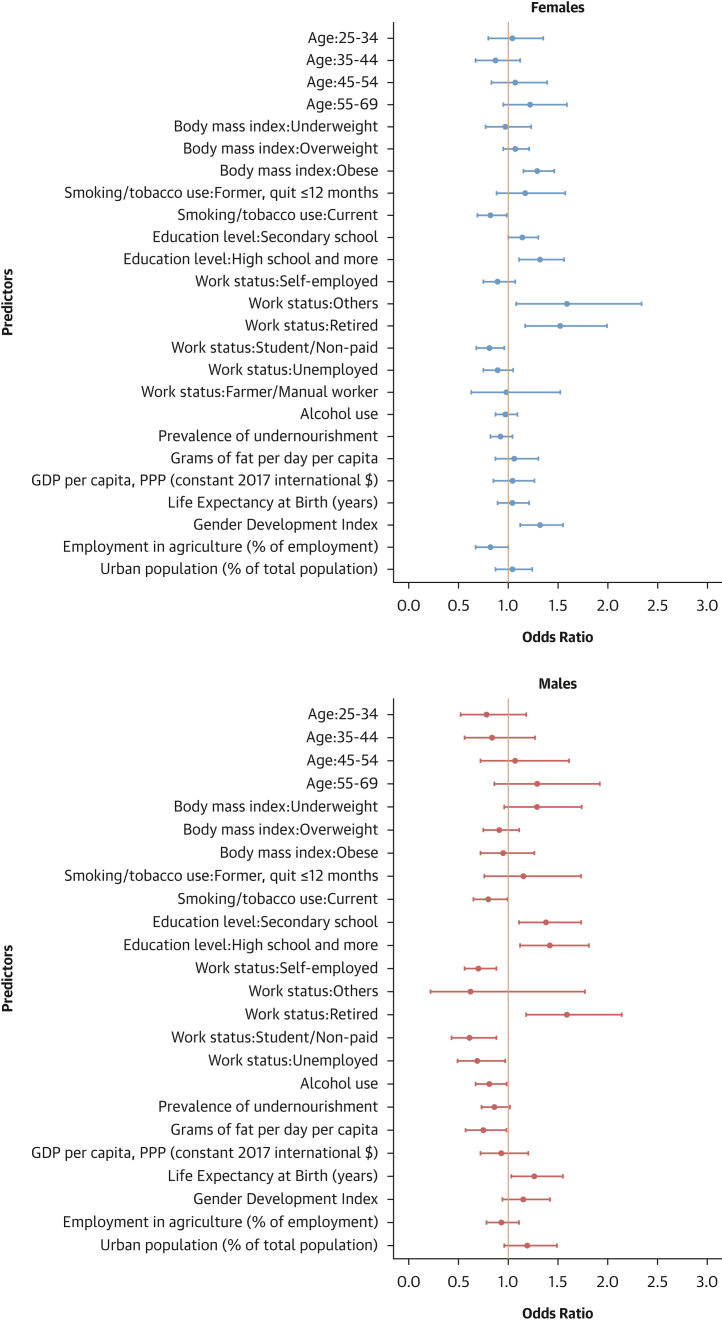
Determinants Of Hypertension Control: Sex-Stratified Multivariable Hierarchical Analysis, Adjusted for Contextual Factors ORs and their 95% CIs were estimated via logistic regression analyses with random effects for country and survey year. The analysis was conducted among hypertensive individuals only. Dots represent adjusted ORs and horizontal lines their 95% confidence intervals for each predictor. An OR >1 indicates a positive association (increased odds of hypertension control), and an OR <1 indicates a negative association (reduced odds).

As shown in Figures 12, 13, 14, and 15, adding contextual factors to the models did not change the association of individual risk factors with hypertension-related outcomes. In these analyses, life expectancy at birth was associated with lower odds of hypertension prevalence in females, whereas the Gender Development Index was associated with lower odds of hypertension prevalence in males (Figure 12). The Gender Development Index was associated with increased odds of hypertension awareness in both sexes. In females, life expectancy at birth was associated with increased odds of hypertension awareness, whereas GDP per capita was associated with lower odds of hypertension awareness. In males, grams of fat per day per capita was associated with increased odds of hypertension awareness while urbanization percentage was related to lower odds of hypertension awareness (Figure 13). The urbanization percentage was associated with higher odds of hypertension treatment in both sexes whereas employment in agriculture was associated with lower odds of hypertension treatment in females only (Figure 14). Gender Development Index was associated with higher odds of hypertension control in females, whereas life expectancy at birth was associated with higher odds of hypertension control in males (Figure 15).

### Sensitivity analyses

The factors associated with SBP and DBP in multivariable linear regression analyses (Supplemental Figures 8 and 9) were consistent with those related to hypertension status in both sexes.

The factors associated with the 4 hypertension-related outcomes in both sexes remained unchanged when using a more parsimonious model (Supplemental Figures 10 to 13), when further adjusting for physical activity (Supplemental Figures 14 to 17) or after further adjustment for diabetes, marital status, and high total cholesterol (Supplemental Figures 18 to 21).

Analysis by urban status indicates that in both sexes, the determinants of the 4 hypertension-related outcomes did not change after further adjusting for the rural-urban status (Supplemental Figures 22 to 25).

The time-trends analysis based on observed data is consistent with the findings derived from the spatiotemporal model (Supplemental Figures 26 to 36).

## Discussion

This analysis of the burden of hypertension in 250,000 African adults from 37 countries of the AFRO region revealed 3 main findings. First, hypertension affects 1 in 3 adult females and 1 in 4 adult males. Among people diagnosed with hypertension, more than one-half were aware of their condition, but only 15% of females and 8% of males were treated, and only 7% of females and 4% of males had controlled hypertension. Second, hypertension-related outcomes were influenced by both individual and contextual (country-level) risk factors. Third, although the 20-year trend analysis from 2003 to 2022 suggests a slight decrease in hypertension prevalence, the absolute number of hypertension diagnoses nearly doubled over the same period, from 123 to 199 million in females and 118 to 191 million in males.

With 61 national and subnational surveys in 37 countries, this is by far the largest and most comprehensive study addressing the prevalence, associated factors, and time trends of hypertension, awareness, treatment, and control in the African region. Compared with the MMM study and the NCD-RisC study, the largest multicountry nationwide studies in Africa with 25 countries so far, the age-standardized prevalence of hypertension (29% in females and 26% in males) in the present study is similar to the 26% age-standardized prevalence reported in the MMM study in both sexes,^9^ but lower than the latest (ie, 2019) analysis of the NCD-RisC study, reporting prevalence rates of 48% in females and 34% in males in SSA.^6^ Of note, however, study participants were older in NCD-RisC compared with the STEPS surveys and DHS (30-79 years compared with 18-69 years or 15-59 years). Strikingly, the doubling of the absolute number of individuals affected by hypertension from 2003 to 2022 to reach 199 million females and 191 million males in 2022 contrasts with the steady trend in the hypertension prevalence over the same period, a finding consistent with the trends reported in the NCD-RisC from 1999 to 2019.^2^^,^^6^ This rise is likely driven by factors such as urbanization and related behavioral changes (ie, dietary changes and physical inactivity), increased screening and diagnosis.^21^^,^^22^ This also likely due to the coexistence of an ageing population, the habitual provider of hypertension cases, and an important growth of the young population, an additional provider of hypertension cases in Africa.^6^^,^^23^ Altogether, this doubling of hypertension cases is compensated by the growing population explaining the apparent stable prevalence of hypertension rates over time.

The age-standardized hypertension awareness rates in the present study (62% in females and 51% in males) were consistent with the SevenCEWA study,^7^ but were higher than those from the MMM study.^10^ However, awareness was solely based on the use of antihypertensive medications in the MMM study, whereas knowledge of having raised blood pressure was additionally considered in the current study. The almost doubling prevalence rates of awareness between 2003 and 2022—from 40% to 73% in females and 27% to 59% in males—in the present study is likely reflecting expanded screening by initiatives like MMM that promote the importance of regular blood pressure monitoring,^10^ the growing implementation of community-based programs,^24^, ^25^, ^26^ broader efforts to integrate hypertension management into primary health care systems,^27^ and integration of WHO Package of Essential NCD (PEN) into primary care across the region.^28^

The high level of hypertension awareness and its significant increased trend contrast with the dramatically low level of both treatment and control of hypertension globally and over time. The reported age-standardized hypertension treatment rates (15% in hypertensive females and 8% in hypertensive males) and the even lower rates of age-standardized hypertension control (7% in hypertensive females and 4% in hypertensive males) are considerably lower than those reported in the NCD-RisC,^6^ MMM,^10^ SevenCEWA,^7^ and the H3Africa AWI-Gen,^8^ where treatment rates ranged from 18% to 50% and control rates from 12% to 22%. The MMM study relies on voluntary participation, attracting individuals who are likely already more aware of their health status. The other 2 studies included older populations than the participants in the present study, the current study clearly showing that older age is a strong risk factor for hypertension treatment and control. We may have underestimated treatment rates, given that treatment was evaluated using a 2-week time window. In the African context with limited treatment affordability, such time window is probably too short and restrictive to reliably identify untreated patients. Furthermore, the control rates might differ according to adherence to treatment, but such information was unavailable in STEPS surveys and DHS. A recent systematic review indicates a level of adherence to antihypertensive treatment of 34% in Africa,^29^ a finding corroborating the results of a clinical study in 12 SSA countries reporting that two-thirds of hypertensive patients followed in specialized clinics had poor-to-medium adherence to antihypertensive treatment.^30^ Interestingly, studies conducted in hypertensive patients attending hypertension clinics in SSA have reported uncontrolled rates of hypertension of 77.4%.^31^ Altogether, these low rates of hypertension treatment and control echo the systemic barriers such as limited access to health care services, affordability, and the availability of quality medications,^32^, ^33^, ^34^, ^35^ along with the use of traditional treatments.^36^ Specifically, the SEVEN study^37^ showed that up to 16% of cardiovascular drugs in SSA, particularly calcium channel blockers, are of poor quality, with rates reaching 50% in informal street markets. Furthermore, the EIGHT (Evaluation of Hypertension in Sub-Saharan Africa) study^34^ highlighted that many patients with severe hypertension are still treated with monotherapy, possibly reflecting gaps in guideline adherence, clinical inertia, and socioeconomic constraints, and control remains suboptimal even with combination therapy. While challenging, comprehensive strategies that include enhancing primary health care capacity, strengthening pharmaceutical regulatory systems to combat counterfeit and substandard medicines, promoting generic drugs, implementing financial support mechanisms, such as subsidies or insurance schemes, and promoting community-based interventions to support long-term management of hypertension are urgently needed.

We noted striking sex differences in hypertension awareness, treatment, and control rates in favor of females compared with males. Studies have shown that females more than males regularly interact with health care professionals to access birth control, child health, and for gynecological health, which may increase opportunities for blood pressure diagnosis and hypertension awareness.^38^, ^39^, ^40^ Gender-related factors may also operate through differences in health-seeking behaviors,^40^ gender norms,^41^ exposure to lifestyle risk factors, and differing perceptions of disease risk and personal vulnerability,^42^ thereby influencing detection, treatment, and control of hypertension. However, more studies are needed to explain sex and gender difference in the burden of hypertension in Africa.

This is the first African multicountries study connecting contextual factors with the burden of hypertension. These contextual factors are completing the role played by lifestyle and socioeconomic risk factors at the individual level shown here and in the SevenCEWA.^7^ Higher rates of awareness, treatment, and control were found in urban regions-countries, countries with higher life expectancy at birth and Gender Development Index values, and according to fat consumption per capita. This is likely reflecting more developed health care infrastructure, greater equity in access to health services, and increased health literacy, which tend to be more favorable in urbanized and socioeconomically advantaged settings.^43^, ^44^, ^45^ Urban areas often have a denser distribution of health facilities and trained personnel, facilitating regular screening and follow-up, whereas higher Gender Development Index scores may indicate reduced gender-based disparities in health-seeking behavior, improving detection and management for both sexes. Similarly, longer life expectancy often indicates more developed health systems and stronger infrastructure for managing chronic diseases. Altogether, these results suggest that tackling hypertension in the AFRO requires multicomponent and multilevel interventions targeting both the individuals but also their contexts of living.

The findings of the present study have implications. The doubling in the number of hypertension cases over 20 years coupled with the extremely low treatment and control rates are of major concerns and threaten the capacities of already fragile health care systems in the region. Recent initiatives such as the ACHIEVE (African Control of Hypertension through Innovative Epidemiology and a Vibrant Ecosystem) initiative^46^ led by 34 African experts and supported by organizations such as the WHO, the World Hypertension League, and Resolve to Save Lives, have been proposed, but not yet implemented, to enhance the prevention, treatment, and control of hypertension in Africa through comprehensive strategies, including public education, evidence-based guidelines, and the WHO's HEARTS program for improving cardiovascular health and reducing the burden of heart disease at the primary health care level. In this context, and in the long term, implementing primordial prevention strategies, aiming at preventing the development of risk factors in the first place, may be a complimentary, but crucial, preventative strategy.^47^, ^48^, ^49^ Accordingly, higher cardiovascular health scores have been associated with lower risk of hypertension onset in other populations^49^, ^50^, ^51^, ^52^ but such data are lacking in the African context. Finally, the consistent observed associations of hypertension outcomes with markers of social and health system development highlight actionable levers for policy such as investing in urbanization and equitable health access, promoting gender equity, and strengthening public health education to improve hypertension outcomes across diverse African contexts.

### Study limitations

Despite the standardized data collection procedures and measures of STEPS surveys and DHS, the national and subnational representativeness of the data, and the multicountry nature of the study, it relies on 2 sources of data. Blood pressure was measured 3 times at 1 single occasion during physical examination, which may overestimate hypertension prevalence compared with 24-hour ambulatory blood pressure monitoring.^53^ Awareness and treatment definitions relied on a 2week time window, which may be inappropriate in the African context due to the lack of treatment affordability. Residual confounding factors cannot be excluded because factors such as treatment adherence or health care access were not measured. The survey targets adults aged 18 to 69 years, so that the study findings cannot be extrapolated to younger or older groups. Additionally, the trends analysis relies on modeled estimates for missing countries and missing years, and may have introduced uncertainties that do not completely capture the real-world variability. However, the consistency between the imputed and observed data support the robustness of the approach. Finally, the current study findings only applied to the WHO AFRO and not to the entire African continent.

## Conclusions

The analysis of 61 STEPS surveys and DHS in 37 countries highlights that the burden of hypertension remains substantial, the rates of treatment and control are alarmingly low, and stresses that the doubling of hypertension cases over the last 2 decades represents a major threat for the already fragile health infrastructures in the WHO AFRO.

## Funding Support and Author Disclosures

Dr Nambiema is supported by a postdoctoral grant from the Lefoulon-Delalande Foundation. All other authors have reported that they have no relationships relevant to the contents of this paper to disclose.
