# Supplementary material for: Prevalence, Awareness, and Treatment of Hypertension in 37 African Countries: Trends From 2003 to 2022
Source: J Am Coll Cardiol. 2025 Dec 9;86(23):2263–88. doi: 10.1016/j.jacc.2025.09.1600 (PMC12675140; doi:10.1016/j.jacc.2025.09.1600)
Supplement: Supplementary Material [file mmc1.pdf]

Prevalence, awareness and treatment of hypertension in 37 African countries: Trends from 2003 to 2022

Aboubakari Nambiema, PhD[1,2,3*], Kouamivi Mawuenyegan Agboyibor, MD[1,4], Jean-Marie Dangou, MD[4], Marie Antignac, PharmD, PhD [1,3,5], Cheick Bady Diallo, MD[4], Joseph Chukwudi Okeibunor, PhD[6], Xavier Jouven, MD, PhD [1,3,7], Farshad Farzadfar, MD, PhD [6], Jean-Philippe Empana, MD, PhD[1,3]

Supplemental Material

Table of contents

Supplemental Text 1. Covariate definitions 5
Supplemental Text 2. Data management and statistical methods 6
Supplemental Table 1. Data sources used in the analysis 7
Supplemental Table 2. Rationale for Non-Inclusion of STEPS Surveys 9
Supplemental Table 3a. Distribution of United Nations Population for the World Health Organization region by year and sex 10
Supplemental Table 3b. Distribution of United Nations Population for the World Health Organization region by year, country, and sex 11
Supplemental Table 4. Distribution of contextual factors by sex according to hypertension status 31
Supplemental Figure 1. Sex-stratified hypertension prevalence, awareness, treatment, and control rates by age group. 32
Supplemental Figure 2. Sex-stratified age-standardized hypertension prevalence, awareness, treatment, and control rates by education level. 32
Supplemental Figure 3. Sex-stratified age-standardized hypertension prevalence, awareness, treatment, and control rates by country income level. 33
Supplemental Figure 4. Sex-stratified age-standardized hypertension prevalence, awareness, treatment, and control rates by rural/urban status. 33
Supplemental Figure 5. Sex-stratified age-standardized prevalence of blood pressure categories. 34
Supplemental Figure 6. Sex-stratified trend in age-standardized mean systolic blood pressure from 2003–2022. 35
Supplemental Figure 7. Sex-stratified trend in age-standardized mean diastolic blood pressure from 2003–2022. 35
Supplemental Figure 8. Determinants of systolic blood pressure levels: sex-stratified multivariable hierarchical analysis. 36
Supplemental Figure 9. Determinants of diastolic blood pressure levels: sex-stratified multivariable hierarchical analysis. 37
Supplemental Figure 10. Determinants of hypertension status: sex-stratified multivariable hierarchical analysis, adjusted for covariates commonly available in STEPS and DHS surveys (parsimonious model). 38
Supplemental Figure 11. Determinants of hypertension awareness: sex-stratified multivariable hierarchical analysis, adjusted for covariates commonly available in STEPS and DHS surveys (parsimonious model). 39
Supplemental Figure 12. Determinants of hypertension treatment: sex-stratified multivariable hierarchical analysis, adjusted for covariates commonly available in STEPS and DHS surveys (parsimonious model). 40
Supplemental Figure 13. Determinants of hypertension control: sex-stratified multivariable hierarchical analysis, adjusted for covariates commonly available in STEPS and DHS surveys (parsimonious model). 41
Supplemental Figure 14. Determinants of hypertension status: sex-stratified multivariable hierarchical analysis, adjusted for physical activity. 42

Supplemental Figure 15. Determinants of hypertension awareness: sex-stratified multivariable hierarchical analysis, adjusted for physical activity. 43
Supplemental Figure 16. Determinants of hypertension treatment: sex-stratified multivariable hierarchical analysis, adjusted for physical activity. 44
Supplemental Figure 17. Determinants of hypertension control: sex-stratified multivariable hierarchical analysis, adjusted for physical activity. 45
Supplemental Figure 18. Determinants of hypertension status: sex-stratified multivariable hierarchical analysis, adjusted for diabetes, marital status, and cholesterol levels. 46
Supplemental Figure 19. Determinants of hypertension awareness: sex-stratified multivariable hierarchical analysis, adjusted for diabetes, marital status, and cholesterol levels. 47
Supplemental Figure 20. Determinants of hypertension treatment: sex-stratified multivariable hierarchical analysis, adjusted for diabetes, marital status, and cholesterol levels. 48
Supplemental Figure 21. Determinants of hypertension control: sex-stratified multivariable hierarchical analysis, adjusted for diabetes, marital status, and cholesterol levels. 49
Supplemental Figure 22. Determinants of hypertension status: sex-stratified multivariable hierarchical analysis, adjusted for rural/urban status. 50
Supplemental Figure 23. Determinants of hypertension awareness: sex-stratified multivariable hierarchical analysis, adjusted for rural/urban status. 51
Supplemental Figure 24. Determinants of hypertension treatment: sex-stratified multivariable hierarchical analysis, adjusted for rural/urban status. 52
Supplemental Figure 25. Determinants of hypertension control: sex-stratified multivariable hierarchical analysis, adjusted for rural/urban status. 53
Supplemental Figure 26. Sex-stratified trend in age-standardized prevalence of hypertension from 2003–2022 using observed (non-imputed) data.................................... 54
Supplemental Figure 27. Sex-stratified trend in the number of hypertensive individuals from 2003–2022 using observed (non-imputed) data. 54
Supplemental Figure 28. Sex-stratified trend in age-standardized prevalence of awareness from 2003–2022 using observed (non-imputed) data. 55
Supplemental Figure 29. Sex-stratified trend in age-standardized prevalence of treatment from 2003–2022 using observed (non-imputed) data. 55
Supplemental Figure 30. Sex-stratified trend in age-standardized prevalence of control from 2003–2022 using observed (non-imputed) data. 56
Supplemental Figure 31. Observed and imputed averaged systolic blood pressure values from 2003 to 2022 in the WHO African region by sex and age group (18–24, 25–34, 35–44, 45–54, and 55–69 years) for each available survey for each year. Green dots represent observed values and blue dots represent imputed values. Data were imputed using the spatio-temporal model.[3] 57
Supplemental Figure 32. Observed and imputed averaged diastolic blood pressure from 2003 to 2022 in the WHO African region by sex and age group (18–24, 25–34, 35–44, 45–54, and 55–69 years) for each available survey for each year. Green dots represent observed values and blue dots represent imputed values. Data were imputed using the spatio-temporal model.[3] 57

Supplemental Figure 33. Observed and imputed prevalence of hypertension from 2003 to 2022 in the WHO African region by sex and age group (18–24, 25–34, 35–44, 45–54, and 55–69 years) for each available survey for each year. Green dots represent observed values and blue dots represent imputed values. Data were imputed using the spatio-temporal model.[3] ... 58

Supplemental Figure 34. Observed and imputed prevalence of hypertension awareness values from 2003 to 2022 in the WHO African region by sex and age group (18–24, 25–34, 35–44, 45–54, and 55–69 years) for each available survey for each year. Green dots represent observed values and blue dots represent imputed values. Data were imputed using the spatio-temporal model.[3] ... 58

Supplemental Figure 35. Observed and imputed prevalence of hypertension treatment values from 2003 to 2022 in the WHO African region by sex and age group (18–24, 25–34, 35–44, 45–54, and 55–69 years) for each available survey for each year. Green dots represent observed values and blue dots represent imputed values. Data were imputed using the spatio-temporal model.[3] ... 59

Supplemental Figure 36. Observed and imputed prevalence of hypertension control values from 2003 to 2022 in the WHO African region by sex and age group (18–24, 25–34, 35–44, 45–54, and 55–69 years) for each available survey for each year. Green dots represent observed values and blue dots represent imputed values. Data were imputed using the spatio-temporal model.[3] ... 59

References ... 60

Supplemental Text 1. Covariate definitions

Body Mass Index (BMI) (in all STEPS surveys; only in women in the DHS surveys) is a widely used measure to classify adults into categories of underweight (BMI < 18.5 kg/m^2), normal weight (BMI 18.5–24.9 kg/m^2), overweight (BMI 25–29.9 kg/m^2), or obesity (BMI ≥ 30 kg/m^2) according to the World Health Organization (WHO). It is calculated by dividing a person's weight in kilograms by the square of their height in meters (kg/m^2).

Physical Activity (in STEPS only) is defined as engaging in moderate- or vigorous-intensity exercise each week, with the following three levels of activity: (1) **Low activity:** 0 minutes of activity, or 1–149 minutes per week of moderate-intensity activity, or 1–74 minutes per week of vigorous-intensity activity, or a combination of 1–149 minutes per week of both, (2) **Moderate activity**: 150 minutes or more per week of moderate-intensity activity, or 75 minutes or more per week of vigorous-intensity activity, or a combination of ≥150 minutes of both moderate and vigorous activity, and (3) **High activity**: Engaging in more than the minimum recommendations (e.g., ≥300 minutes per week of moderate-intensity or ≥150 minutes per week of vigorous-intensity activity).

Smoking/tobacco use is categorized into three levels: (1) **Current smoker**: An individual who actively smokes any tobacco products, such as cigarettes, cigars or pipes, (2) **Former, quit ≤12 months**: An individual who smoked in the past but stopped smoking no more than 12 months ago, (3) **Never or quit >12 months**: An individual who has never smoked or smoked in the past but stopped smoking for at least 12 months.

Alcohol intake is categorized into three levels: (1) **Never or quit >12 months**: A person who never drank alcohol or who drank in the past but stopped drinking for at least 12 months, (2) **Current**: An individual who actively drinks alcohol, and (3) **Heavy episodic drink**: Someone who has had at least 6 standard alcoholic drinks on at least one occasion in the past 30 days.

Highest level of education is categorized into four levels: (1) **No formal schooling/<primary school/primary school**: typically refers to primary education or less, (2) **Secondary school**: refers to secondary school education, (3) **High school**: refers to high school education, (4) **University+**: refers to tertiary education, such as university or college degrees.

Work status is defined in seven categorized as follows: (1) Employed, (2) Self-employed, (3) Farmer/Manual worker, (4) Unemployed, (5) Student/Non-paid, (6) Retired, and (7) Others.

Marital status is defined in three categorized as follows: (1) **Single**: individuals who have never married or are not currently in a partnership, (2) **Married/cohabiting**: individuals who are legally married or are currently in a partnership, and (3) **Divorced/Separated/Widowed**: individuals who were previously married but are no longer married, whose spouse has died.

Diabetes is defined as a diagnosis of diabetes, typically confirmed by fasting blood glucose tests or by taking medication to manage blood glucose levels.

High total cholesterol (mg/dL) is defined as having a total cholesterol level above **190 mg/dL**.

Country income level is defined by the World Bank income classifications: 1) Low income, (2) Lower middle-income, and (3) Upper middle-income. The ‘country income level’ variable was constructed by using information on the country's income level at the time of the survey.

Supplemental Text 2. Data management and statistical methods

We used data of WHO-STEPwise approach to Surveillance (STEPS) surveys and Demographic and Health Surveys (DHS) obtained from the WHO African Region (AFRO) and from publicly available multi-country and national measurement surveys, respectively. We carefully controlled the quality of the individual datasets, harmonised the definitions and coding of the variables and compiled them into a combined dataset for statistical analysis.

All analyses incorporated survey weights and individual study weights.

Survey weights were constructed to adjust for the following aspects of the STEPS survey and DHS: (i) the probability of selection of households induced by the sampling scheme (sample weight), (ii) the non-response (non-response weight), and (iii) the differences between the sample population and target population (population weight). A detailed explanation of the weighting procedures can be found in the WHO STEPS Surveillance Manual,[1] and the DHS Methodology report.[2]

Individual study weights were further calculated and incorporated in the pooled analysis to account for the contribution of each study to the overall analysis. The weight assigned to each study was proportional to the inverse of its variance (or standard error).

Spatial and spatio-temporal models with R-INLA

Trends over time in mean systolic blood pressure (SBP) and mean diastolic blood pressure (DBP), as well as hypertension-related outcomes including prevalence, awareness, treatment, and control, were estimated.

The model utilizes a multivariate regression framework to estimate SBP, DBP, and hypertension-related outcomes across different countries and years, from 2003 to 2022, incorporating both spatial (latitude and longitude) and temporal (year) covariates using a spatio-temporal model.[3,4] Spatial correlation was modelled using country-level random effects with a conditional autoregressive (CAR) structure,[3] so that geographically proximate countries exhibit more similar outcomes than distant ones. Temporal trends beyond discrete year-specific random effects were modelled using a first-order autoregressive (AR(1)) process, capturing smooth changes over time beyond the random effects of individual survey years. Covariates used for imputations incorporates country-level data of World Bank and United Nations Development Programme (UNDP) such as Food Production Index, GDP per capita, grams of fat per day per capita, prevalence of undernourishment, employment in agriculture, value added in agriculture, and urban population.[5,6] BP and hypertension-related outcomes values were not only estimated for missing years and/or countries within the initially studied 37 countries, but also, the spatio-temporal analysis enabled us to extend estimates to the 10 African countries for which data were unavailable or STEPS were not conducted, thereby providing comprehensive trends for all 47 member states in the African region. All missing values were imputed 10 times, generating multiple distinct complete datasets. Final estimates were obtained by averaging across imputations, accounting for random variation to ensure robustness in the final inferences and minimize bias.

The analysis involved three major steps: first, missing World Bank and UNDP covariate data were handled using the Amelia multiple imputations package.[7] To ensure consistency and comparability, numeric UNDP and world bank covariates were normalized, and missing values were imputed using the mean of the available data. Second, the most relevant World Bank and UNDP covariates for predicting SBP, DBP, hypertension prevalence, awareness, treatment, and control were identified using stepwise selection based on the Akaike Information Criterion (AIC). Finally, trends over the 20-year period (2003 to 2022) in mean aggregated SBP, DBP, and in the prevalence of hypertension-related outcomes were estimated using a spatio-temporal Bayesian model defined using INLA, which is computationally efficient and provides accurate approximations for Bayesian inference.[8] This method allows for the inclusion of complex random effects structures to capture spatial and temporal dependencies. The Besag-York-Mollié (BYM) model accounts for structured and unstructured spatial variation, while independent and identically distributed random effects handle temporal components.[9] INLA's flexibility, efficiency, and robustness make it well-suited for analysing the spatial and temporal trends in BP and hypertension-related outcomes, providing reliable parameter estimates and uncertainty quantification.[4] All estimations were provided by 95% credible interval. To assess the statistical significance of the trends, we fitted separate unadjusted linear regression models with the mean aggregated SBP, DBP, and hypertension-related outcomes as the dependent variables, and year as the independent variable. We then extracted the p-values associated with the regression coefficient for year.

Supplemental Table 1. Data sources used in the analysis

Country	AFRO*	Survey‡	Year of survey	Geographic coverage	Rural, urban, or both	Country income level	Age range		Sample size	
							Females	Males	Females	Males
Algeria	North Africa	STEPS	2003	Subnational	Both	Upper-Middle	25-64	25-64	2253	1441
Algeria	North Africa	STEPS	2016-2017	National	Both	Upper-Middle	18-69	18-69	3770	2954
Benin	West Africa	STEPS	2007	Community	Urban	Low	25-64	25-64	1525	954
Benin	West Africa	STEPS	2008	National	Both	Low	25-64	25-64	3384	3439
Benin	West Africa	DHS	2011-2012	National	Both	Low	18-49	26-64	4510	2628
Benin	West Africa	STEPS	2015	National	Both	Low	18-69	18-69	2782	2309
Benin	West Africa	DHS	2017-1018	National	Both	Low	18-49	30-64	3063	3637
Botswana	Southern Africa	STEPS	2007	National	Both	Upper-Middle	25-64	25-64	2608	1230
Botswana	Southern Africa	STEPS	2014	National	Both	Upper-Middle	18-69	18-69	2604	1237
Burkina Faso	West Africa	STEPS	2013	National	Both	Low	25-64	25-64	2401	2231
Cabo Verde	West Africa	STEPS	2007	National	Both	Lower-Middle	25-64	25-64	1094	663
Cabo Verde	West Africa	STEPS	2020	National	Both	Lower-Middle	18-69	18-69	2712	1835
Cameroon	Central Africa	STEPS	2003	Subnational	Urban	Low	18-64	18-64	4901	3235
Central African Republic	Central Africa	STEPS	2010	Subnational	Both	Low	25-64	25-64	2135	1861
Central African Republic	Central Africa	STEPS	2017	Subnational	Both	Low	25-64	25-64	2071	1201
Chad	Central Africa	STEPS	2008	Community	Urban	Low	25-64	25-64	287	394
Comoros	Eastern Africa	STEPS	2011	National	Both	Low	25-64	25-64	3869	1565
Congo	Central Africa	STEPS	2004	Community	Urban	Low	25-64	25-64	962	1009
Côte d'Ivoire	West Africa	STEPS	2005	Subnational	Urban	Low	15-69	15-69	2398	1872
Democratic Republic of the Congo	Central Africa	STEPS	2005	Subnational	Urban	Low	25-64	25-64	962	1009
Eritrea	Eastern Africa	STEPS	2004	National	Both	Low	18-64	18-65	296	260
Eritrea	Eastern Africa	STEPS	2010	National	Both	Low	25-69	25-69	4358	1534
Eswatini	Southern Africa	STEPS	2007	National	Both	Lower-Middle	25-64	25-64	832	433
Eswatini	Southern Africa	STEPS	2014	National	Both	Lower-Middle	18-69	18-69	1941	994
Ethiopia	Eastern Africa	STEPS	2006	Subnational	Urban	Low	25-64	25-64	2329	1637

Ethiopia	Eastern Africa	STEPS	2015	National	Both	Low	18-69	18-69	5429	3702
Gabon	Central Africa	STEPS	2009	Subnational	Urban	Upper-Middle	18-64	18-64	1561	1014
Gambia	West Africa	STEPS	2010	National	Both	Low	25-64	25-64	2170	1633
Ghana	West Africa	STEPS	2006	Community	Urban	Low	25-64	25-64	1735	881
Ghana	West Africa	DHS	2014	National	Both	Low	18-49	18-59	8264	3848
Guinea	West Africa	STEPS	2009	Subnational	Both	Low	18-64	18-64	1228	1057
Kenya	Eastern Africa	STEPS	2015	National	Both	Lower-Middle	18-69	18-69	2658	1760
Lesotho	Southern Africa	DHS	2009-2010	National	Both	Lower-Middle	18-49	18-59	3292	2643
Lesotho	Southern Africa	STEPS	2012	National	Both	Lower-Middle	25-64	25-64	1497	743
Lesotho	Southern Africa	DHS	2014	National	Both	Lower-Middle	18-49	18-59	2757	2345
Liberia	West Africa	STEPS	2011	National	Both	Low	25-64	25-64	1379	1015
Liberia	West Africa	STEPS	2022	National	Both	Low	18-69	18-69	2531	1528
Madagascar	Eastern Africa	STEPS	2005	Subnational	Both	Low	25-64	25-64	1451	1381
Malawi	Eastern Africa	STEPS	2009	National	Both	Low	25-64	25-64	2726	1183
Malawi	Eastern Africa	STEPS	2017	National	Both	Low	18-69	18-69	2692	1485
Mali	West Africa	STEPS	2007	Subnational	Both	Low	18-64	18-64	1265	837
Mali	West Africa	STEPS	2013	Subnational	Both	Low	18-65	18-66	1084	667
Mozambique	Eastern Africa	STEPS	2005	National	Both	Low	25-64	25-64	429	313
Namibia	Southern Africa	DHS	2013	National	Both	Lower-Middle	35-64	35-64	2076	1525
Niger	West Africa	STEPS	2007	National	Both	Low	18-64	18-64	1222	1372
Niger	West Africa	STEPS	2021	National	Both	Low	18-69	18-69	3419	2273
Rwanda	Eastern Africa	STEPS	2012	National	Both	Low	18-64	18-64	4217	2492
Rwanda	Eastern Africa	STEPS	2021	National	Both	Low	18-69	18-69	3540	2125
Sao Tome and Principe	Central Africa	STEPS	2008	National	Both	Lower-Middle	25-64	25-64	1400	1043
Sao Tome and Principe	Central Africa	STEPS	2019	National	Both	Lower-Middle	18-69	18-69	1437	978
Seychelles	Eastern Africa	STEPS	2004	National	Both	Upper-Middle	25-64	25-64	687	568
Sierra Leone	West Africa	STEPS	2009	National	Both	Low	25-64	25-64	2606	2207
South Africa	Southern Africa	DHS	2016	National	Both	Upper-Middle	18-69	18-69	4229	2778
Togo	West Africa	STEPS	2010	National	Both	Low	18-64	18-64	2091	1894

Togo	West Africa	STEPS	2021	National	Both	Low	18-69	18-69	2288	1585
Uganda	Eastern Africa	STEPS	2014	National	Both	Low	18-69	18-69	2336	1570
United Republic of Tanzania	Eastern Africa	DHS	2011	National	Both	Low	25-64	25-64	1607	1008
United Republic of Tanzania	Eastern Africa	STEPS	2012	National	Both	Low	25-64	25-64	3006	2576
United Republic of Tanzania	Eastern Africa	STEPS	2022	National	Both	Low	18-49	18-49	6547	4745
Zambia	Eastern Africa	STEPS	2008	National	Both	Lower-Middle	25-68	25-69	1230	608
Zambia	Eastern Africa	STEPS	2017	National	Both	Lower-Middle	18-69	18-69	2592	1554
*AFRO: World Health Organization African Region. ‡STEPS: STEPwise approach to noncommunicable diseases risk factor Surveillance and DHS: Demographic and Health Survey.										

Supplemental Table 2. Rationale for Non-Inclusion of STEPS Surveys

		Reason for not included
1	Angola	Official STEPS survey not conducted
-	Burkina Faso, 2021	No access to STEPS survey data
2	Burundi	Official STEPS survey not conducted
3	Equatorial Guinea	Official STEPS survey not conducted
4	Guinea Bissau	Official STEPS survey not conducted
5	Mauritania, 2006	No blood pressure measurement
6	Mauritius	Official STEPS survey not conducted
7	Namibia, 2005	Too much missing of STEPS survey for blood pressure measurements (≥75%)
8	Nigeria	Official STEPS survey not conducted
9	Senegal, 2015	No access to STEPS survey data
10	South Africa	Official STEPS survey not conducted
11	South Sudan	Official STEPS survey not conducted
12	Zimbabwe	Official STEPS survey not conducted

Supplemental Table 3a. Distribution of United Nations Population for the World Health Organization region by year and sex

Year of survey	Sample size	
	Males	Females
2003	350 401 644	356 074 534
2004	359 885 823	365 420 096
2005	369 662 217	375 066 725
2006	379 776 876	385 041 717
2007	390 275 156	395 384 802
2008	401 129 582	406 098 587
2009	412 306 251	417 169 219
2010	423 868 068	428 621 373
2011	435 838 290	440 467 162
2012	448 050 328	452 558 986
2013	460 497 810	464 871 247
2014	473 175 202	477 380 009
2015	486 232 066	490 148 665
2016	499 303 234	503 065 566
2017	512 386 745	516 101 474
2018	525 902 580	529 479 834
2019	539 736 084	543 204 116
2020	553 864 262	557 236 455
2021	568 028 688	571 337 149
2022	582 270 420	585 518 658

Supplemental Table 3b. Distribution of United Nations Population for the World Health Organization region by year, country, and sex

Year of survey	Country	Sample size	
		Males	Females
2003	Algeria	16 019 236	15 395 531
2003	Angola	8 726 610	9 035 245
2003	Benin	3 727 571	3 778 452
2003	Botswana	871 941	918 384
2003	Burkina Faso	6 312 496	6 457 484
2003	Burundi	3 312 914	3 410 716
2003	Cabo Verde	230 602	239 885
2003	Cameroon	7 959 123	8 068 117
2003	Central African Republic	1 981 950	1 964 355
2003	Chad	4 496 907	4 515 531
2003	Comoros	277 486	281 241
2003	Congo	1 664 621	1 691 539
2003	Côte d'Ivoire	8 950 606	8 803 517
2003	Democratic Republic of the Congo	25 784 021	26 357 506
2003	Equatorial Guinea	395 595	378 293
2003	Eritrea	1 272 837	1 327 486
2003	Eswatini	499 439	538 182
2003	Ethiopia	36 025 474	35 679 988
2003	Gabon	674 411	676 419
2003	Gambia	755 948	778 983
2003	Ghana	10 389 169	10 513 754
2003	Guinea	4 217 476	4 379 334
2003	Guinea-Bissau	629 361	659 979
2003	Kenya	16 423 605	16 668 173
2003	Lesotho	955 239	997 930
2003	Liberia	1 489 412	1 534 057
2003	Madagascar	8 727 905	8 641 919
2003	Malawi	5 742 423	6 103 783
2003	Mali	6 107 976	5 987 345
2003	Mauritania	1 408 388	1 417 271
2003	Mauritius	592 988	596 114
2003	Mozambique	9 152 399	9 650 621
2003	Namibia	909 484	967 632
2003	Niger	6 380 723	6 262 050
2003	Nigeria	65 607 923	64 849 481
2003	Rwanda	4 028 211	4 368 422
2003	Sao Tome and Principe	75 147	75 539
2003	Senegal	5 036 865	5 188 949
2003	Seychelles	41 011	40 114
2003	Sierra Leone	2 608 232	2 635 658
2003	South Africa	22 466 875	24 675 092
2003	South Sudan	3 342 463	3 510 056
2003	Togo	2 649 905	2 662 675
2003	Uganda	12 653 233	13 174 409

2003	United Republic of Tanzania	18 077 450	18 509 788
2003	Zambia	5 139 991	5 481 222
2003	Zimbabwe	5 608 000	6 226 312
2004	Algeria	16 246 576	15 613 406
2004	Angola	9 041 583	9 354 120
2004	Benin	3 844 784	3 891 879
2004	Botswana	888 394	933 511
2004	Burkina Faso	6 522 505	6 654 553
2004	Burundi	3 440 289	3 537 797
2004	Cabo Verde	234 372	242 479
2004	Cameroon	8 182 844	8 290 375
2004	Central African Republic	2 025 067	2 007 768
2004	Chad	4 704 254	4 716 979
2004	Comoros	283 019	286 511
2004	Congo	1 723 576	1 748 576
2004	Côte d’Ivoire	9 169 463	9 004 542
2004	Democratic Republic of the Congo	26 569 479	27 149 816
2004	Equatorial Guinea	415 493	394 335
2004	Eritrea	1 326 855	1 381 022
2004	Eswatini	502 640	541 809
2004	Ethiopia	37 095 546	36 699 459
2004	Gabon	694 298	694 470
2004	Gambia	778 593	801 387
2004	Ghana	10 674 555	10 793 760
2004	Guinea	4 307 664	4 474 154
2004	Guinea-Bissau	644 754	675 315
2004	Kenya	16 928 707	17 167 292
2004	Lesotho	951 543	994 134
2004	Liberia	1 507 815	1 552 183
2004	Madagascar	8 986 526	8 899 233
2004	Malawi	5 889 571	6 273 544
2004	Mali	6 311 368	6 185 588
2004	Mauritania	1 436 724	1 450 920
2004	Mauritius	596 432	600 151
2004	Mozambique	9 394 820	9 905 702
2004	Namibia	921 020	979 598
2004	Niger	6 615 277	6 484 269
2004	Nigeria	67 409 393	66 612 318
2004	Rwanda	4 140 321	4 475 694
2004	Sao Tome and Principe	77 083	77 459
2004	Senegal	5 162 580	5 322 137
2004	Seychelles	40 975	39 849
2004	Sierra Leone	2 698 966	2 723 696
2004	South Africa	22 713 050	24 871 899
2004	South Sudan	3 504 144	3 666 632
2004	Togo	2 721 642	2 732 271
2004	Uganda	13 042 055	13 561 108

2004	United Republic of Tanzania	18 564 140	19 029 521
2004	Zambia	5 315 143	5 649 137
2004	Zimbabwe	5 639 926	6 277 738
2005	Algeria	16 470 288	15 827 269
2005	Angola	9 372 909	9 689 030
2005	Benin	3 969 765	4 016 665
2005	Botswana	905 576	949 375
2005	Burkina Faso	6 739 187	6 859 417
2005	Burundi	3 572 029	3 669 067
2005	Cabo Verde	238 016	244 956
2005	Cameroon	8 411 929	8 517 738
2005	Central African Republic	2 070 868	2 053 790
2005	Chad	4 899 057	4 905 855
2005	Comoros	289 023	291 807
2005	Congo	1 788 147	1 811 234
2005	Côte d’Ivoire	9 388 305	9 202 506
2005	Democratic Republic of the Congo	27 417 443	28 001 799
2005	Equatorial Guinea	436 243	411 188
2005	Eritrea	1 360 674	1 414 424
2005	Eswatini	505 488	544 960
2005	Ethiopia	38 181 785	37 738 757
2005	Gabon	715 659	713 527
2005	Gambia	802 313	824 847
2005	Ghana	10 966 591	11 080 421
2005	Guinea	4 390 280	4 567 031
2005	Guinea-Bissau	660 831	691 288
2005	Kenya	17 446 692	17 679 458
2005	Lesotho	947 640	990 235
2005	Liberia	1 578 166	1 622 826
2005	Madagascar	9 252 141	9 164 187
2005	Malawi	6 046 846	6 453 690
2005	Mali	6 523 805	6 393 135
2005	Mauritania	1 466 339	1 485 775
2005	Mauritius	599 665	604 024
2005	Mozambique	9 641 872	10 165 021
2005	Namibia	932 194	991 413
2005	Niger	6 860 396	6 717 722
2005	Nigeria	69 257 269	68 423 637
2005	Rwanda	4 257 139	4 588 634
2005	Sao Tome and Principe	79 037	79 410
2005	Senegal	5 293 935	5 460 641
2005	Seychelles	41 272	39 929
2005	Sierra Leone	2 774 076	2 795 591
2005	South Africa	22 964 627	25 072 177
2005	South Sudan	3 676 090	3 833 312
2005	Togo	2 794 609	2 802 756
2005	Uganda	13 436 266	13 951 390

2005	United Republic of Tanzania	19 076 412	19 574 303
2005	Zambia	5 504 875	5 828 697
2005	Zimbabwe	5 658 447	6 321 810
2006	Algeria	16 709 905	16 056 475
2006	Angola	9 719 600	10 039 493
2006	Benin	4 094 057	4 140 523
2006	Botswana	923 958	966 171
2006	Burkina Faso	6 959 132	7 070 785
2006	Burundi	3 704 283	3 800 742
2006	Cabo Verde	241 564	247 341
2006	Cameroon	8 646 274	8 750 031
2006	Central African Republic	2 112 595	2 095 871
2006	Chad	5 078 647	5 079 654
2006	Comoros	295 210	297 355
2006	Congo	1 857 926	1 879 129
2006	Côte d’Ivoire	9 607 557	9 398 618
2006	Democratic Republic of the Congo	28 312 744	28 901 254
2006	Equatorial Guinea	458 125	429 184
2006	Eritrea	1 384 577	1 437 913
2006	Eswatini	508 161	548 018
2006	Ethiopia	39 291 262	38 805 967
2006	Gabon	738 691	733 794
2006	Gambia	827 419	849 649
2006	Ghana	11 264 297	11 372 318
2006	Guinea	4 478 965	4 665 048
2006	Guinea-Bissau	677 747	708 063
2006	Kenya	17 979 114	18 207 634
2006	Lesotho	947 131	990 114
2006	Liberia	1 670 578	1 715 711
2006	Madagascar	9 526 068	9 437 225
2006	Malawi	6 213 281	6 642 660
2006	Mali	6 743 303	6 607 769
2006	Mauritania	1 497 574	1 522 030
2006	Mauritius	602 131	607 184
2006	Mozambique	9 892 653	10 428 610
2006	Namibia	943 367	1 003 460
2006	Niger	7 116 110	6 961 755
2006	Nigeria	71 158 979	70 284 190
2006	Rwanda	4 378 002	4 706 663
2006	Sao Tome and Principe	81 013	81 397
2006	Senegal	5 432 042	5 606 077
2006	Seychelles	42 247	40 660
2006	Sierra Leone	2 837 850	2 855 728
2006	South Africa	23 222 321	25 279 601
2006	South Sudan	3 858 570	4 010 357
2006	Togo	2 875 708	2 881 048
2006	Uganda	13 842 864	14 354 898

2006	United Republic of Tanzania	19 609 929	20 140 881
2006	Zambia	5 710 845	6 021 292
2006	Zimbabwe	5 702 499	6 381 381
2007	Algeria	16 983 857	16 320 294
2007	Angola	10 083 720	10 407 771
2007	Benin	4 216 597	4 258 209
2007	Botswana	943 660	983 978
2007	Burkina Faso	7 176 887	7 285 046
2007	Burundi	3 845 147	3 940 571
2007	Cabo Verde	244 996	249 642
2007	Cameroon	8 892 508	8 994 320
2007	Central African Republic	2 152 162	2 135 895
2007	Chad	5 256 304	5 251 972
2007	Comoros	301 512	303 050
2007	Congo	1 928 884	1 948 318
2007	Côte d’Ivoire	9 827 536	9 593 811
2007	Democratic Republic of the Congo	29 245 935	29 837 698
2007	Equatorial Guinea	481 310	448 528
2007	Eritrea	1 407 410	1 460 233
2007	Eswatini	510 992	551 336
2007	Ethiopia	40 438 652	39 917 608
2007	Gabon	763 453	755 324
2007	Gambia	853 911	875 674
2007	Ghana	11 565 731	11 668 423
2007	Guinea	4 582 997	4 773 142
2007	Guinea-Bissau	695 729	725 831
2007	Kenya	18 525 353	18 750 706
2007	Lesotho	950 953	992 844
2007	Liberia	1 757 525	1 802 561
2007	Madagascar	9 807 626	9 718 832
2007	Malawi	6 388 201	6 837 353
2007	Mali	6 969 942	6 829 351
2007	Mauritania	1 530 640	1 559 797
2007	Mauritius	604 493	610 344
2007	Mozambique	10 153 678	10 701 226
2007	Namibia	954 980	1 016 283
2007	Niger	7 382 977	7 216 938
2007	Nigeria	73 128 487	72 199 662
2007	Rwanda	4 503 033	4 829 671
2007	Sao Tome and Principe	83 027	83 422
2007	Senegal	5 575 406	5 757 184
2007	Seychelles	42 576	40 755
2007	Sierra Leone	2 903 035	2 917 345
2007	South Africa	23 492 665	25 503 507
2007	South Sudan	4 051 449	4 198 017
2007	Togo	2 962 129	2 964 457
2007	Uganda	14 264 043	14 773 165

2007	United Republic of Tanzania	20 158 690	20 723 477
2007	Zambia	5 929 318	6 224 713
2007	Zimbabwe	5 755 039	6 446 517
2008	Algeria	17 275 989	16 602 210
2008	Angola	10 464 952	10 792 741
2008	Benin	4 345 506	4 382 833
2008	Botswana	964 353	1 002 819
2008	Burkina Faso	7 391 975	7 501 981
2008	Burundi	4 008 966	4 103 580
2008	Cabo Verde	248 302	251 828
2008	Cameroon	9 150 818	9 250 722
2008	Central African Republic	2 196 855	2 181 033
2008	Chad	5 443 189	5 433 502
2008	Comoros	307 960	308 920
2008	Congo	1 995 012	2 012 797
2008	Côte d'Ivoire	10 049 312	9 790 248
2008	Democratic Republic of the Congo	30 205 046	30 799 684
2008	Equatorial Guinea	505 817	469 254
2008	Eritrea	1 446 721	1 498 942
2008	Eswatini	514 557	553 517
2008	Ethiopia	41 609 028	41 060 935
2008	Gabon	789 852	778 127
2008	Gambia	881 504	902 627
2008	Ghana	11 871 026	11 968 538
2008	Guinea	4 695 970	4 888 219
2008	Guinea-Bissau	714 400	744 263
2008	Kenya	19 090 068	19 313 089
2008	Lesotho	957 361	997 753
2008	Liberia	1 831 970	1 876 240
2008	Madagascar	10 095 965	10 007 362
2008	Malawi	6 576 254	7 035 380
2008	Mali	7 203 043	7 057 052
2008	Mauritania	1 565 490	1 603 179
2008	Mauritius	606 245	612 993
2008	Mozambique	10 423 508	10 985 150
2008	Namibia	967 505	1 030 275
2008	Niger	7 662 020	7 484 052
2008	Nigeria	75 165 025	74 169 830
2008	Rwanda	4 630 555	4 955 801
2008	Sao Tome and Principe	85 064	85 460
2008	Senegal	5 722 774	5 912 697
2008	Seychelles	43 663	41 555
2008	Sierra Leone	2 978 940	2 990 103
2008	South Africa	23 792 526	25 761 970
2008	South Sudan	4 252 884	4 394 527
2008	Togo	3 049 502	3 048 530
2008	Uganda	14 697 260	15 202 404

2008	United Republic of Tanzania	20 703 170	21 310 295
2008	Zambia	6 157 943	6 437 963
2008	Zimbabwe	5 793 736	6 505 606
2009	Algeria	17 588 002	16 904 114
2009	Angola	10 863 010	11 194 510
2009	Benin	4 477 578	4 511 486
2009	Botswana	985 562	1 022 456
2009	Burkina Faso	7 612 014	7 725 007
2009	Burundi	4 220 595	4 314 583
2009	Cabo Verde	251 467	253 858
2009	Cameroon	9 417 498	9 515 390
2009	Central African Republic	2 244 275	2 228 974
2009	Chad	5 640 772	5 625 433
2009	Comoros	314 609	315 034
2009	Congo	2 077 924	2 094 161
2009	Côte d'Ivoire	10 274 205	9 990 001
2009	Democratic Republic of the Congo	31 192 072	31 792 755
2009	Equatorial Guinea	531 561	491 251
2009	Eritrea	1 485 273	1 536 937
2009	Eswatini	518 727	554 262
2009	Ethiopia	42 793 943	42 226 530
2009	Gabon	818 078	802 394
2009	Gambia	910 043	930 514
2009	Ghana	12 179 615	12 272 131
2009	Guinea	4 813 342	5 007 555
2009	Guinea-Bissau	733 497	763 154
2009	Kenya	19 666 982	19 890 174
2009	Lesotho	964 636	1 004 350
2009	Liberia	1 891 969	1 934 995
2009	Madagascar	10 391 819	10 302 931
2009	Malawi	6 775 734	7 237 220
2009	Mali	7 441 793	7 290 190
2009	Mauritania	1 603 011	1 653 152
2009	Mauritius	607 410	615 069
2009	Mozambique	10 704 989	11 282 938
2009	Namibia	981 083	1 045 478
2009	Niger	7 953 842	7 763 315
2009	Nigeria	77 269 100	76 194 742
2009	Rwanda	4 759 253	5 083 609
2009	Sao Tome and Principe	87 083	87 483
2009	Senegal	5 876 326	6 074 802
2009	Seychelles	43 964	41 589
2009	Sierra Leone	3 063 232	3 071 413
2009	South Africa	24 107 916	26 039 448
2009	South Sudan	4 453 860	4 590 783
2009	Togo	3 137 509	3 133 142
2009	Uganda	15 141 453	15 642 817

2009	United Republic of Tanzania	21 200 229	21 878 546
2009	Zambia	6 392 918	6 658 807
2009	Zimbabwe	5 846 477	6 579 736
2010	Algeria	17 916 983	17 222 234
2010	Angola	11 280 443	11 616 459
2010	Benin	4 613 122	4 643 673
2010	Botswana	1 007 080	1 042 751
2010	Burkina Faso	7 839 504	7 955 003
2010	Burundi	4 425 712	4 518 362
2010	Cabo Verde	254 402	256 386
2010	Cameroon	9 692 319	9 788 157
2010	Central African Republic	2 290 762	2 276 104
2010	Chad	5 838 991	5 817 842
2010	Comoros	321 482	321 421
2010	Congo	2 167 099	2 182 027
2010	Côte d’Ivoire	10 503 606	10 194 035
2010	Democratic Republic of the Congo	32 227 491	32 835 941
2010	Equatorial Guinea	558 336	514 298
2010	Eritrea	1 516 786	1 567 986
2010	Eswatini	522 895	555 028
2010	Ethiopia	44 018 161	43 434 873
2010	Gabon	848 505	828 378
2010	Gambia	939 391	959 139
2010	Ghana	12 487 882	12 575 341
2010	Guinea	4 934 651	5 130 662
2010	Guinea-Bissau	753 201	782 676
2010	Kenya	20 230 037	20 457 500
2010	Lesotho	971 918	1 010 375
2010	Liberia	1 948 951	1 990 604
2010	Madagascar	10 692 733	10 603 699
2010	Malawi	6 980 128	7 443 925
2010	Mali	7 687 875	7 530 723
2010	Mauritania	1 644 359	1 706 713
2010	Mauritius	608 447	616 945
2010	Mozambique	11 011 497	11 600 752
2010	Namibia	995 663	1 061 621
2010	Niger	8 259 092	8 055 500
2010	Nigeria	79 445 128	78 288 669
2010	Rwanda	4 889 445	5 213 405
2010	Sao Tome and Principe	89 041	89 455
2010	Senegal	6 036 031	6 243 489
2010	Seychelles	45 358	42 617
2010	Sierra Leone	3 151 315	3 156 649
2010	South Africa	24 428 957	26 320 266
2010	South Sudan	4 693 997	4 826 133
2010	Togo	3 224 157	3 216 261
2010	Uganda	15 598 531	16 096 363

2010	United Republic of Tanzania	21 732 543	22 475 775
2010	Zambia	6 630 580	6 885 665
2010	Zimbabwe	5 913 482	6 669 493
2011	Algeria	18 259 535	17 553 135
2011	Angola	11 716 549	12 057 381
2011	Benin	4 752 420	4 779 434
2011	Botswana	1 028 186	1 063 171
2011	Burkina Faso	8 076 772	8 193 826
2011	Burundi	4 587 827	4 678 791
2011	Cabo Verde	257 362	259 610
2011	Cameroon	9 973 115	10 066 781
2011	Central African Republic	2 325 729	2 311 652
2011	Chad	6 049 000	6 022 375
2011	Comoros	328 579	328 090
2011	Congo	2 239 351	2 253 181
2011	Côte d’Ivoire	10 725 413	10 406 243
2011	Democratic Republic of the Congo	33 332 791	33 948 393
2011	Equatorial Guinea	585 191	536 506
2011	Eritrea	1 546 294	1 597 125
2011	Eswatini	527 111	556 154
2011	Ethiopia	45 291 289	44 690 282
2011	Gabon	881 035	856 015
2011	Gambia	969 657	988 591
2011	Ghana	12 799 679	12 882 143
2011	Guinea	5 059 991	5 257 167
2011	Guinea-Bissau	773 786	803 049
2011	Kenya	20 772 035	21 010 406
2011	Lesotho	979 976	1 016 948
2011	Liberia	2 028 737	2 068 790
2011	Madagascar	10 994 882	10 906 312
2011	Malawi	7 188 132	7 655 040
2011	Mali	7 940 775	7 778 164
2011	Mauritania	1 689 815	1 763 949
2011	Mauritius	609 069	618 286
2011	Mozambique	11 345 988	11 939 224
2011	Namibia	1 011 086	1 078 608
2011	Niger	8 577 165	8 360 285
2011	Nigeria	81 697 350	80 457 121
2011	Rwanda	5 020 596	5 344 797
2011	Sao Tome and Principe	90 972	91 352
2011	Senegal	6 200 749	6 417 613
2011	Seychelles	44 341	41 351
2011	Sierra Leone	3 238 772	3 241 366
2011	South Africa	24 773 330	26 621 129
2011	South Sudan	4 955 153	5 083 035
2011	Togo	3 312 740	3 300 959
2011	Uganda	16 067 940	16 561 882

2011	United Republic of Tanzania	22 355 193	23 132 519
2011	Zambia	6 865 004	7 115 494
2011	Zimbabwe	5 991 829	6 773 441
2012	Algeria	18 616 070	17 899 281
2012	Angola	12 169 199	12 515 328
2012	Benin	4 895 676	4 918 120
2012	Botswana	1 048 602	1 083 316
2012	Burkina Faso	8 328 138	8 443 319
2012	Burundi	4 755 150	4 844 418
2012	Cabo Verde	260 411	262 775
2012	Cameroon	10 260 481	10 351 549
2012	Central African Republic	2 345 681	2 332 158
2012	Chad	6 265 825	6 233 983
2012	Comoros	335 863	334 999
2012	Congo	2 303 161	2 315 830
2012	Côte d’Ivoire	10 941 825	10 628 672
2012	Democratic Republic of the Congo	34 477 894	35 100 018
2012	Equatorial Guinea	612 010	557 752
2012	Eritrea	1 568 531	1 619 013
2012	Eswatini	531 560	557 655
2012	Ethiopia	46 589 674	45 972 579
2012	Gabon	915 091	884 880
2012	Gambia	1 000 775	1 019 019
2012	Ghana	13 120 680	13 200 908
2012	Guinea	5 187 018	5 385 900
2012	Guinea-Bissau	795 341	824 322
2012	Kenya	21 298 906	21 552 384
2012	Lesotho	989 167	1 024 457
2012	Liberia	2 103 354	2 141 751
2012	Madagascar	11 297 224	11 209 691
2012	Malawi	7 399 250	7 870 376
2012	Mali	8 176 180	8 008 213
2012	Mauritania	1 738 999	1 824 392
2012	Mauritius	610 457	620 308
2012	Mozambique	11 702 891	12 294 969
2012	Namibia	1 027 415	1 096 704
2012	Niger	8 913 043	8 682 276
2012	Nigeria	83 999 524	82 674 889
2012	Rwanda	5 150 620	5 472 907
2012	Sao Tome and Principe	92 897	93 228
2012	Senegal	6 370 261	6 596 935
2012	Seychelles	44 934	41 604
2012	Sierra Leone	3 326 437	3 326 380
2012	South Africa	25 138 362	26 943 770
2012	South Sudan	5 181 653	5 305 919
2012	Togo	3 401 924	3 386 178
2012	Uganda	16 548 455	17 039 375

2012	United Republic of Tanzania	23 013 814	23 816 600
2012	Zambia	7 100 575	7 349 189
2012	Zimbabwe	6 099 327	6 900 697
2013	Algeria	18 983 673	18 256 941
2013	Angola	12 636 184	12 987 879
2013	Benin	5 042 707	5 059 849
2013	Botswana	1 069 129	1 103 803
2013	Burkina Faso	8 585 478	8 698 202
2013	Burundi	4 929 474	5 017 112
2013	Cabo Verde	263 343	265 799
2013	Cameroon	10 556 042	10 644 151
2013	Central African Republic	2 359 365	2 347 016
2013	Chad	6 494 578	6 457 853
2013	Comoros	343 292	342 113
2013	Congo	2 359 922	2 371 583
2013	Côte d'Ivoire	11 163 608	10 856 274
2013	Democratic Republic of the Congo	35 680 641	36 310 180
2013	Equatorial Guinea	639 437	579 625
2013	Eritrea	1 590 196	1 640 244
2013	Eswatini	536 328	559 626
2013	Ethiopia	47 884 785	47 257 893
2013	Gabon	949 777	914 405
2013	Gambia	1 032 423	1 049 949
2013	Ghana	13 448 446	13 526 639
2013	Guinea	5 316 814	5 517 508
2013	Guinea-Bissau	817 562	846 235
2013	Kenya	21 814 076	22 082 445
2013	Lesotho	999 384	1 033 075
2013	Liberia	2 151 021	2 187 745
2013	Madagascar	11 600 827	11 515 484
2013	Malawi	7 613 952	8 090 327
2013	Mali	8 418 406	8 245 547
2013	Mauritania	1 788 521	1 879 579
2013	Mauritius	611 636	622 113
2013	Mozambique	12 079 517	12 667 179
2013	Namibia	1 044 627	1 115 793
2013	Niger	9 262 560	9 017 575
2013	Nigeria	86 319 313	84 912 288
2013	Rwanda	5 280 946	5 598 377
2013	Sao Tome and Principe	94 785	95 097
2013	Senegal	6 543 148	6 780 505
2013	Seychelles	45 910	42 240
2013	Sierra Leone	3 414 030	3 411 531
2013	South Africa	25 515 399	27 280 745
2013	South Sudan	5 381 613	5 502 297
2013	Togo	3 491 883	3 472 221
2013	Uganda	17 039 471	17 528 628

2013	United Republic of Tanzania	23 725 904	24 542 666
2013	Zambia	7 341 800	7 588 475
2013	Zimbabwe	6 235 879	7 048 433
2014	Algeria	19 360 619	18 624 344
2014	Angola	13 114 326	13 471 444
2014	Benin	5 195 363	5 207 184
2014	Botswana	1 090 139	1 125 028
2014	Burkina Faso	8 848 352	8 958 094
2014	Burundi	5 099 431	5 185 584
2014	Cabo Verde	266 270	268 885
2014	Cameroon	10 884 089	10 969 505
2014	Central African Republic	2 356 411	2 346 348
2014	Chad	6 732 184	6 690 999
2014	Comoros	350 875	349 444
2014	Congo	2 417 523	2 428 441
2014	Côte d’Ivoire	11 419 027	11 116 617
2014	Democratic Republic of the Congo	36 938 016	37 576 860
2014	Equatorial Guinea	667 284	601 995
2014	Eritrea	1 603 622	1 653 334
2014	Eswatini	541 324	562 024
2014	Ethiopia	49 192 427	48 559 404
2014	Gabon	983 714	943 805
2014	Gambia	1 064 222	1 081 016
2014	Ghana	13 778 184	13 854 248
2014	Guinea	5 452 933	5 653 766
2014	Guinea-Bissau	840 058	868 385
2014	Kenya	22 314 636	22 600 589
2014	Lesotho	1 010 502	1 042 836
2014	Liberia	2 197 051	2 231 959
2014	Madagascar	11 907 185	11 824 471
2014	Malawi	7 832 539	8 315 867
2014	Mali	8 689 206	8 511 571
2014	Mauritania	1 837 530	1 928 780
2014	Mauritius	612 417	623 567
2014	Mozambique	12 468 296	13 049 635
2014	Namibia	1 062 587	1 135 554
2014	Niger	9 621 824	9 362 749
2014	Nigeria	88 640 585	87 150 850
2014	Rwanda	5 414 061	5 727 022
2014	Sao Tome and Principe	96 622	96 925
2014	Senegal	6 721 971	6 968 931
2014	Seychelles	46 733	42 798
2014	Sierra Leone	3 501 331	3 496 543
2014	South Africa	25 970 671	27 664 288
2014	South Sudan	5 434 645	5 554 373
2014	Togo	3 583 032	3 559 584
2014	Uganda	17 562 029	18 047 780

2014	United Republic of Tanzania	24 485 801	25 312 459
2014	Zambia	7 589 493	7 833 545
2014	Zimbabwe	6 378 061	7 200 576
2015	Algeria	19 749 187	19 003 105
2015	Angola	13 601 345	13 963 823
2015	Benin	5 353 744	5 360 383
2015	Botswana	1 112 105	1 146 964
2015	Burkina Faso	9 119 311	9 224 348
2015	Burundi	5 213 375	5 299 230
2015	Cabo Verde	269 183	271 939
2015	Cameroon	11 234 575	11 317 818
2015	Central African Republic	2 365 542	2 357 405
2015	Chad	6 951 260	6 906 209
2015	Comoros	358 614	356 997
2015	Congo	2 476 571	2 486 528
2015	Côte d'Ivoire	11 711 517	11 413 289
2015	Democratic Republic of the Congo	38 217 352	38 866 414
2015	Equatorial Guinea	695 300	624 732
2015	Eritrea	1 611 883	1 661 322
2015	Eswatini	546 496	564 761
2015	Ethiopia	50 529 252	49 893 205
2015	Gabon	1 015 577	972 371
2015	Gambia	1 095 974	1 112 096
2015	Ghana	14 109 417	14 184 104
2015	Guinea	5 597 507	5 795 972
2015	Guinea-Bissau	862 587	890 553
2015	Kenya	22 803 169	23 111 289
2015	Lesotho	1 022 392	1 053 759
2015	Liberia	2 243 479	2 276 604
2015	Madagascar	12 217 632	12 136 262
2015	Malawi	8 054 782	8 545 380
2015	Mali	8 966 570	8 784 078
2015	Mauritania	1 887 956	1 979 339
2015	Mauritius	612 899	624 722
2015	Mozambique	12 865 760	13 440 620
2015	Namibia	1 081 173	1 155 877
2015	Niger	9 999 048	9 726 514
2015	Nigeria	90 947 365	89 368 504
2015	Rwanda	5 550 501	5 859 598
2015	Sao Tome and Principe	98 402	98 699
2015	Senegal	6 907 325	7 161 732
2015	Seychelles	47 867	43 684
2015	Sierra Leone	3 587 711	3 580 766
2015	South Africa	26 612 309	28 146 665
2015	South Sudan	5 425 472	5 544 942
2015	Togo	3 675 452	3 648 312
2015	Uganda	18 123 687	18 604 122

2015	United Republic of Tanzania	25 343 330	26 148 636
2015	Zambia	7 840 331	8 082 933
2015	Zimbabwe	6 519 779	7 352 059
2016	Algeria	20 145 188	19 387 355
2016	Angola	14 102 368	14 469 284
2016	Benin	5 516 680	5 518 204
2016	Botswana	1 135 415	1 169 952
2016	Burkina Faso	9 395 473	9 494 515
2016	Burundi	5 299 708	5 385 553
2016	Cabo Verde	272 181	275 046
2016	Cameroon	11 578 625	11 658 774
2016	Central African Republic	2 406 562	2 399 532
2016	Chad	7 175 165	7 125 568
2016	Comoros	366 519	364 788
2016	Congo	2 537 002	2 546 085
2016	Côte d’Ivoire	12 011 943	11 717 406
2016	Democratic Republic of the Congo	39 570 857	40 231 501
2016	Equatorial Guinea	723 287	647 661
2016	Eritrea	1 624 421	1 673 562
2016	Eswatini	551 857	567 817
2016	Ethiopia	51 909 168	51 278 196
2016	Gabon	1 044 789	999 693
2016	Gambia	1 127 690	1 143 172
2016	Ghana	14 444 720	14 518 496
2016	Guinea	5 749 456	5 942 910
2016	Guinea-Bissau	885 124	912 737
2016	Kenya	23 304 452	23 632 326
2016	Lesotho	1 035 168	1 065 826
2016	Liberia	2 290 311	2 321 665
2016	Madagascar	12 536 047	12 455 855
2016	Malawi	8 280 097	8 777 413
2016	Mali	9 256 991	9 069 113
2016	Mauritania	1 939 676	2 031 177
2016	Mauritius	612 983	625 488
2016	Mozambique	13 289 996	13 852 567
2016	Namibia	1 100 184	1 176 701
2016	Niger	10 394 527	10 108 781
2016	Nigeria	93 288 140	91 605 452
2016	Rwanda	5 693 265	5 999 016
2016	Sao Tome and Principe	100 125	100 416
2016	Senegal	7 097 766	7 358 564
2016	Seychelles	48 587	44 197
2016	Sierra Leone	3 676 453	3 667 581
2016	South Africa	26 869 802	28 424 026
2016	South Sudan	5 363 477	5 481 306
2016	Togo	3 769 436	3 738 690
2016	Uganda	18 748 611	19 224 722

2016	United Republic of Tanzania	26 276 958	27 036 807
2016	Zambia	8 095 262	8 337 143
2016	Zimbabwe	6 660 725	7 502 926
2017	Algeria	20 542 087	19 771 728
2017	Angola	14 616 987	14 987 468
2017	Benin	5 684 202	5 680 642
2017	Botswana	1 159 836	1 193 966
2017	Burkina Faso	9 673 242	9 765 900
2017	Burundi	5 423 703	5 508 779
2017	Cabo Verde	275 363	278 292
2017	Cameroon	11 914 681	11 990 637
2017	Central African Republic	2 451 115	2 445 691
2017	Chad	7 419 320	7 364 846
2017	Comoros	374 246	372 185
2017	Congo	2 598 799	2 607 293
2017	Côte d’Ivoire	12 320 934	12 030 120
2017	Democratic Republic of the Congo	40 963 542	41 634 065
2017	Equatorial Guinea	751 027	670 653
2017	Eritrea	1 640 092	1 688 903
2017	Eswatini	557 277	571 085
2017	Ethiopia	53 330 472	52 703 519
2017	Gabon	1 071 533	1 025 878
2017	Gambia	1 159 309	1 174 250
2017	Ghana	14 772 110	14 845 706
2017	Guinea	5 903 977	6 091 997
2017	Guinea-Bissau	907 506	934 724
2017	Kenya	23 810 209	24 158 964
2017	Lesotho	1 048 564	1 078 642
2017	Liberia	2 335 626	2 365 072
2017	Madagascar	12 862 394	12 783 756
2017	Malawi	8 509 517	9 014 027
2017	Mali	9 559 386	9 365 741
2017	Mauritania	1 992 616	2 084 198
2017	Mauritius	613 188	626 401
2017	Mozambique	13 722 390	14 275 663
2017	Namibia	1 119 417	1 197 827
2017	Niger	10 801 360	10 501 803
2017	Nigeria	95 709 696	93 916 293
2017	Rwanda	5 841 328	6 144 404
2017	Sao Tome and Principe	101 788	102 089
2017	Senegal	7 294 364	7 560 273
2017	Seychelles	49 261	44 665
2017	Sierra Leone	3 767 379	3 756 636
2017	South Africa	26 915 964	28 592 421
2017	South Sudan	5 164 192	5 280 868
2017	Togo	3 864 970	3 830 769
2017	Uganda	19 426 892	19 897 651

2017	United Republic of Tanzania	27 207 648	27 934 043
2017	Zambia	8 355 415	8 596 678
2017	Zimbabwe	6 801 818	7 654 262
2018	Algeria	20 935 351	20 153 116
2018	Angola	15 136 584	15 511 479
2018	Benin	5 855 240	5 846 630
2018	Botswana	1 184 504	1 217 875
2018	Burkina Faso	9 947 963	10 036 905
2018	Burundi	5 589 539	5 674 064
2018	Cabo Verde	278 403	281 374
2018	Cameroon	12 251 209	12 324 003
2018	Central African Republic	2 498 271	2 494 614
2018	Chad	7 675 261	7 616 865
2018	Comoros	381 707	379 081
2018	Congo	2 662 256	2 669 985
2018	Côte d’Ivoire	12 635 485	12 348 624
2018	Democratic Republic of the Congo	42 333 104	43 012 504
2018	Equatorial Guinea	778 422	693 628
2018	Eritrea	1 664 004	1 712 464
2018	Eswatini	562 720	574 499
2018	Ethiopia	54 765 316	54 141 533
2018	Gabon	1 096 785	1 051 387
2018	Gambia	1 190 813	1 205 205
2018	Ghana	15 090 066	15 163 161
2018	Guinea	6 060 956	6 242 812
2018	Guinea-Bissau	929 815	956 640
2018	Kenya	24 289 086	24 665 152
2018	Lesotho	1 062 231	1 091 827
2018	Liberia	2 381 972	2 409 631
2018	Madagascar	13 193 278	13 116 332
2018	Malawi	8 744 222	9 256 303
2018	Mali	9 867 107	9 668 505
2018	Mauritania	2 046 830	2 138 468
2018	Mauritius	613 189	627 077
2018	Mozambique	14 138 348	14 697 053
2018	Namibia	1 138 622	1 218 946
2018	Niger	11 219 948	10 905 568
2018	Nigeria	98 161 817	96 258 054
2018	Rwanda	5 990 563	6 290 609
2018	Sao Tome and Principe	103 397	103 720
2018	Senegal	7 496 759	7 766 652
2018	Seychelles	49 812	45 015
2018	Sierra Leone	3 858 204	3 845 851
2018	South Africa	27 265 902	28 926 940
2018	South Sudan	5 036 439	5 150 984
2018	Togo	3 961 543	3 924 202
2018	Uganda	20 110 686	20 574 401

2018	United Republic of Tanzania	28 105 745	28 822 889
2018	Zambia	8 618 822	8 860 353
2018	Zimbabwe	6 944 282	7 806 857
2019	Algeria	21 321 765	20 529 496
2019	Angola	15 663 374	16 043 142
2019	Benin	6 029 083	6 015 553
2019	Botswana	1 208 538	1 241 169
2019	Burkina Faso	10 222 637	10 309 970
2019	Burundi	5 776 392	5 860 949
2019	Cabo Verde	281 235	284 255
2019	Cameroon	12 598 379	12 668 315
2019	Central African Republic	2 553 735	2 551 403
2019	Chad	7 932 926	7 871 403
2019	Comoros	389 183	385 983
2019	Congo	2 726 118	2 733 199
2019	Côte d’Ivoire	12 953 765	12 670 836
2019	Democratic Republic of the Congo	43 708 404	44 400 348
2019	Equatorial Guinea	805 443	716 526
2019	Eritrea	1 690 363	1 738 479
2019	Eswatini	568 205	578 016
2019	Ethiopia	56 227 924	55 610 258
2019	Gabon	1 121 437	1 076 493
2019	Gambia	1 222 395	1 236 310
2019	Ghana	15 409 204	15 482 640
2019	Guinea	6 222 991	6 396 996
2019	Guinea-Bissau	952 310	978 738
2019	Kenya	24 766 749	25 165 672
2019	Lesotho	1 075 997	1 105 191
2019	Liberia	2 429 853	2 455 730
2019	Madagascar	13 528 447	13 454 024
2019	Malawi	8 984 975	9 505 015
2019	Mali	10 179 829	9 976 246
2019	Mauritania	2 102 227	2 193 945
2019	Mauritius	613 044	627 621
2019	Mozambique	14 554 751	15 125 132
2019	Namibia	1 157 750	1 239 961
2019	Niger	11 651 966	11 322 559
2019	Nigeria	100 627 222	98 611 179
2019	Rwanda	6 140 770	6 437 557
2019	Sao Tome and Principe	104 981	105 326
2019	Senegal	7 703 406	7 977 360
2019	Seychelles	50 341	45 331
2019	Sierra Leone	3 949 845	3 936 047
2019	South Africa	27 643 789	29 281 525
2019	South Sudan	5 063 797	5 174 915
2019	Togo	4 059 175	4 019 058
2019	Uganda	20 816 774	21 273 326

2019	United Republic of Tanzania	28 972 170	29 702 958
2019	Zambia	8 885 069	9 127 797
2019	Zimbabwe	7 087 349	7 960 166
2020	Algeria	21 690 241	20 892 392
2020	Angola	16 187 147	16 572 769
2020	Benin	6 204 160	6 186 100
2020	Botswana	1 231 574	1 263 900
2020	Burkina Faso	10 504 230	10 587 944
2020	Burundi	5 945 267	6 030 554
2020	Cabo Verde	283 950	287 037
2020	Cameroon	12 946 413	13 014 851
2020	Central African Republic	2 618 638	2 617 521
2020	Chad	8 187 934	8 123 873
2020	Comoros	396 805	393 238
2020	Congo	2 790 601	2 797 530
2020	Côte d’Ivoire	13 276 669	12 998 886
2020	Democratic Republic of the Congo	45 144 658	45 851 443
2020	Equatorial Guinea	827 270	736 858
2020	Eritrea	1 718 443	1 766 308
2020	Eswatini	574 198	582 844
2020	Ethiopia	57 728 419	57 118 674
2020	Gabon	1 145 446	1 101 276
2020	Gambia	1 254 432	1 268 083
2020	Ghana	15 730 289	15 806 504
2020	Guinea	6 387 673	6 553 376
2020	Guinea-Bissau	974 714	1 000 798
2020	Kenya	25 265 084	25 680 980
2020	Lesotho	1 089 941	1 119 077
2020	Liberia	2 480 825	2 505 008
2020	Madagascar	13 865 456	13 795 217
2020	Malawi	9 230 383	9 759 137
2020	Mali	10 503 972	10 295 587
2020	Mauritania	2 158 370	2 250 262
2020	Mauritius	612 658	628 035
2020	Mozambique	14 987 433	15 567 241
2020	Namibia	1 177 627	1 261 689
2020	Niger	12 095 523	11 751 444
2020	Nigeria	103 138 311	101 022 546
2020	Rwanda	6 294 682	6 588 753
2020	Sao Tome and Principe	106 930	107 337
2020	Senegal	7 915 387	8 192 010
2020	Seychelles	50 827	45 666
2020	Sierra Leone	4 042 273	4 027 019
2020	South Africa	28 009 168	29 616 720
2020	South Sudan	5 143 111	5 250 992
2020	Togo	4 158 202	4 115 527
2020	Uganda	21 533 628	21 982 890

2020	United Republic of Tanzania	29 865 945	30 604 483
2020	Zambia	9 151 841	9 397 320
2020	Zimbabwe	7 237 516	8 118 758
2021	Algeria	22 047 299	21 247 111
2021	Angola	16 710 461	17 103 237
2021	Benin	6 379 517	6 357 440
2021	Botswana	1 252 180	1 284 474
2021	Burkina Faso	10 790 484	10 868 186
2021	Burundi	6 107 150	6 193 038
2021	Cabo Verde	286 497	289 668
2021	Cameroon	13 292 946	13 361 709
2021	Central African Republic	2 673 714	2 674 298
2021	Chad	8 451 059	8 385 086
2021	Comoros	404 449	400 745
2021	Congo	2 856 145	2 862 945
2021	Côte d’Ivoire	13 600 160	13 328 524
2021	Democratic Republic of the Congo	46 623 354	47 352 883
2021	Equatorial Guinea	846 108	755 669
2021	Eritrea	1 750 123	1 797 783
2021	Eswatini	579 994	588 432
2021	Ethiopia	59 234 536	58 642 830
2021	Gabon	1 168 563	1 125 792
2021	Gambia	1 286 857	1 300 260
2021	Ghana	16 048 025	16 128 346
2021	Guinea	6 552 182	6 709 086
2021	Guinea-Bissau	996 872	1 022 635
2021	Kenya	25 753 600	26 191 900
2021	Lesotho	1 103 184	1 132 641
2021	Liberia	2 533 470	2 556 078
2021	Madagascar	14 201 116	14 136 223
2021	Malawi	9 477 384	10 014 563
2021	Mali	10 839 529	10 627 354
2021	Mauritania	2 215 378	2 307 297
2021	Mauritius	612 372	628 636
2021	Mozambique	15 422 480	16 013 052
2021	Namibia	1 196 743	1 282 805
2021	Niger	12 552 952	12 194 715
2021	Nigeria	105 670 472	103 462 824
2021	Rwanda	6 450 594	6 742 056
2021	Sao Tome and Principe	109 079	109 566
2021	Senegal	8 131 116	8 408 071
2021	Seychelles	51 253	46 018
2021	Sierra Leone	4 134 464	4 117 765
2021	South Africa	28 316 716	29 887 693
2021	South Sudan	5 214 172	5 319 134
2021	Togo	4 258 372	4 213 560
2021	Uganda	22 246 625	22 690 077

2021	United Republic of Tanzania	30 789 300	31 527 267
2021	Zambia	9 416 824	9 666 839
2021	Zimbabwe	7 392 816	8 280 837
2022	Algeria	22 404 992	21 600 167
2022	Angola	17 238 952	17 638 256
2022	Benin	6 555 509	6 530 298
2022	Botswana	1 272 831	1 304 859
2022	Burkina Faso	11 073 172	11 147 115
2022	Burundi	6 272 907	6 358 877
2022	Cabo Verde	289 013	292 274
2022	Cameroon	13 644 072	13 712 174
2022	Central African Republic	2 732 585	2 734 975
2022	Chad	8 718 082	8 650 767
2022	Comoros	411 950	408 088
2022	Congo	2 922 164	2 928 852
2022	Côte d’Ivoire	13 931 259	13 666 073
2022	Democratic Republic of the Congo	48 135 674	48 894 333
2022	Equatorial Guinea	866 247	775 162
2022	Eritrea	1 781 520	1 828 831
2022	Eswatini	584 244	593 394
2022	Ethiopia	60 740 572	60 171 754
2022	Gabon	1 191 195	1 150 018
2022	Gambia	1 319 408	1 332 464
2022	Ghana	16 361 212	16 445 141
2022	Guinea	6 716 940	6 865 214
2022	Guinea-Bissau	1 019 063	1 044 392
2022	Kenya	26 241 998	26 704 939
2022	Lesotho	1 114 746	1 144 962
2022	Liberia	2 587 888	2 608 739
2022	Madagascar	14 539 494	14 479 986
2022	Malawi	9 726 364	10 270 847
2022	Mali	11 178 683	10 963 036
2022	Mauritania	2 274 803	2 366 613
2022	Mauritius	610 083	627 189
2022	Mozambique	15 852 693	16 457 436
2022	Namibia	1 213 792	1 301 880
2022	Niger	13 027 515	12 656 302
2022	Nigeria	08 239 173	05 931 214
2022	Rwanda	6 606 218	6 894 945
2022	Sao Tome and Principe	111 139	111 693
2022	Senegal	8 345 722	8 624 398
2022	Seychelles	61 851	55 631
2022	Sierra Leone	4 225 948	4 207 655
2022	South Africa	28 584 646	30 111 361
2022	South Sudan	5 296 447	5 398 453
2022	Togo	4 359 261	4 312 465
2022	Uganda	22 933 305	23 371 288
2022	United Republic of Tanzania	31 723 512	32 464 281
2022	Zambia	9 680 089	9 937 232
2022	Zimbabwe	7 551 489	8 442 636

Supplemental Table 4. Distribution of contextual factors by sex according to hypertension status

	Overall population	Females: n=149 518 (50.5)		P-value[a]	Males: n=102 243 (49.5)		P-value[a]
		Hypertension	No hypertension		Hypertension	No hypertension	
Number of participants	251 761 (100.0)	43240 (25.9)	106287 (74.1)		29666 (24.3)	72577 (75.7)	
Year of survey (country/survey)				<.0001			<.0001
2003-2006 (11/11)	32009 (1.0)	5522 (2.0)	12881 (0.8)		4447 (1.7)	9159 (0.6)	
2007-2010 (18/19)	63013 (6.8)	10988 (8.2)	26026 (7.8)		8134 (7.6)	17865 (4.9)	
2011-2014 (13/15)	69560 (20.9)	11518 (21.5)	31598 (21.8)		7083 (21.1)	19361 (19.8)	
2015-2018 (8/9)	49636 (60.1)	8792 (56.2)	19719 (58.9)		5986 (57.3)	15139 (63.6)	
2019-2022 (7/7)	37543 (11.2)	6420 (12.1)	16054 (10.7)		4016 (12.3)	11053 (11.0)	
Subregion (country/survey)				<.0001			<.0001
North Africa (1/2)	10418 (14.8)	2132 (18)	12881 (0.8)		1291 (16.4)	3104 (14.6)	
Western Africa (12/22)	95516 (15.3)	14661 (16.4)	26026 (7.8)		11937 (17.3)	28428 (14.4)	
Middle Africa (7/9)	27460 (0.2)	5022 (0.4)	31598 (21.8)		3942 (0.2)	7802 (0.1)	
East Africa (12/19)	84065 (68.9)	13884 (64.0)	19719 (58.9)		8201 (65.0)	23865 (70.2)	
Southern Africa (5/9)	34302 (0.8)	7541 (1.2)	16054 (10.7)		4295 (1.0)	9378 (0.7)	
Word Bank country classification by income (country/survey)				<.0001			0.0001
Low income (25/41)	181647 (68.6)	28058 (64.1)	79437 (70.1)		20413 (65.7)	53739 (69.5)	
Lower middle-income (7/13)	42210 (16.2)	8578 (17.2)	16508 (16.2)		5317 (17.2)	11807 (15.5)	
Upper middle-income (5/7)	27904 (15.2)	6604 (18.7)	10333 (13.7)		3936 (17.1)	7031 (14.9)	
Place of residence (country/survey)				<.0001			0.0009
Urban (25/34)	82355 (32.9)	14267 (38.3)	34997 (32.5)		9970 (34.7)	23121 (31.0)	
Rural (20/26)	81203 (67.1)	12079 (61.7)	36122 (67.5)		8038 (65.3)	24964 (69.0)	

[a]P-value of Chi-Square Rao-Scott test. Survey weights were incorporated into the estimates of all percentages.

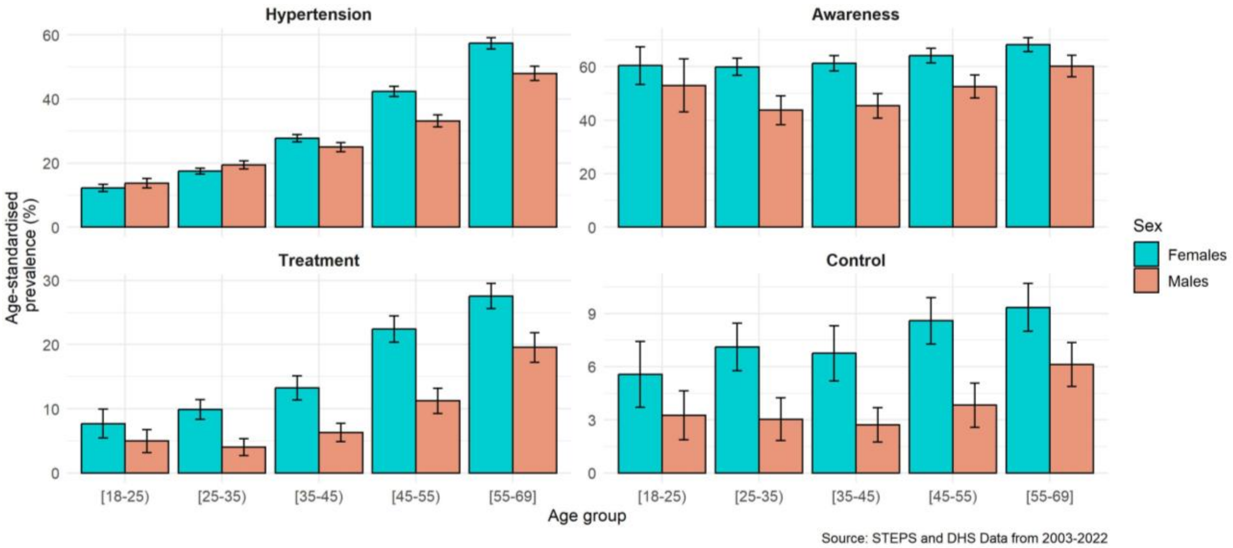

Supplemental Figure 1. Sex-stratified hypertension prevalence, awareness, treatment, and control rates by age group.

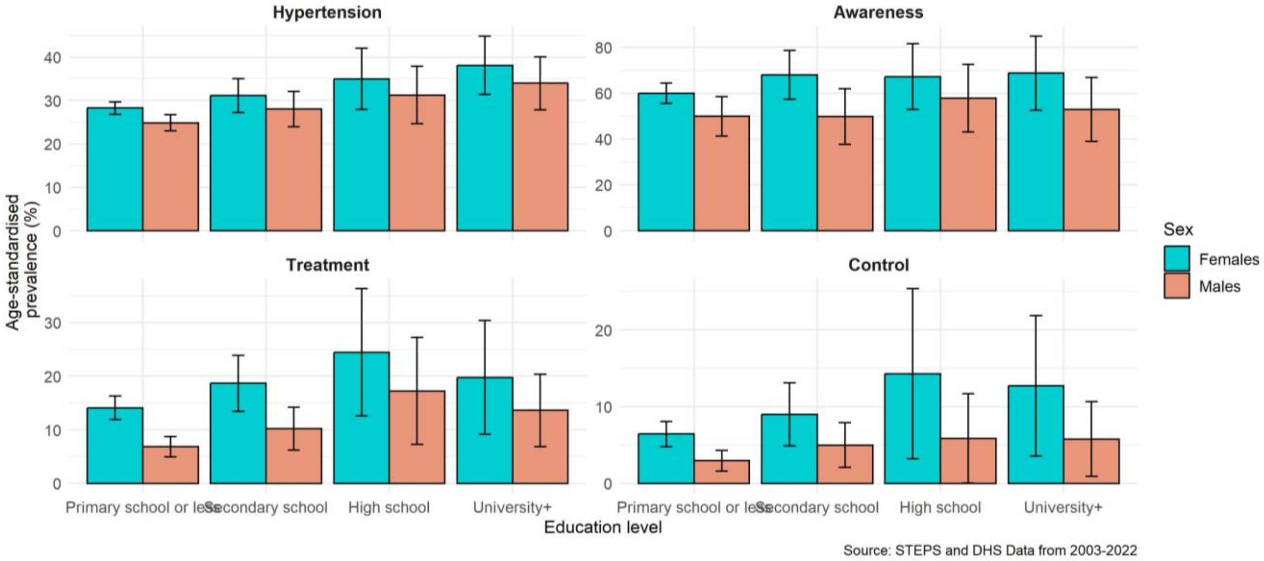

Supplemental Figure 2. Sex-stratified age-standardized hypertension prevalence, awareness, treatment, and control rates by education level.

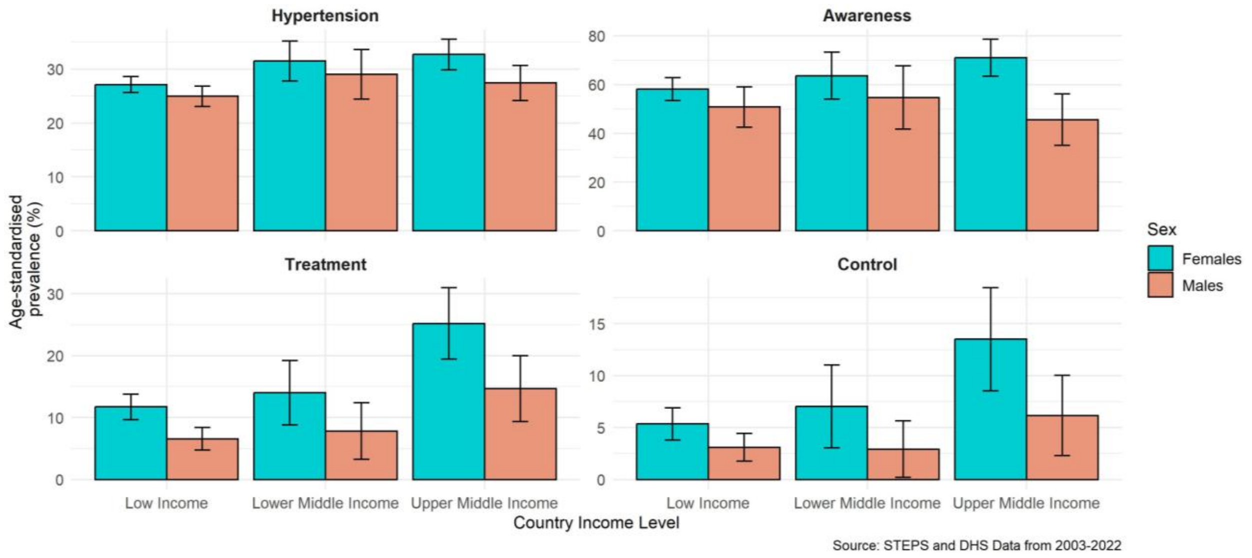

Supplemental Figure 3. Sex-stratified age-standardized hypertension prevalence, awareness, treatment, and control rates by country income level.

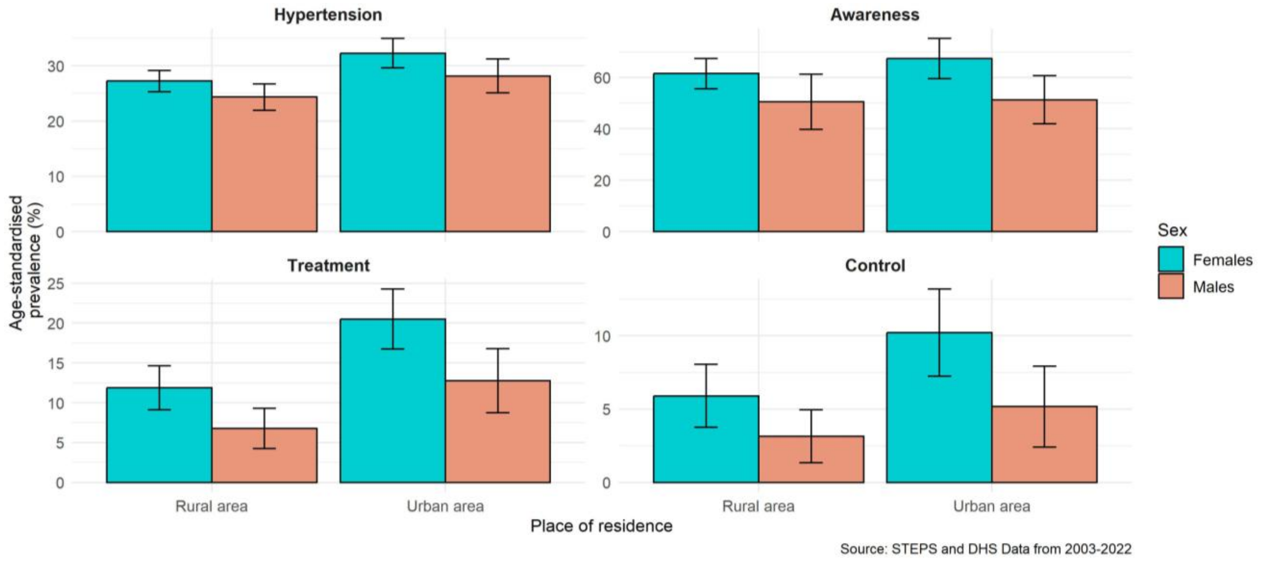

Supplemental Figure 4. Sex-stratified age-standardized hypertension prevalence, awareness, treatment, and control rates by rural/urban status.

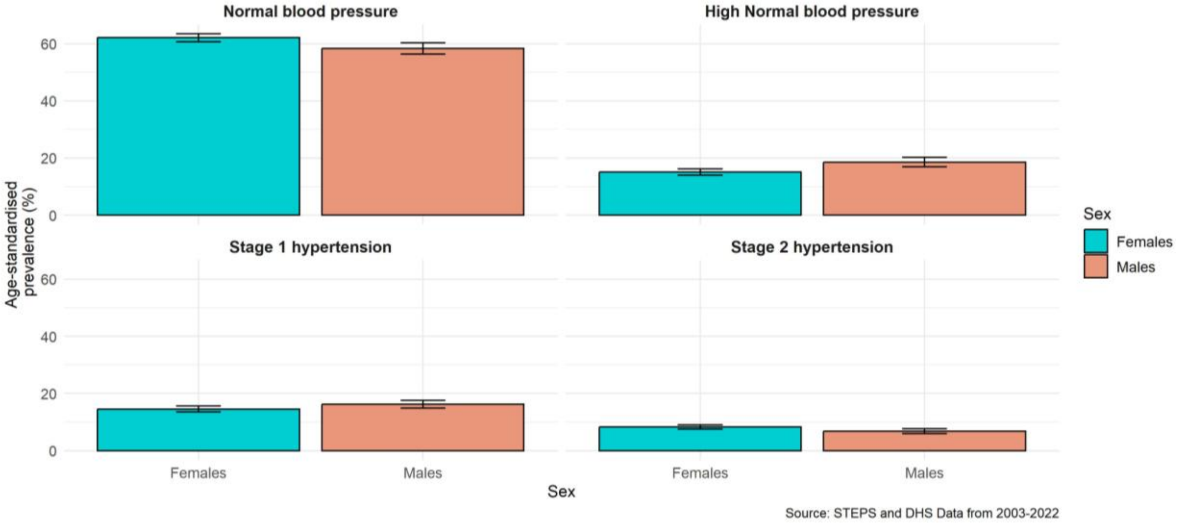

Supplemental Figure 5. Sex-stratified age-standardized prevalence of blood pressure categories.

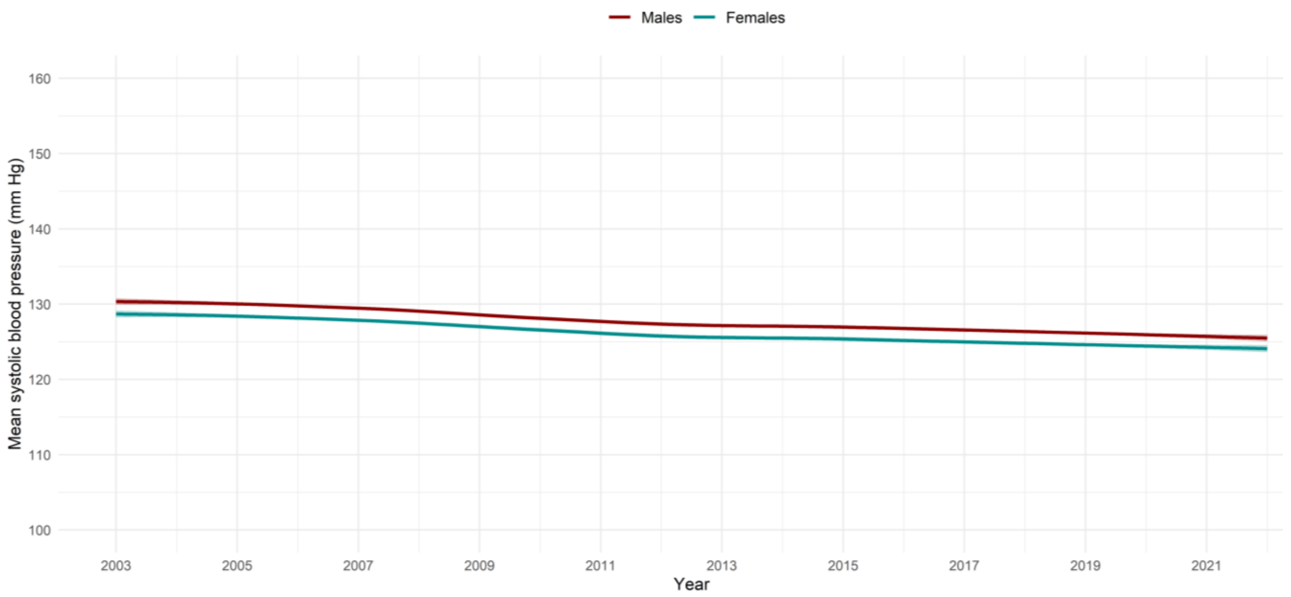

Supplemental Figure 6. Sex-stratified trend in age-standardized mean systolic blood pressure from 2003–2022.

Imputed data for all the 47 countries in the WHO African region. WHO = World Health Organization.

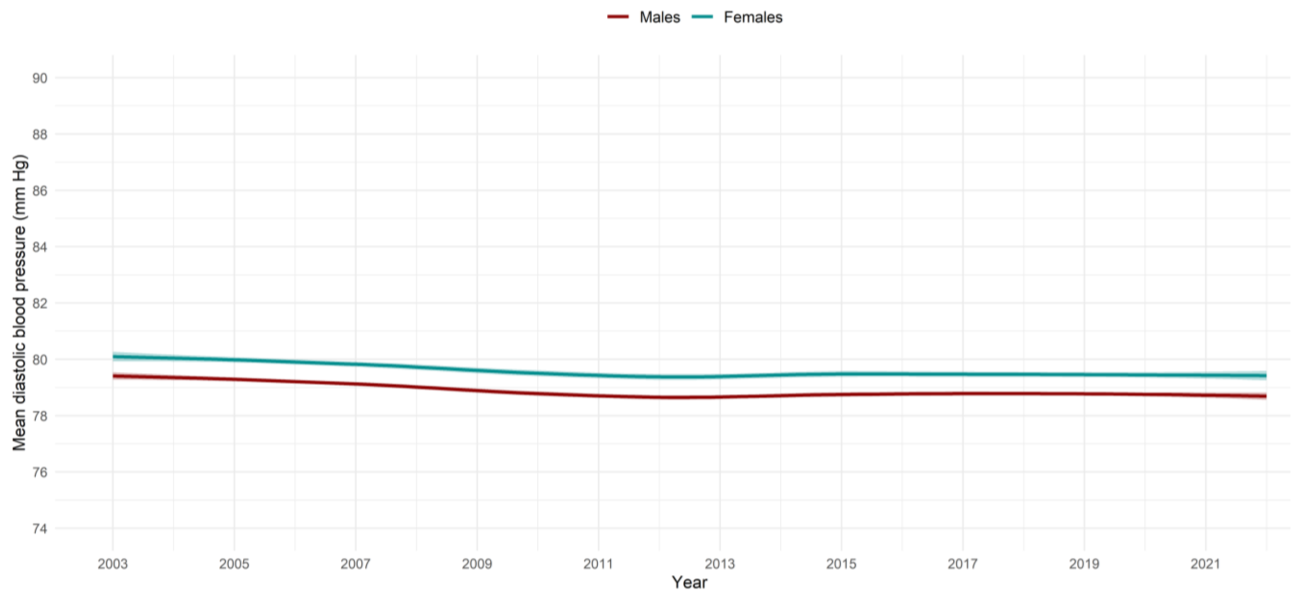

Supplemental Figure 7. Sex-stratified trend in age-standardized mean diastolic blood pressure from 2003–2022.

Imputed data for all the 47 countries in the WHO African region. WHO = World Health Organization.

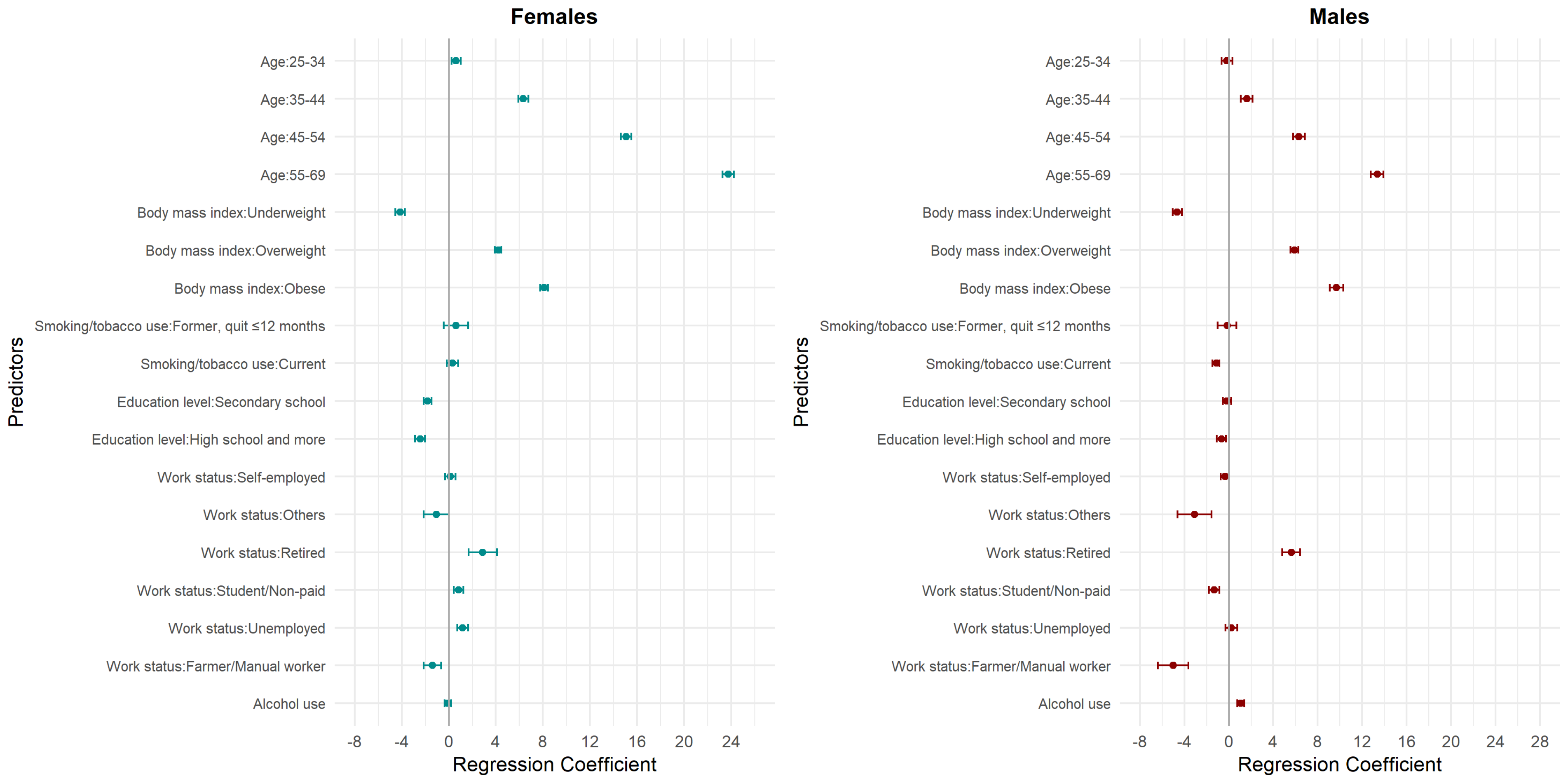

Supplemental Figure 8. Determinants of systolic blood pressure levels: sex-stratified multivariable hierarchical analysis.
Estimates were obtained by linear regression analyses with random effects for country and survey year. The analysis was conducted among all participants. Dots represent adjusted regression coefficients and horizontal lines their 95% confidence intervals for each predictor. A positive coefficient indicates a higher systolic blood pressure (i.e., a positive association) and a negative coefficient indicates a lower systolic blood pressure (i.e., a negative association).

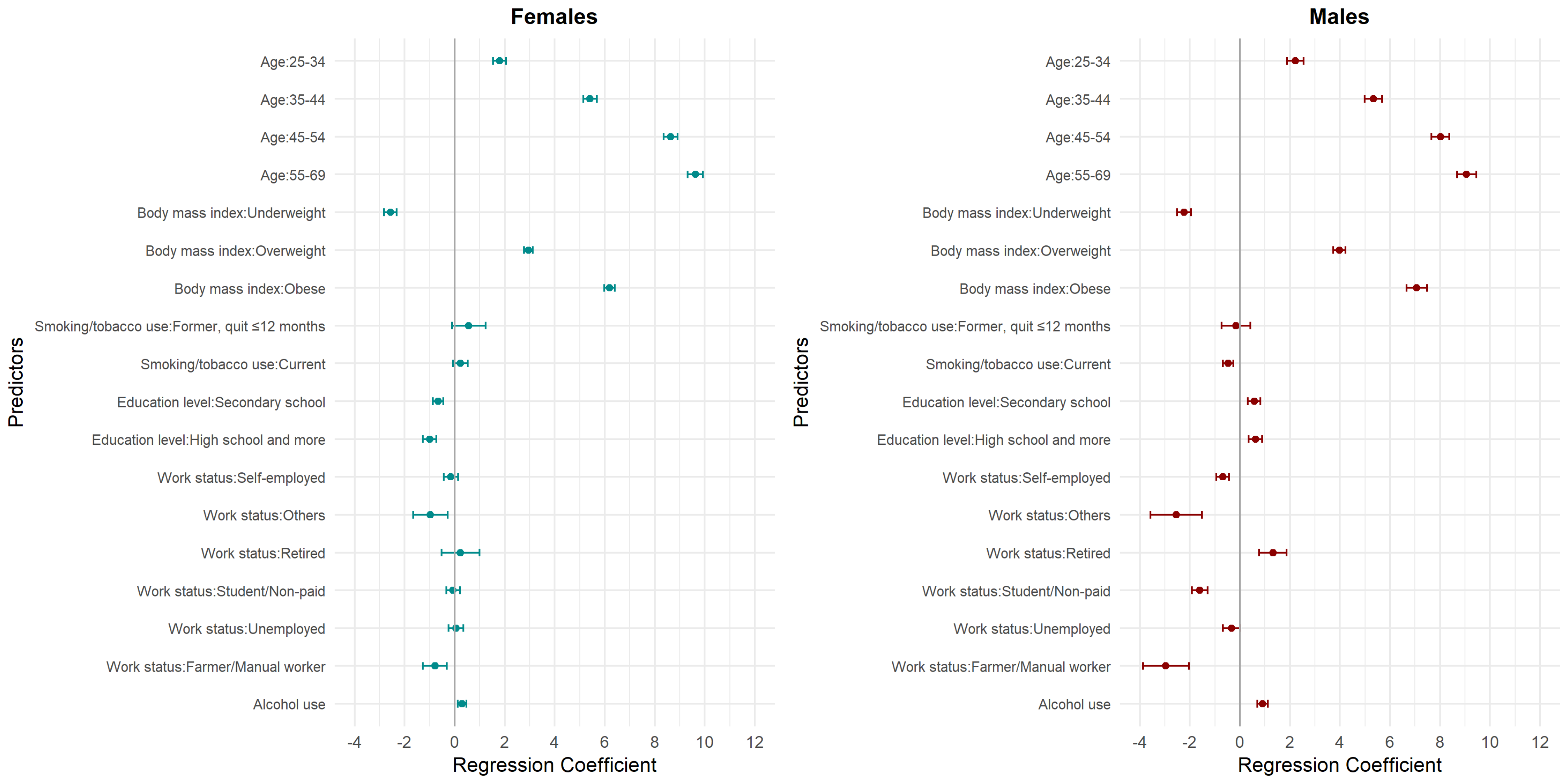

Supplemental Figure 9. Determinants of diastolic blood pressure levels: sex-stratified multivariable hierarchical analysis.
Estimates were obtained by linear regression analyses with random effects for country and survey year. The analysis was conducted among all participants. Dots represent adjusted regression coefficients and horizontal lines their 95% confidence intervals for each predictor. A positive coefficient indicates a higher diastolic blood pressure (i.e., a positive association) and a negative coefficient indicates a lower diastolic blood pressure (i.e., a negative association).

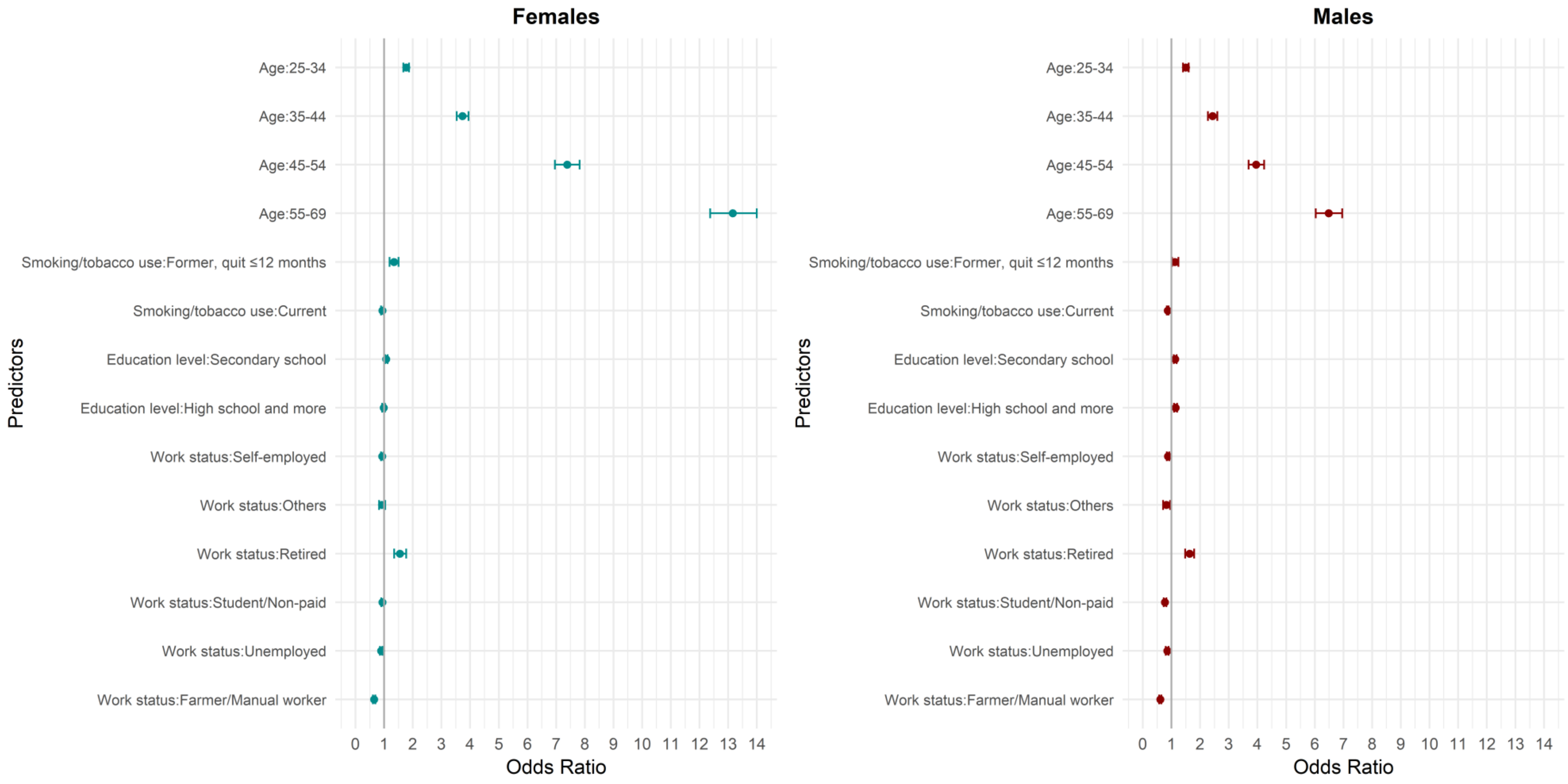

Supplemental Figure 10. Determinants of hypertension status: sex-stratified multivariable hierarchical analysis, adjusted for covariates commonly available in STEPS and DHS surveys (parsimonious model).

Odds ratios (ORs) and their 95% confidence intervals (CIs) were estimated via logistic regression analyses with random effects for country and survey year. The analysis was conducted among all participants. Dots represent adjusted ORs and horizontal lines their 95% CIs for each predictor. An OR greater than 1 indicates a positive association (increased odds of hypertension) and an OR less than 1 indicates a negative association (reduced odds).

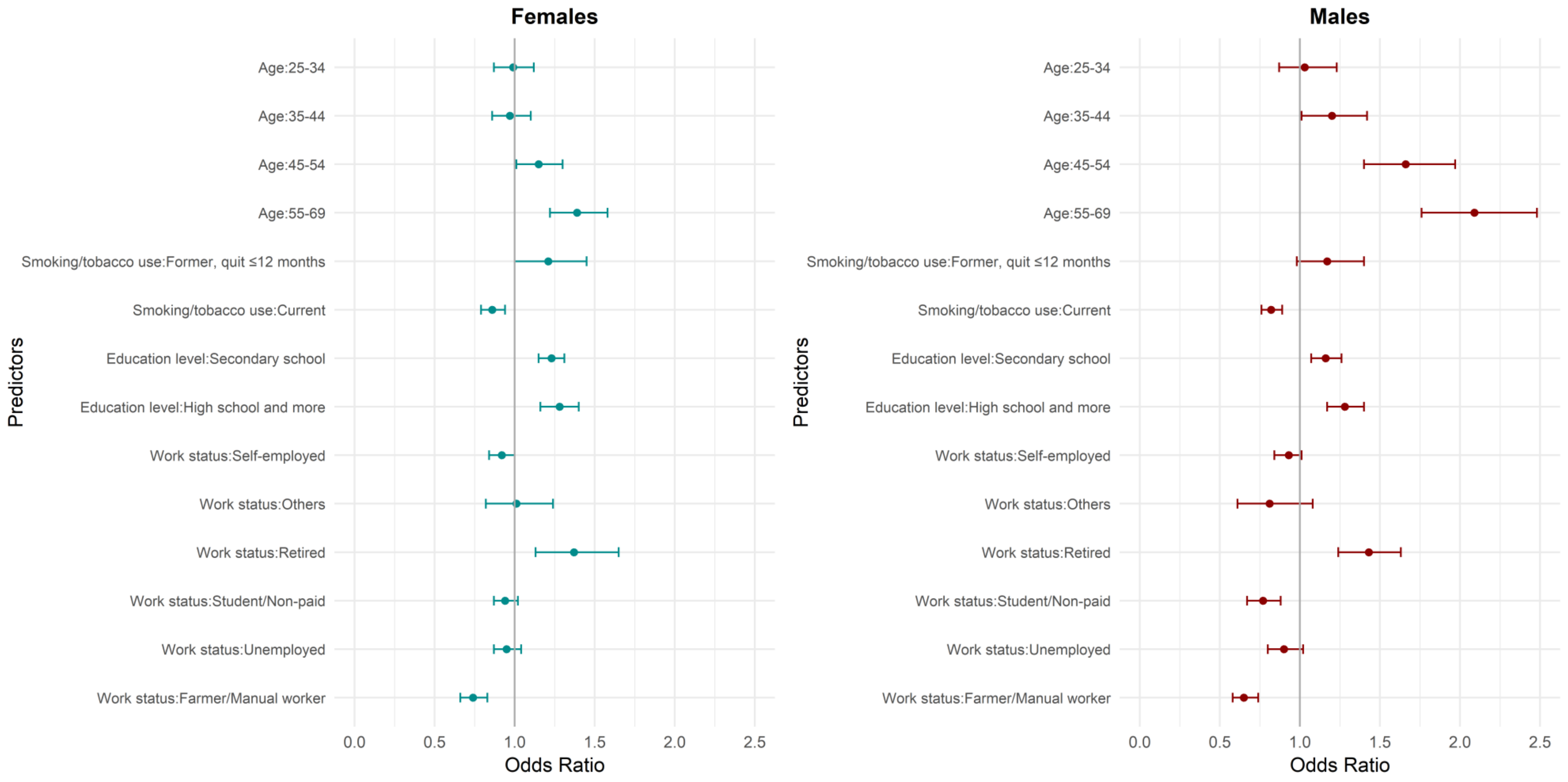

Supplemental Figure 11. Determinants of hypertension awareness: sex-stratified multivariable hierarchical analysis, adjusted for covariates commonly available in STEPS and DHS surveys (parsimonious model).

Odds ratios (ORs) and their 95% confidence intervals (CIs) were estimated via logistic regression analyses with random effects for country and survey year. The analysis was conducted among hypertensive individuals only. Dots represent adjusted ORs and horizontal lines their 95% CIs for each predictor. An OR greater than 1 indicates a positive association (increased odds of hypertension awareness) and an OR less than 1 indicates a negative association (reduced odds).

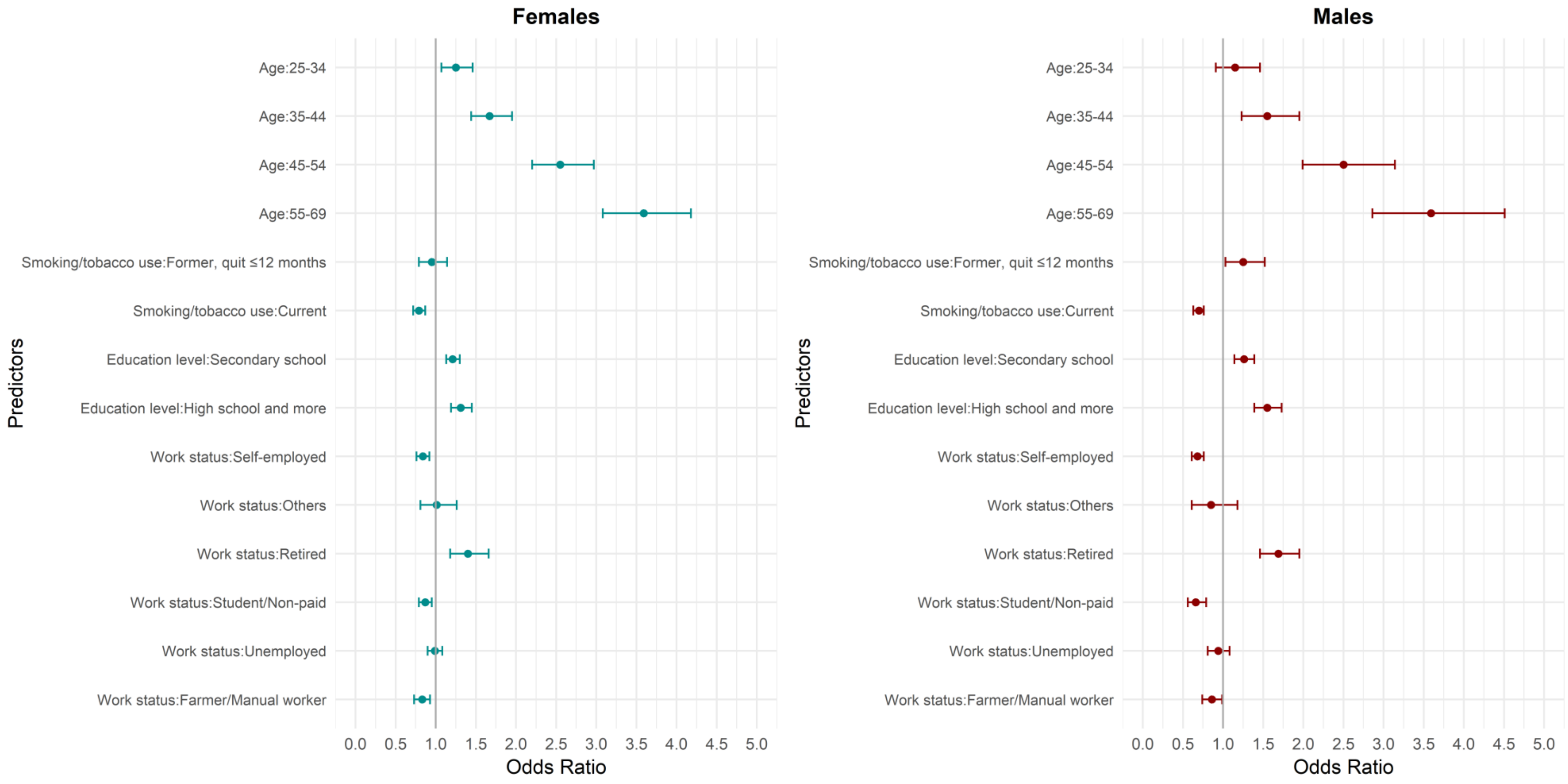

Supplemental Figure 12. Determinants of hypertension treatment: sex-stratified multivariable hierarchical analysis, adjusted for covariates commonly available in STEPS and DHS surveys (parsimonious model).

Odds ratios (ORs) and their 95% confidence intervals (CIs) were estimated via logistic regression analyses with random effects for country and survey year. The analysis was conducted among hypertensive individuals only. Dots represent adjusted ORs and horizontal lines their 95% CIs for each predictor. An OR greater than 1 indicates a positive association (increased odds of hypertension awareness) and an OR less than 1 indicates a negative association (reduced odds).

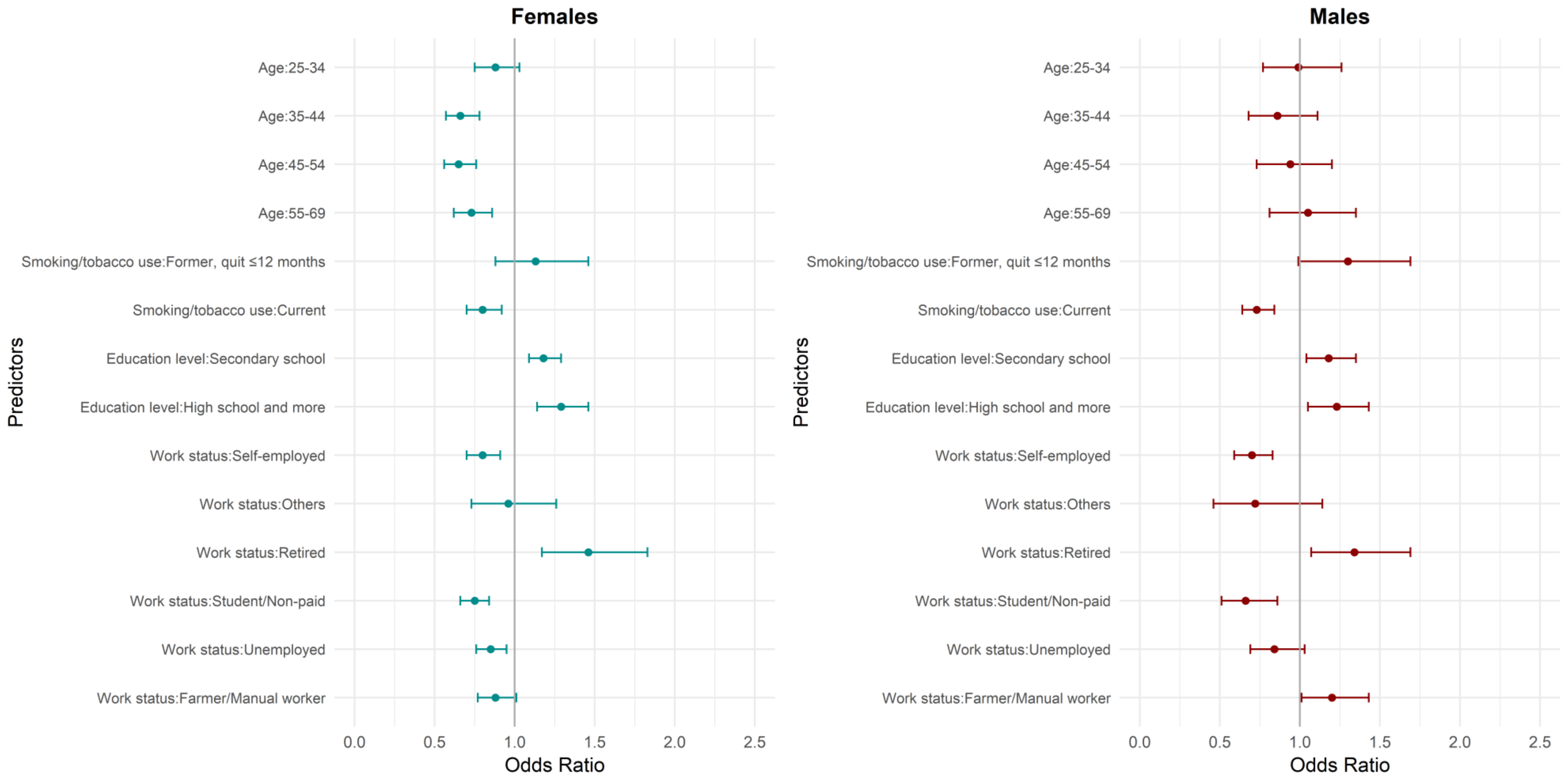

Supplemental Figure 13. Determinants of hypertension control: sex-stratified multivariable hierarchical analysis, adjusted for covariates commonly available in STEPS and DHS surveys (parsimonious model).

Odds ratios (ORs) and their 95% confidence intervals (CIs) were estimated via logistic regression analyses with random effects for country and survey year. The analysis was conducted among hypertensive individuals only. Dots represent adjusted ORs and horizontal lines their 95% CIs for each predictor. An OR greater than 1 indicates a positive association (increased odds of hypertension awareness) and an OR less than 1 indicates a negative association (reduced odds).

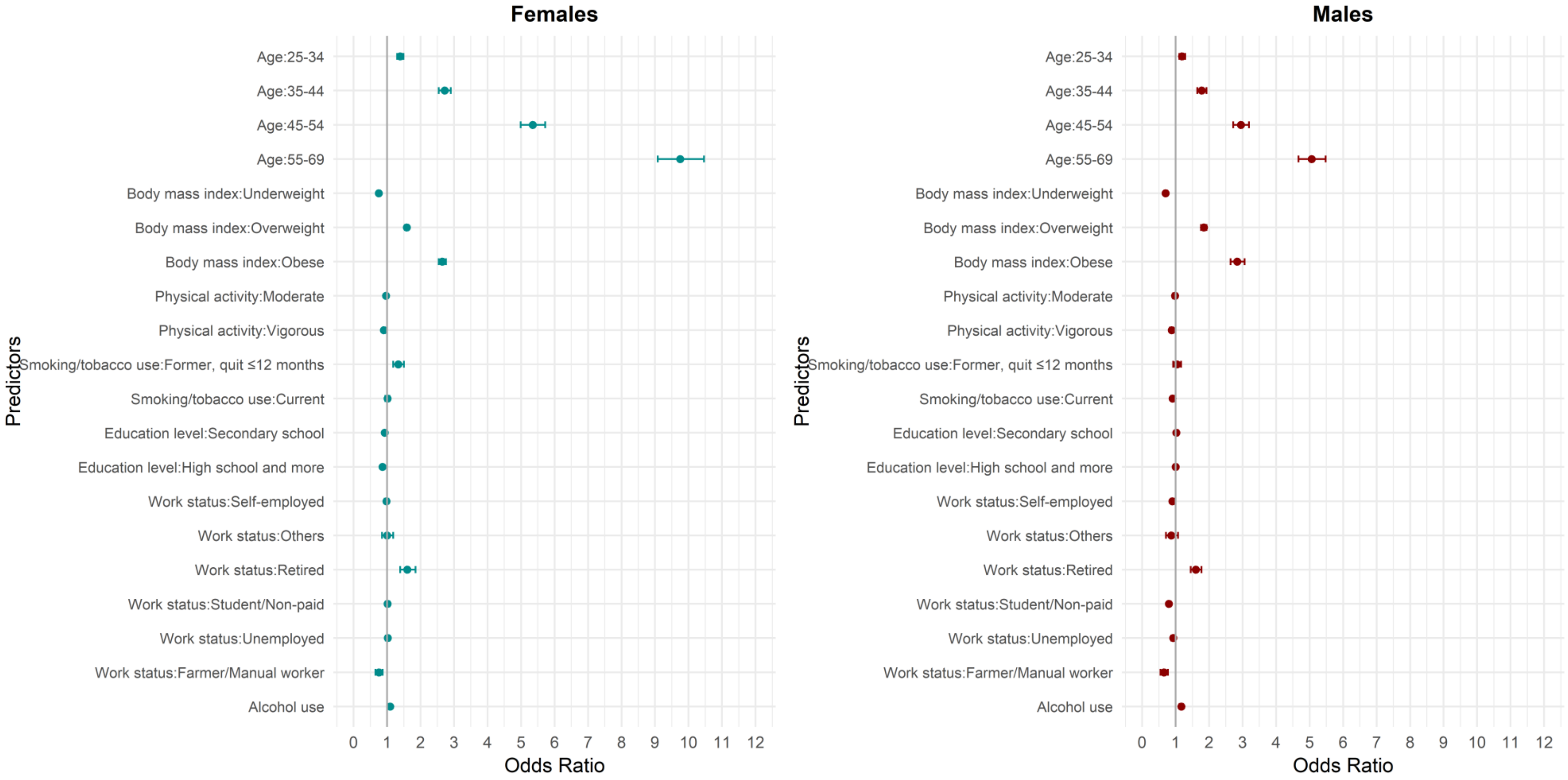

Supplemental Figure 14. Determinants of hypertension status: sex-stratified multivariable hierarchical analysis, adjusted for physical activity.
Odds ratios (ORs) and their 95% confidence intervals (CIs) were estimated via logistic regression analyses with random effects for country and survey year. The analysis was conducted among all participants. Dots represent adjusted ORs and horizontal lines their 95% confidence intervals for each predictor. An OR greater than 1 indicates a positive association (increased odds of hypertension) and an OR less than 1 indicates a negative association (reduced odds).

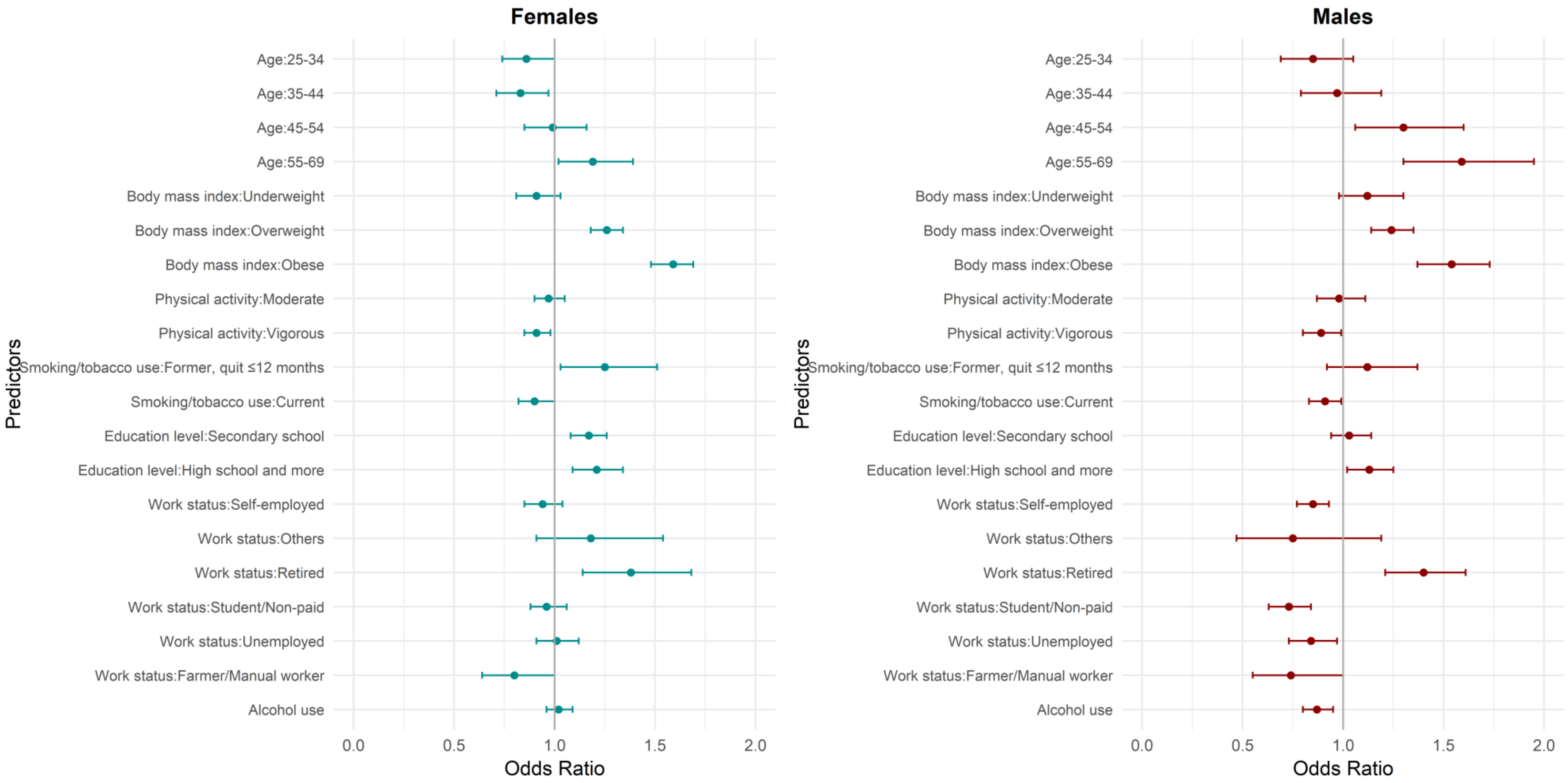

Supplemental Figure 15. Determinants of hypertension awareness: sex-stratified multivariable hierarchical analysis, adjusted for physical activity.
Odds ratios (ORs) and their 95% confidence intervals (CIs) were estimated via logistic regression analyses with random effects for country and survey year. The analysis was conducted among hypertensive individuals only. Dots represent adjusted ORs and horizontal lines their 95% confidence intervals for each predictor. An OR greater than 1 indicates a positive association (increased odds of hypertension awareness) and an OR less than 1 indicates a negative association (reduced odds).

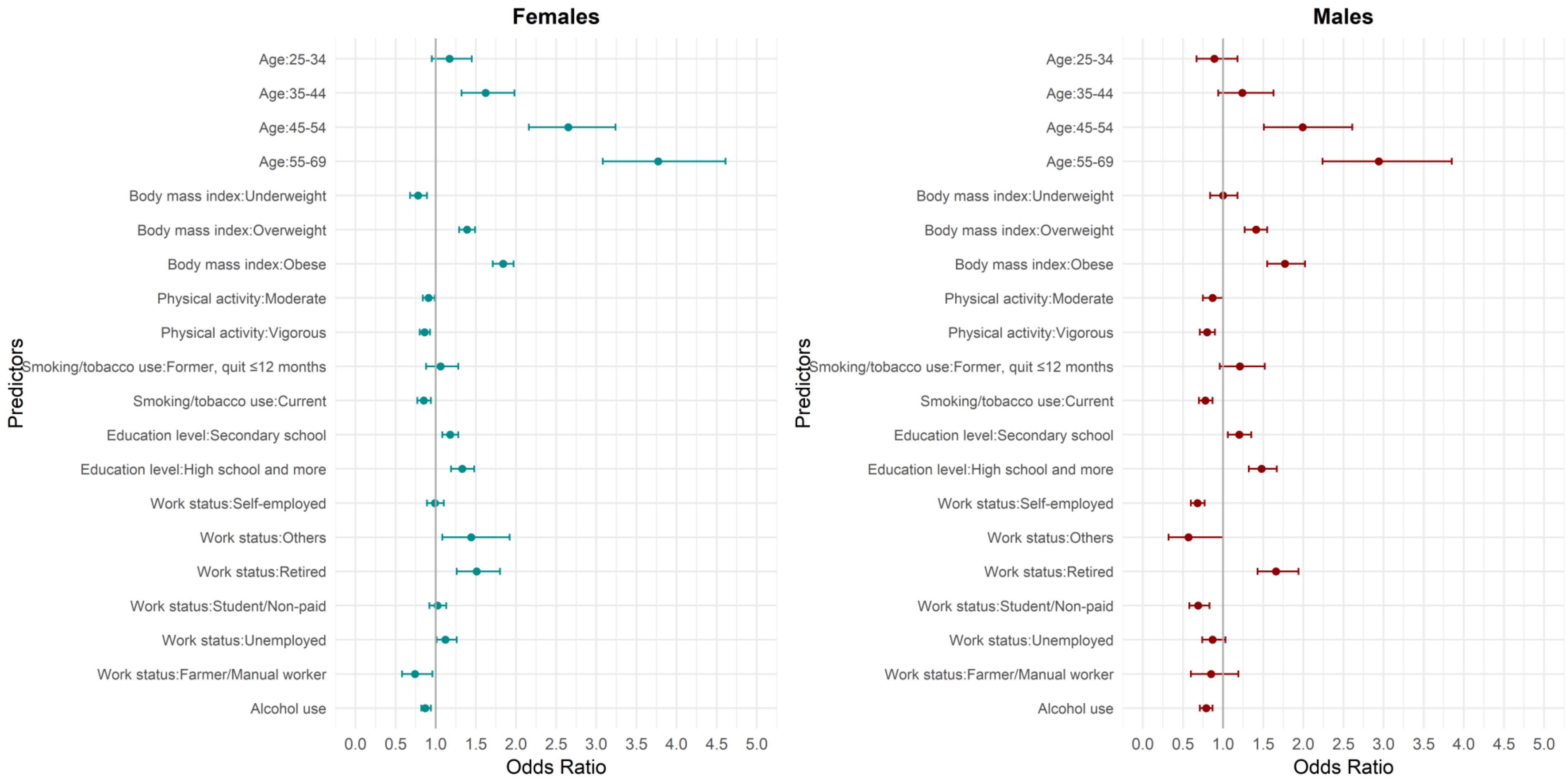

Supplemental Figure 16. Determinants of hypertension treatment: sex-stratified multivariable hierarchical analysis, adjusted for physical activity.
Odds ratios (ORs) and their 95% confidence intervals (CIs) were estimated via logistic regression analyses with random effects for country and survey year. The analysis was conducted among hypertensive individuals only. Dots represent adjusted ORs and horizontal lines their 95% confidence intervals for each predictor. An OR greater than 1 indicates a positive association (increased odds of hypertension treatment) and an OR less than 1 indicates a negative association (reduced odds).

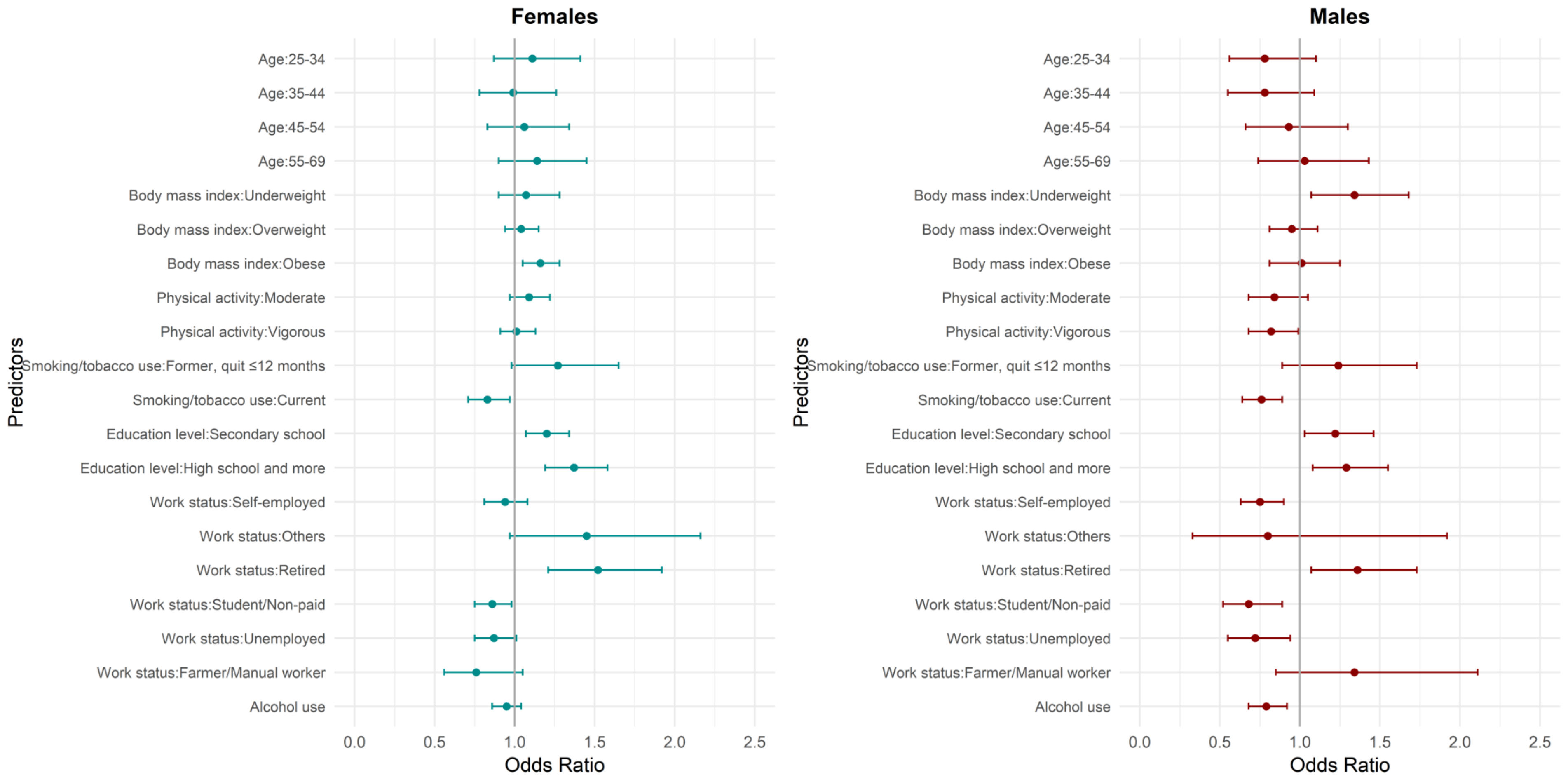

Supplemental Figure 17. Determinants of hypertension control: sex-stratified multivariable hierarchical analysis, adjusted for physical activity.
Odds ratios (ORs) and their 95% confidence intervals (CIs) were estimated via logistic regression analyses with random effects for country and survey year. The analysis was conducted among hypertensive individuals only. Dots represent adjusted ORs and horizontal lines their 95% confidence intervals for each predictor. An OR greater than 1 indicates a positive association (increased odds of hypertension control) and an OR less than 1 indicates a negative association (reduced odds).

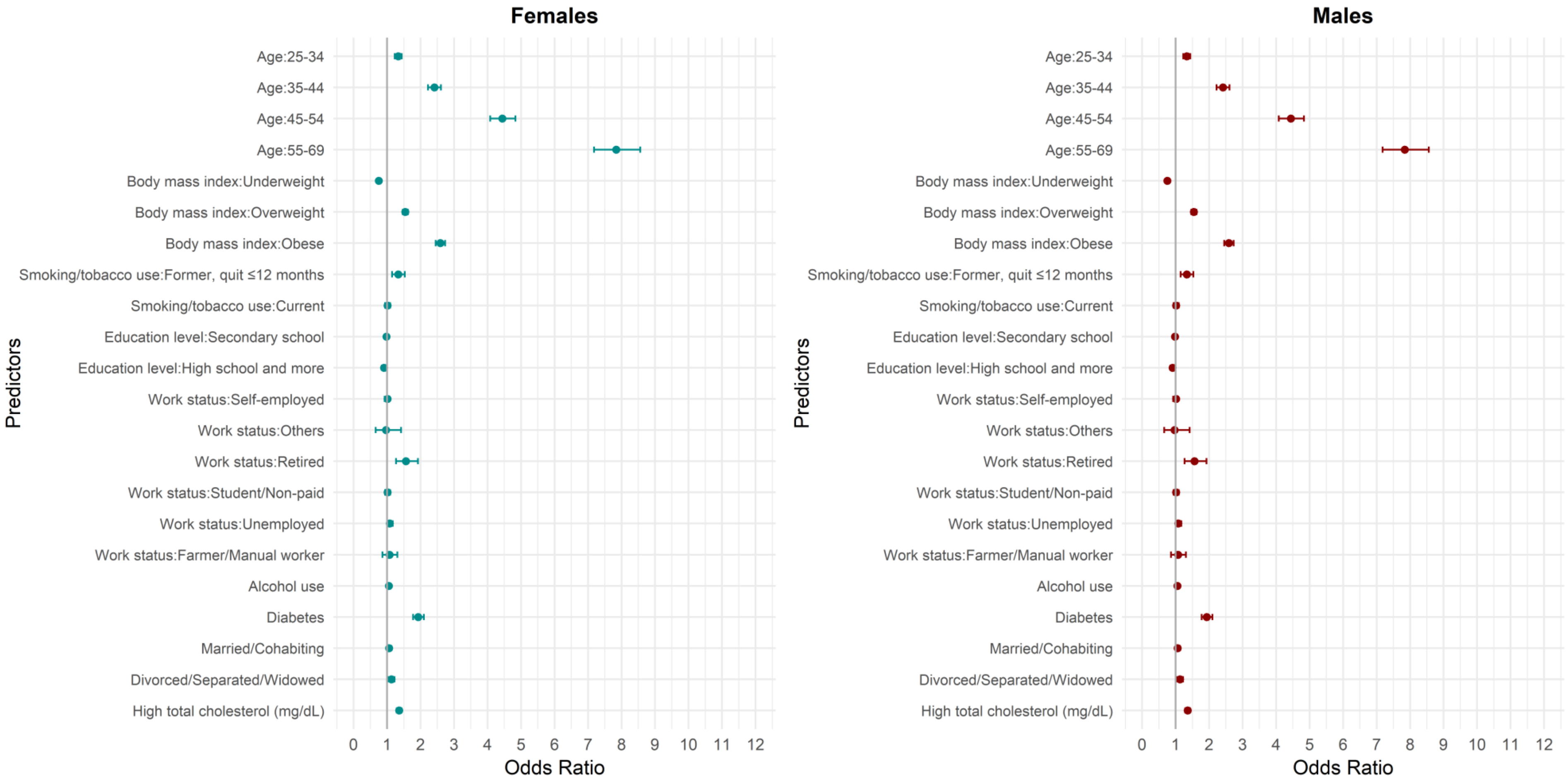

Supplemental Figure 18. Determinants of hypertension status: sex-stratified multivariable hierarchical analysis, adjusted for diabetes, marital status, and cholesterol levels. Odds ratios (ORs) and their 95% confidence intervals (CIs) were estimated via logistic regression analyses with random effects for country and survey year. The analysis was conducted among all participants. Dots represent adjusted ORs and horizontal lines their 95% confidence intervals for each predictor. An OR greater than 1 indicates a positive association (increased odds of hypertension) and an OR less than 1 indicates a negative association (reduced odds).

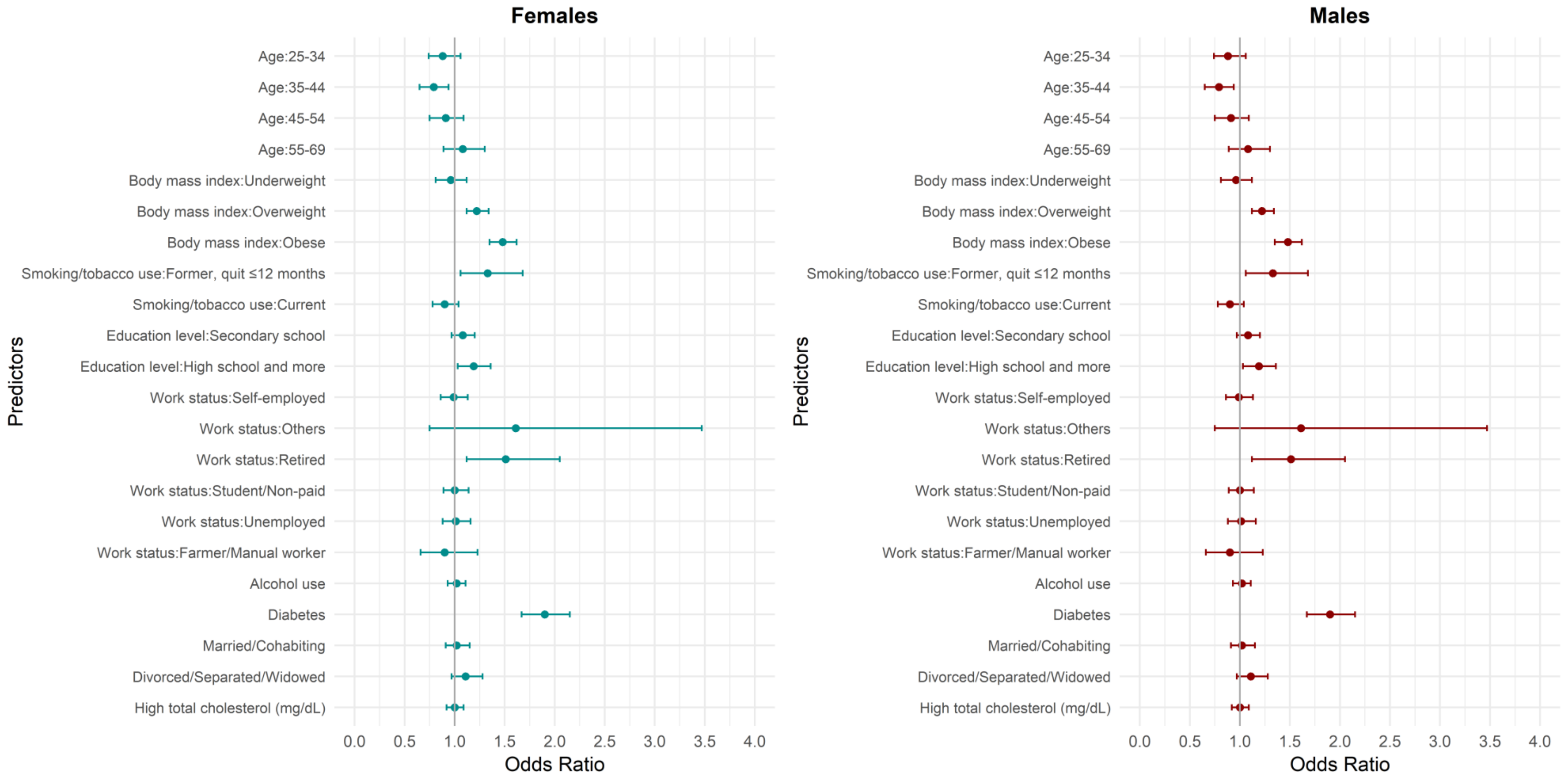

Supplemental Figure 19. Determinants of hypertension awareness: sex-stratified multivariable hierarchical analysis, adjusted for diabetes, marital status, and cholesterol levels.

Odds ratios (ORs) and their 95% confidence intervals (CIs) were estimated via logistic regression analyses with random effects for country and survey year. The analysis was conducted among hypertensive individuals only. Dots represent adjusted ORs and horizontal lines their 95% confidence intervals for each predictor. An OR greater than 1 indicates a positive association (increased odds of hypertension awareness) and an OR less than 1 indicates a negative association (reduced odds).

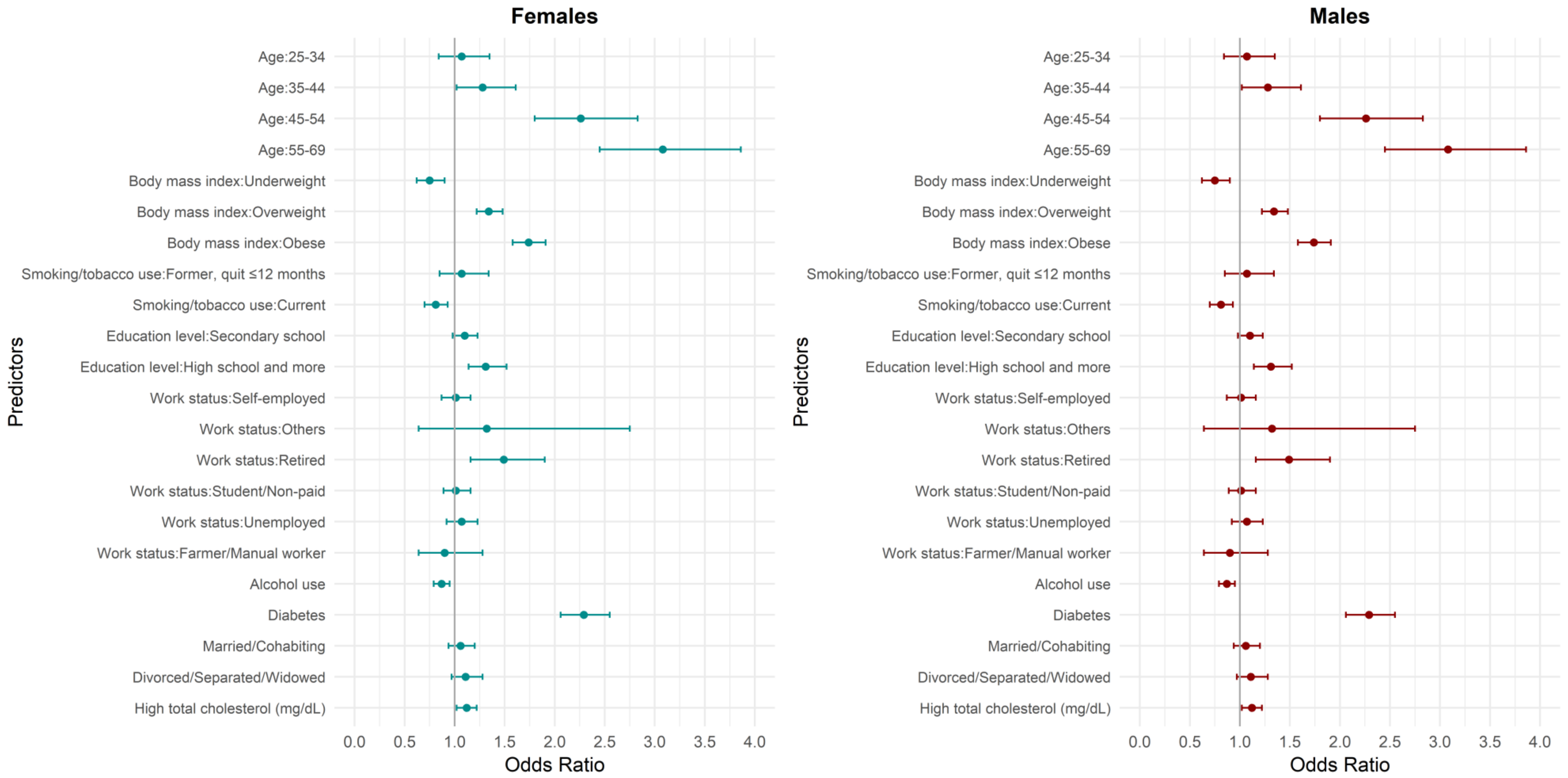

Supplemental Figure 20. Determinants of hypertension treatment: sex-stratified multivariable hierarchical analysis, adjusted for diabetes, marital status, and cholesterol levels.

Odds ratios (ORs) and their 95% confidence intervals (CIs) were estimated via logistic regression analyses with random effects for country and survey year. The analysis was conducted among hypertensive individuals only. Dots represent adjusted ORs and horizontal lines their 95% confidence intervals for each predictor. An OR greater than 1 indicates a positive association (increased odds of hypertension treatment) and an OR less than 1 indicates a negative association (reduced odds).

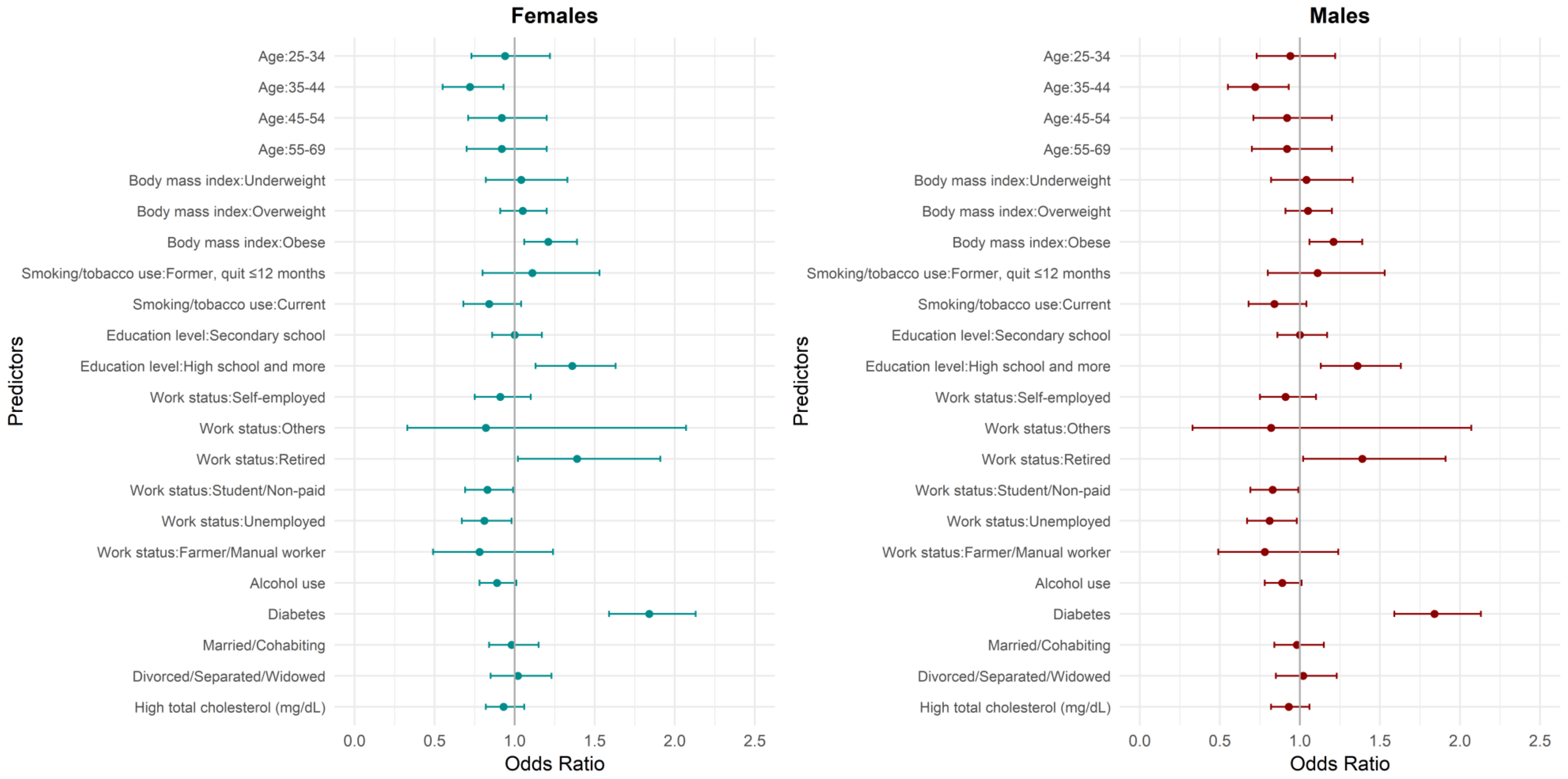

Supplemental Figure 21. Determinants of hypertension control: sex-stratified multivariable hierarchical analysis, adjusted for diabetes, marital status, and cholesterol levels.

Odds ratios (ORs) and their 95% confidence intervals (CIs) were estimated via logistic regression analyses with random effects for country and survey year. The analysis was conducted among hypertensive individuals only. Dots represent adjusted ORs and horizontal lines their 95% confidence intervals for each predictor. An OR greater than 1 indicates a positive association (increased odds of hypertension control) and an OR less than 1 indicates a negative association (reduced odds).

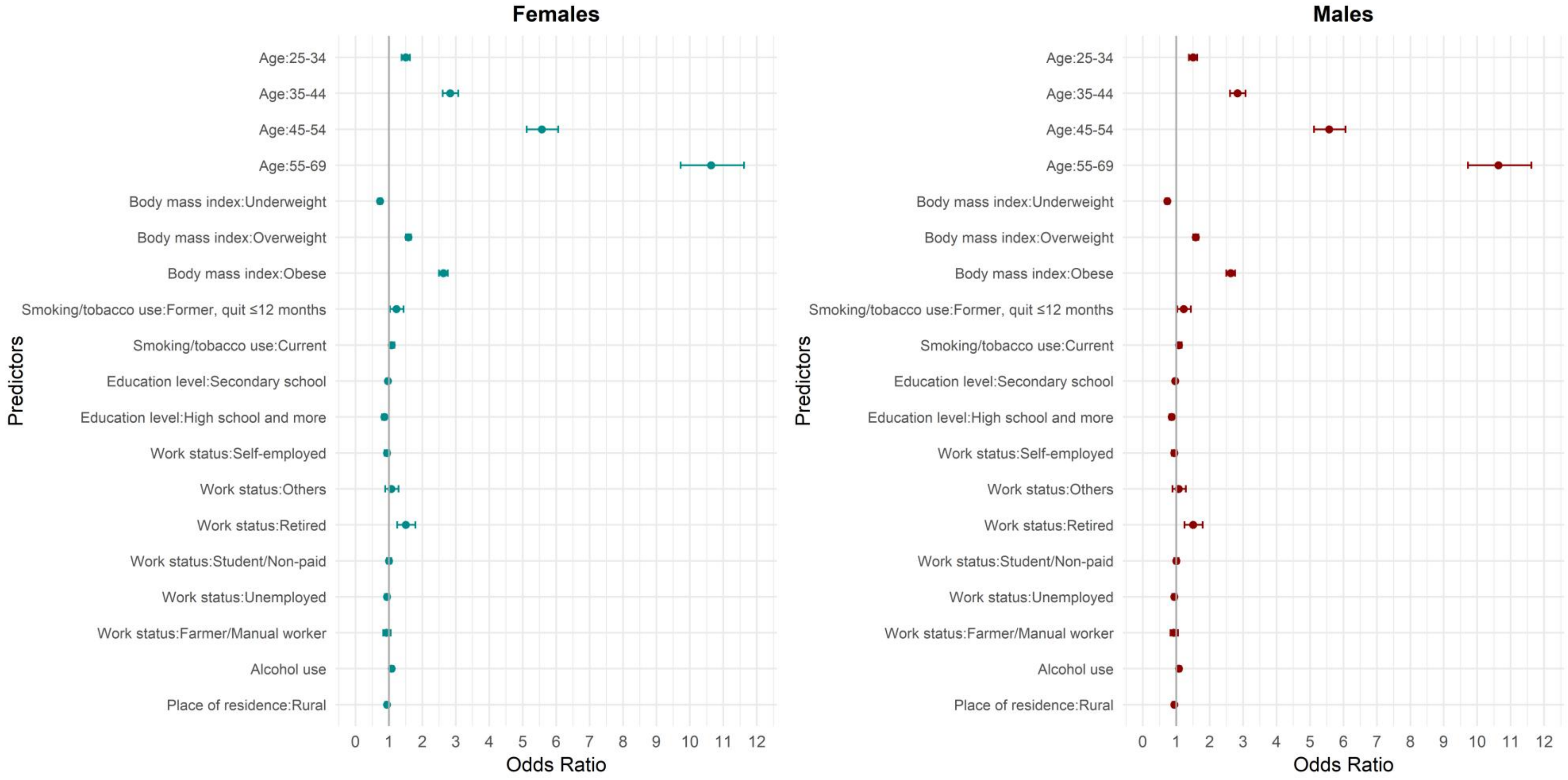

Supplemental Figure 22. Determinants of hypertension status: sex-stratified multivariable hierarchical analysis, adjusted for rural/urban status.
Odds ratios (ORs) and their 95% confidence intervals (CIs) were estimated via logistic regression analyses with random effects for country and survey year. The analysis was conducted among all participants. Dots represent adjusted ORs and horizontal lines their 95% CIs for each predictor. An OR greater than 1 indicates a positive association (increased odds of hypertension) and an OR less than 1 indicates a negative association (reduced odds).

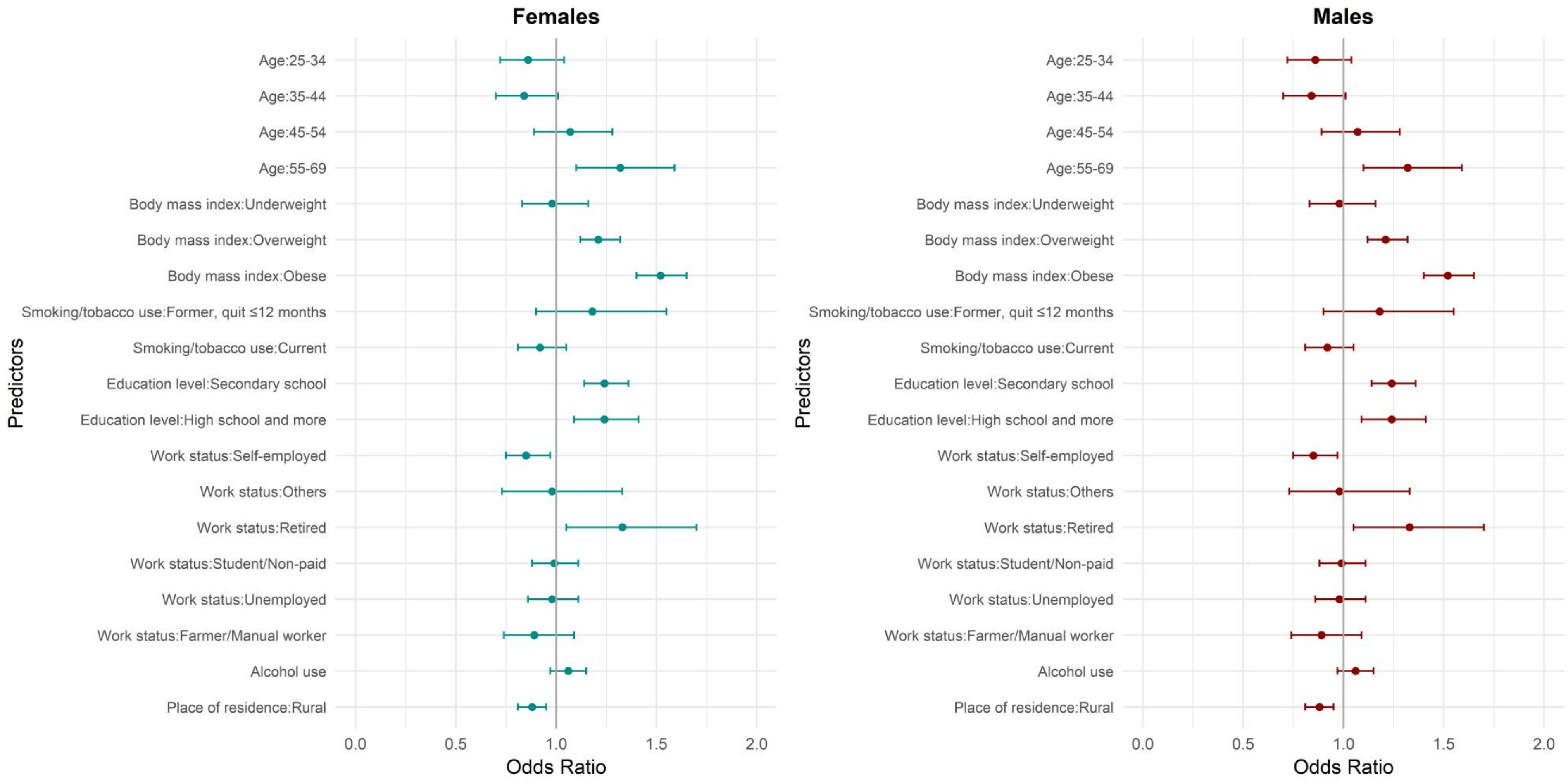

Supplemental Figure 23. Determinants of hypertension awareness: sex-stratified multivariable hierarchical analysis, adjusted for rural/urban status.
Odds ratios (ORs) and their 95% confidence intervals (CIs) were estimated via logistic regression analyses with random effects for country and survey year. The analysis was conducted among hypertensive individuals only. Dots represent adjusted ORs and horizontal lines their 95% CIs for each predictor. An OR greater than 1 indicates a positive association (increased odds of hypertension awareness) and an OR less than 1 indicates a negative association (reduced odds).

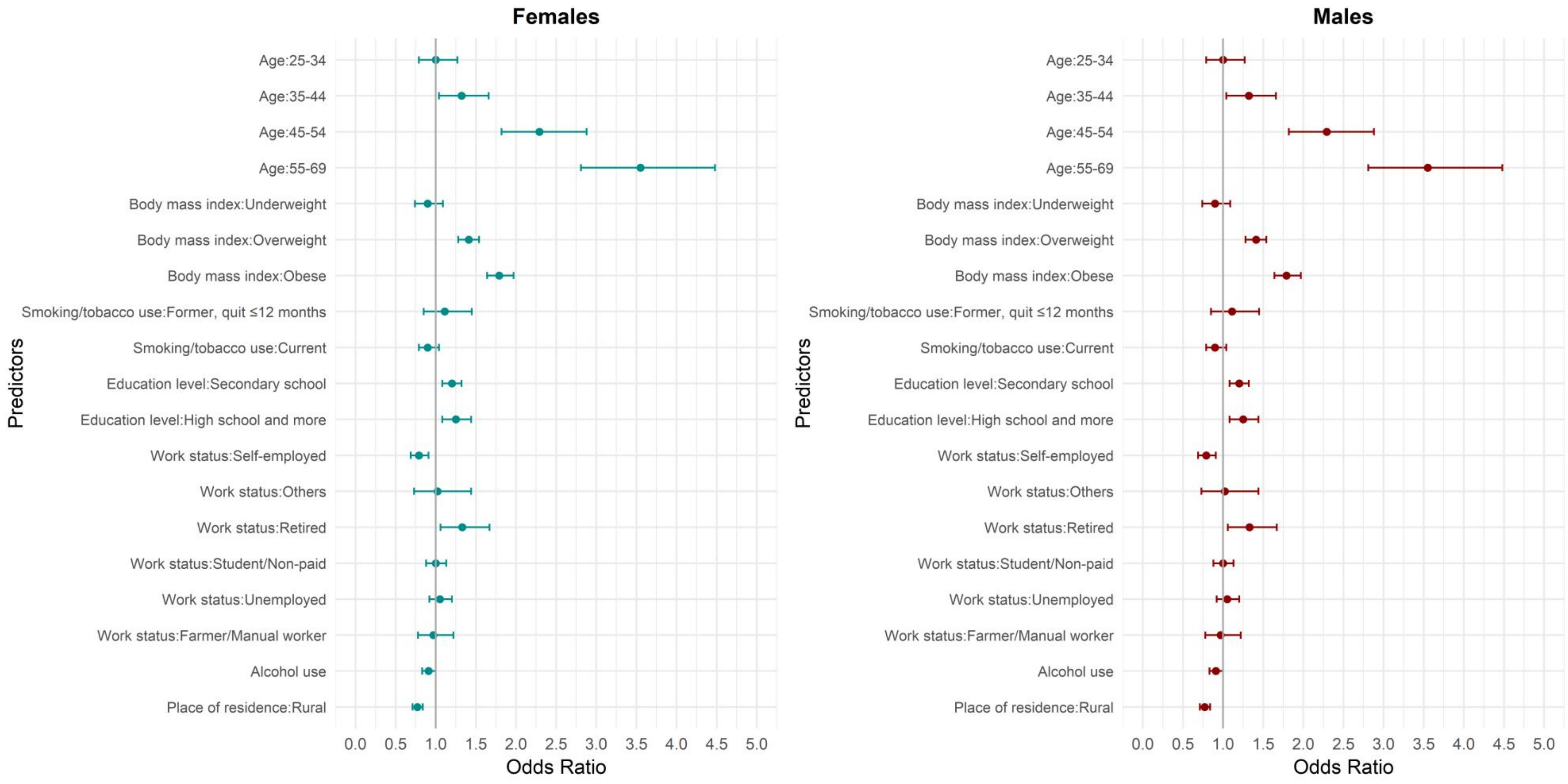

Supplemental Figure 24. Determinants of hypertension treatment: sex-stratified multivariable hierarchical analysis, adjusted for rural/urban status.
Odds ratios (ORs) and their 95% confidence intervals (CIs) were estimated via logistic regression analyses with random effects for country and survey year. The analysis was conducted among hypertensive individuals only. Dots represent adjusted ORs and the horizontal lines their 95% confidence intervals for each predictor. An OR greater than 1 indicates a positive association (increased odds of hypertension treatment) and an OR less than 1 indicates a negative association (reduced odds).

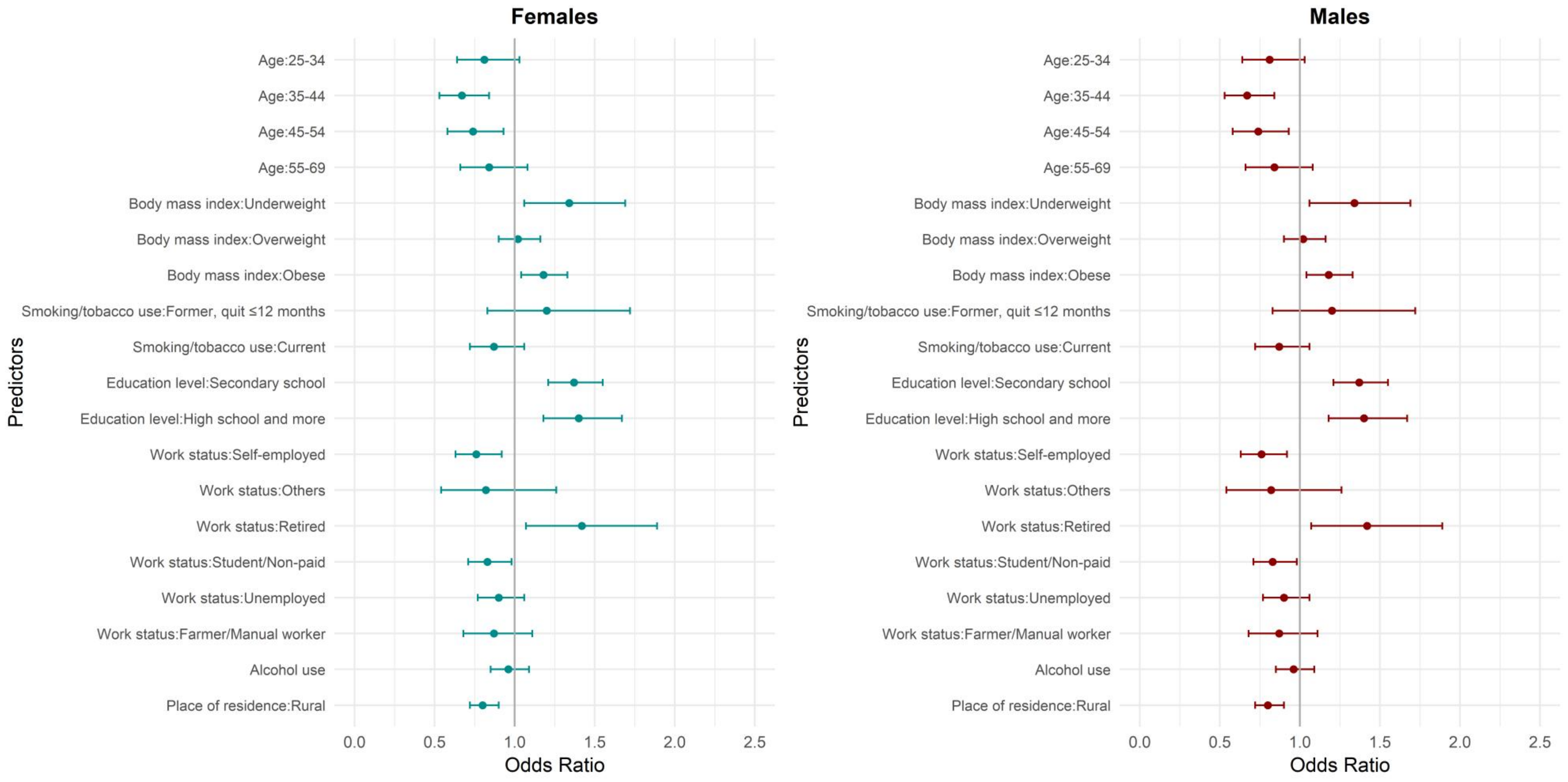

Supplemental Figure 25. Determinants of hypertension control: sex-stratified multivariable hierarchical analysis, adjusted for rural/urban status.
Odds ratios (ORs) and their 95% confidence intervals (CIs) were estimated via logistic regression analyses with random effects for country and survey year. The analysis was conducted among hypertensive individuals only. Dots represent adjusted ORs and horizontal lines their 95% CIs for each predictor. An OR greater than 1 indicates a positive association (increased odds of hypertension control) and an OR less than 1 indicates a negative association (reduced odds).

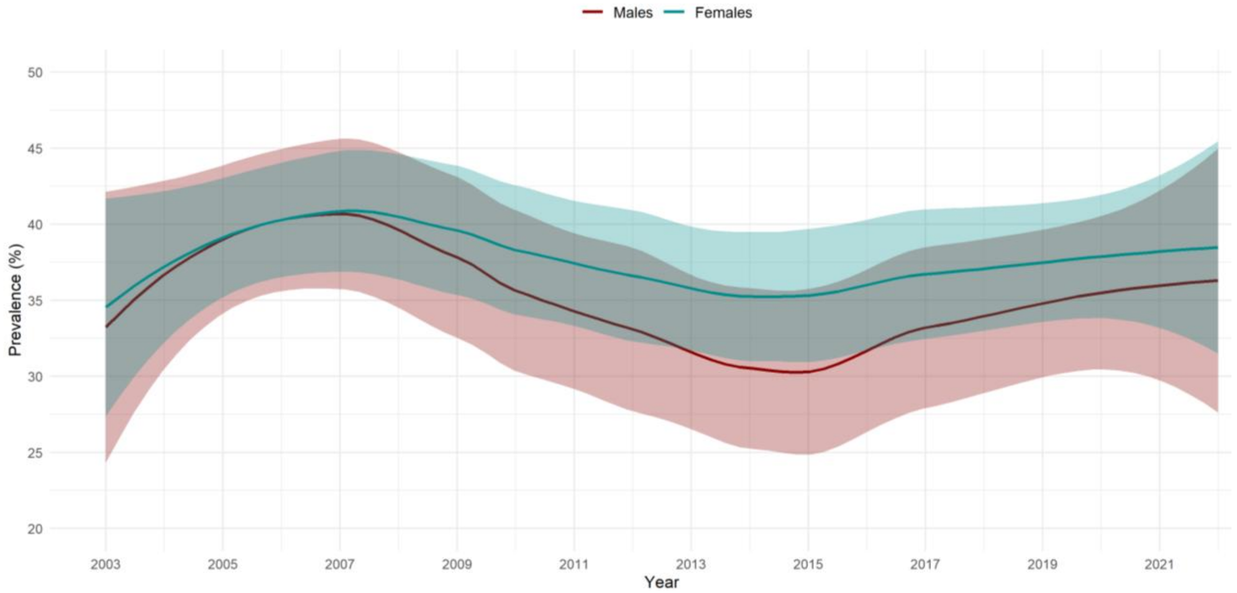

Supplemental Figure 26. Sex-stratified trend in age-standardized prevalence of hypertension from 2003–2022 using observed (non-imputed) data.

Trends in hypertension prevalence in the WHO African region using available data per year and per country: two countries in 2003, three in 2004, four in 2005, two in 2006, six in 2007, five in 2008–2010, four in 2011–2012, three in 2013, five in 2014–2015, two in 2016, four in 2017, one in 2019–2020, three in 2021, and two in 2022. No data were available in 2018. WHO = World Health Organization.

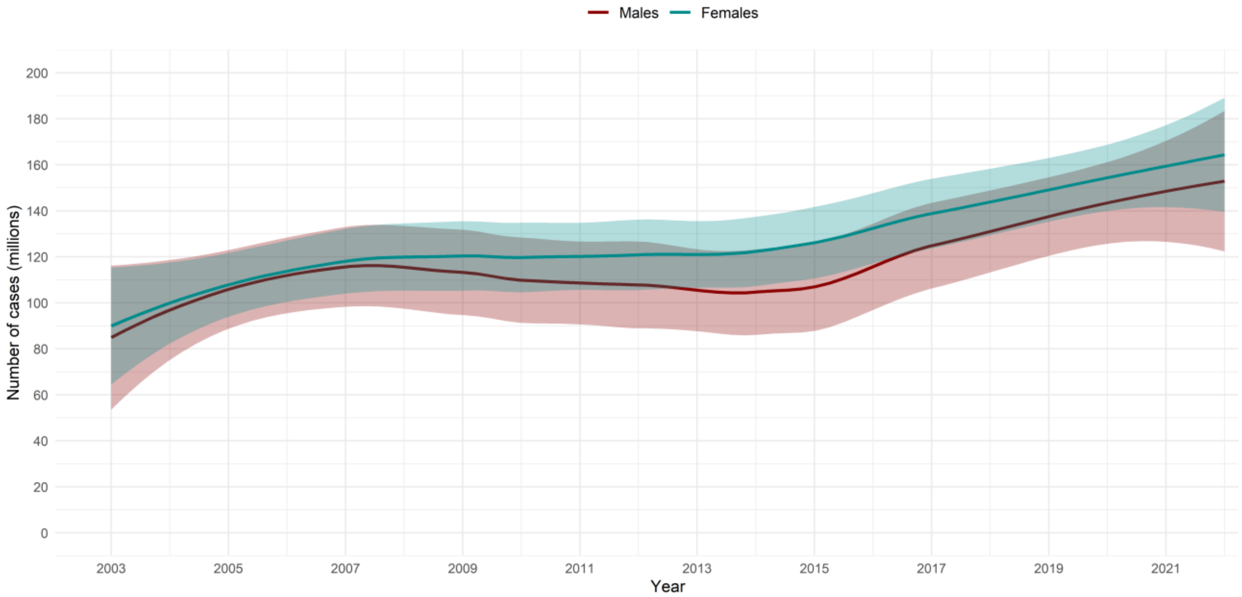

Supplemental Figure 27. Sex-stratified trend in the number of hypertensive individuals from 2003–2022 using observed (non-imputed) data.

Trends in hypertension prevalence in the WHO African region using available data per year and per country: two countries in 2003, three in 2004, four in 2005, two in 2006, six in 2007, five in 2008–2010, four in 2011–2012, three in 2013, five in 2014–2015, two in 2016, four in 2017, one in 2019–2020, three in 2021, and two in 2022. No data were available in 2018. WHO = World Health Organization.

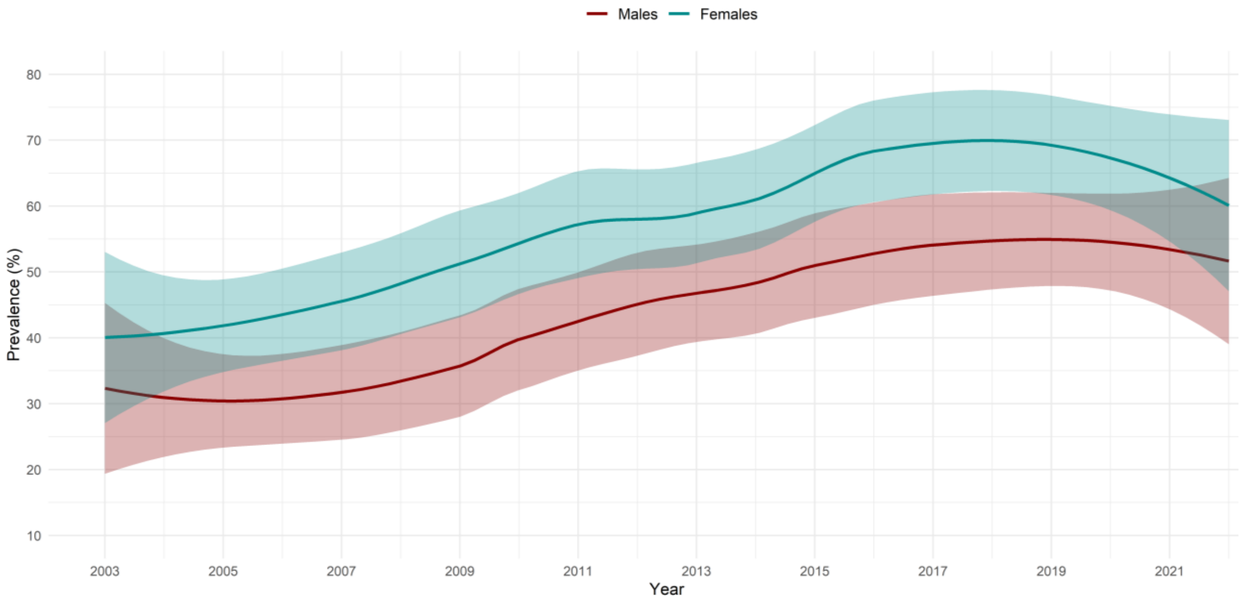

Supplemental Figure 28. Sex-stratified trend in age-standardized prevalence of awareness from 2003–2022 using observed (non-imputed) data.
Trends in hypertension prevalence in the WHO African region using available data per year and per country: two countries in 2003, three in 2004, four in 2005, two in 2006, six in 2007, five in 2008–2010, four in 2011–2012, three in 2013, five in 2014–2015, two in 2016, four in 2017, one in 2019–2020, three in 2021, and two in 2022. No data were available in 2018. WHO = World Health Organization.

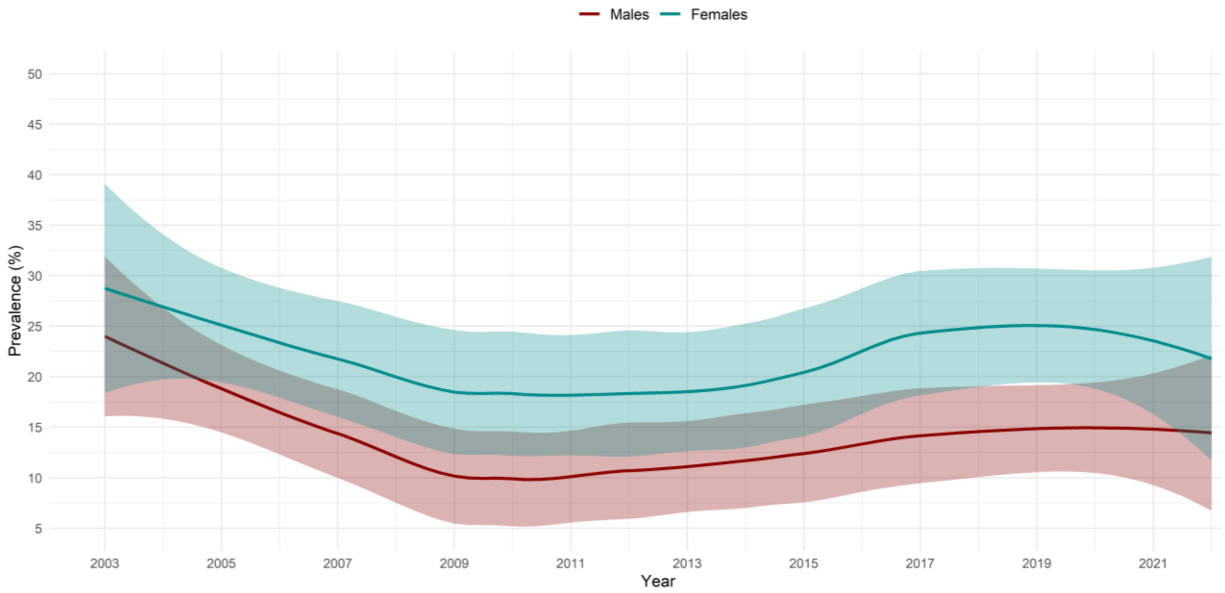

Supplemental Figure 29. Sex-stratified trend in age-standardized prevalence of treatment from 2003–2022 using observed (non-imputed) data.
Trends in hypertension prevalence in the WHO African region using available data per year and per country: two countries in 2003, three in 2004, four in 2005, two in 2006, six in 2007, five in 2008–2010, four in 2011–2012, three in 2013, five in 2014–2015, two in 2016, four in 2017, one in 2019–2020, three in 2021, and two in 2022. No data were available in 2018. WHO = World Health Organization.

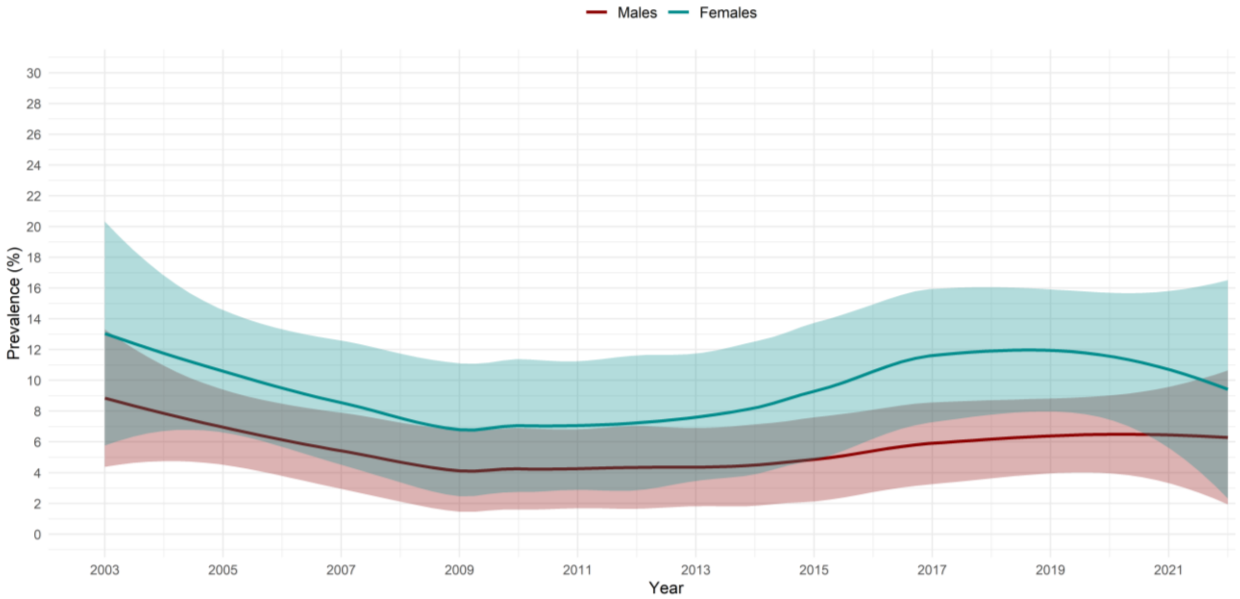

Supplemental Figure 30. Sex-stratified trend in age-standardized prevalence of control from 2003–2022 using observed (non-imputed) data.

Trends in hypertension prevalence in the WHO African region using available data per year and per country: two countries in 2003, three in 2004, four in 2005, two in 2006, six in 2007, five in 2008–2010, four in 2011–2012, three in 2013, five in 2014–2015, two in 2016, four in 2017, one in 2019–2020, three in 2021, and two in 2022. No data were available in 2018. WHO = World Health Organization.

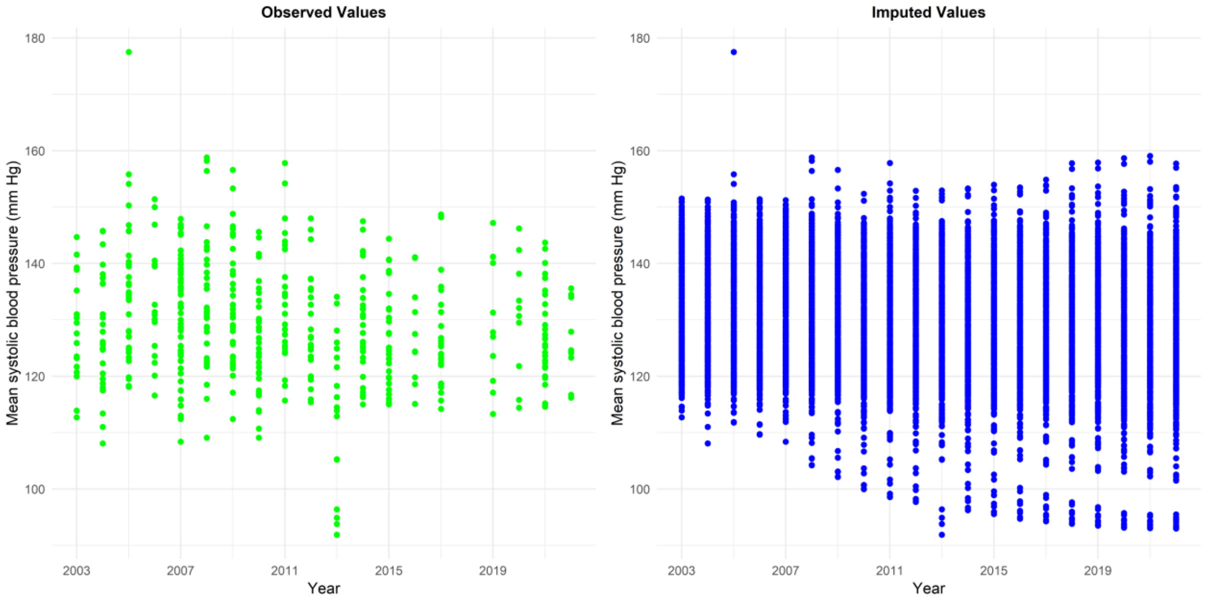

Supplemental Figure 31. Observed and imputed averaged systolic blood pressure values from 2003 to 2022 in the WHO African region by sex and age group (18–24, 25–34, 35–44, 45–54, and 55–69 years) for each available survey for each year. Green dots represent observed values and blue dots represent imputed values. Data were imputed using the spatio-temporal model.[3]

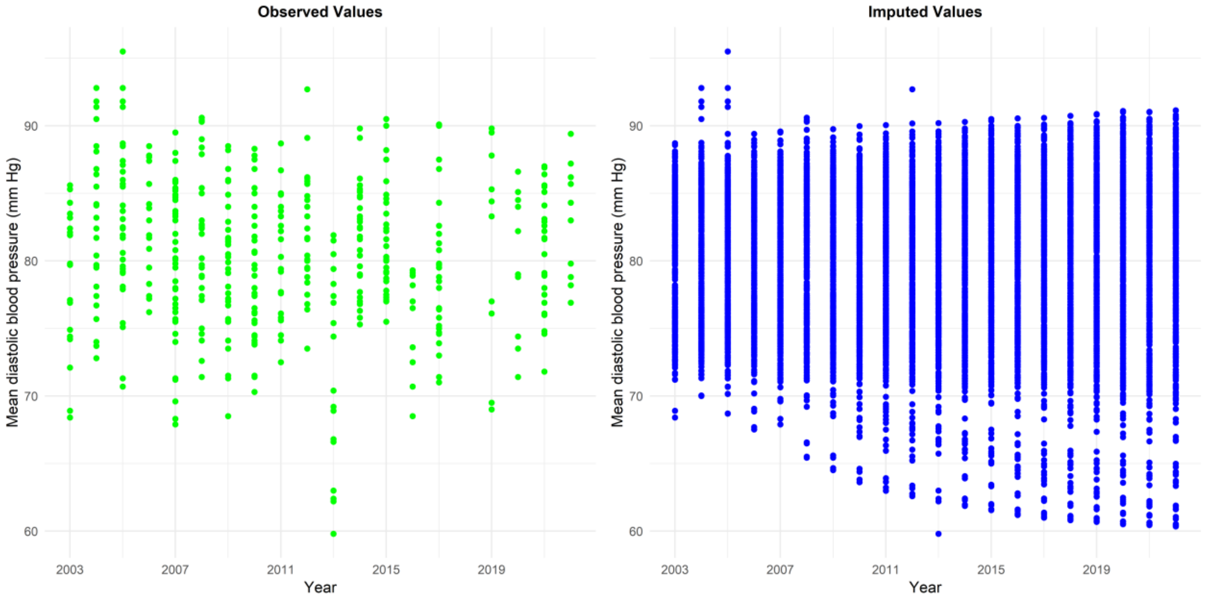

Supplemental Figure 32. Observed and imputed averaged diastolic blood pressure from 2003 to 2022 in the WHO African region by sex and age group (18–24, 25–34, 35–44, 45–54, and 55–69 years) for each available survey for each year. Green dots represent observed values and blue dots represent imputed values. Data were imputed using the spatio-temporal model.[3]

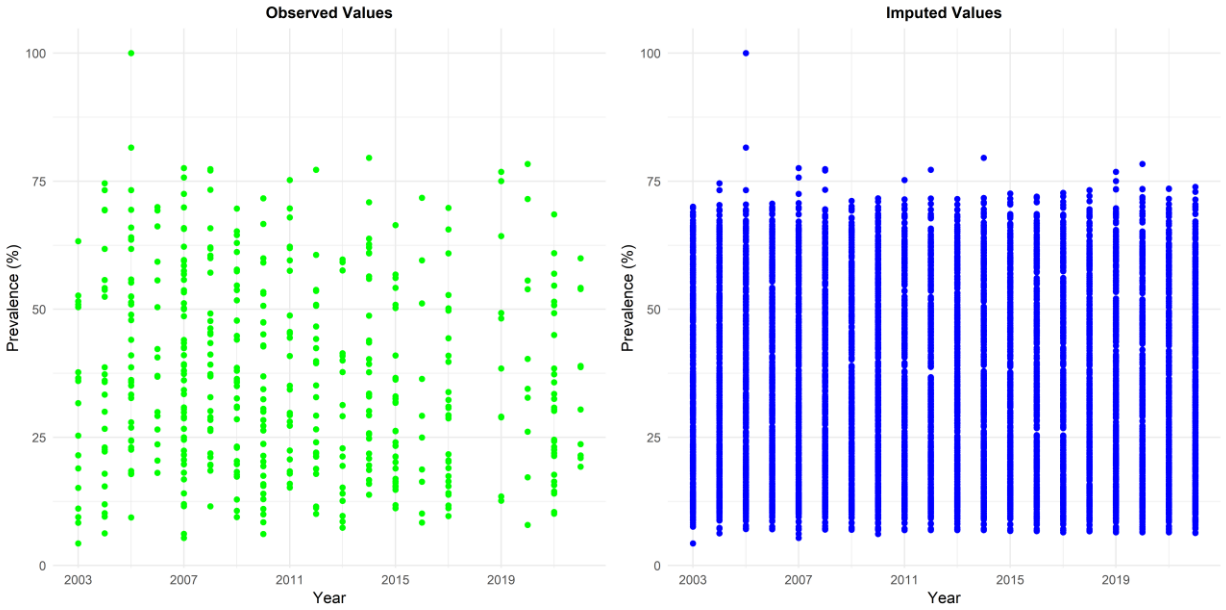

Supplemental Figure 33. Observed and imputed prevalence of hypertension from 2003 to 2022 in the WHO African region by sex and age group (18–24, 25–34, 35–44, 45–54, and 55–69 years) for each available survey for each year. Green dots represent observed values and blue dots represent imputed values. Data were imputed using the spatio-temporal model.[3]

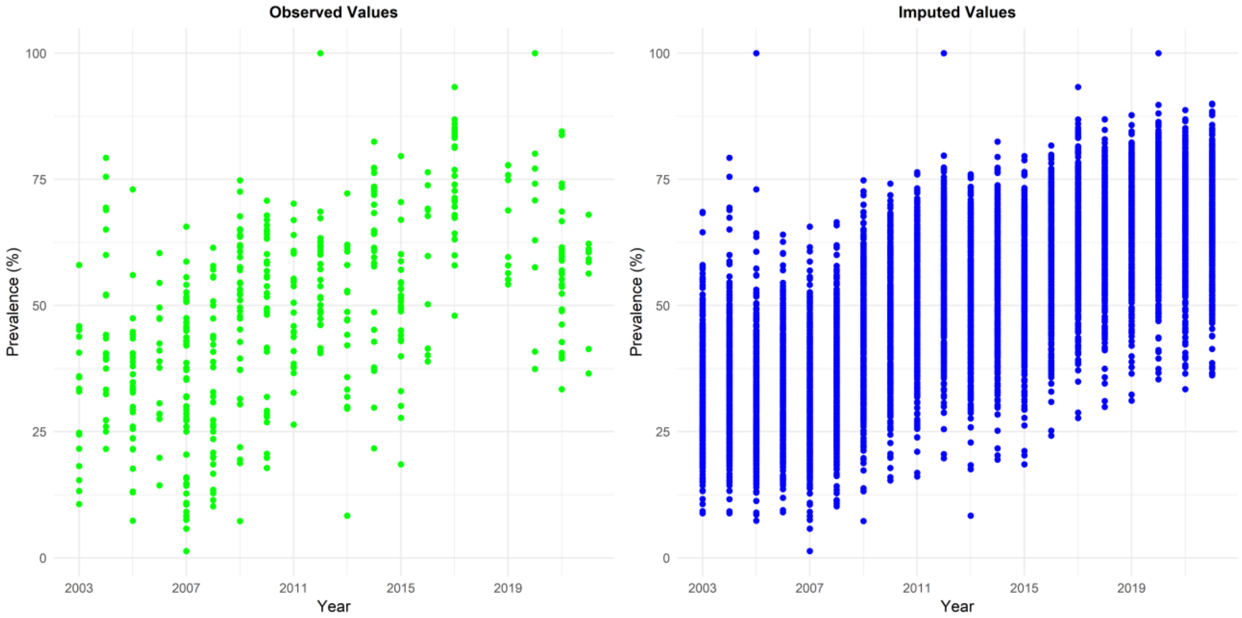

Supplemental Figure 34. Observed and imputed prevalence of hypertension awareness values from 2003 to 2022 in the WHO African region by sex and age group (18–24, 25–34, 35–44, 45–54, and 55–69 years) for each available survey for each year. Green dots represent observed values and blue dots represent imputed values. Data were imputed using the spatio-temporal model.[3]

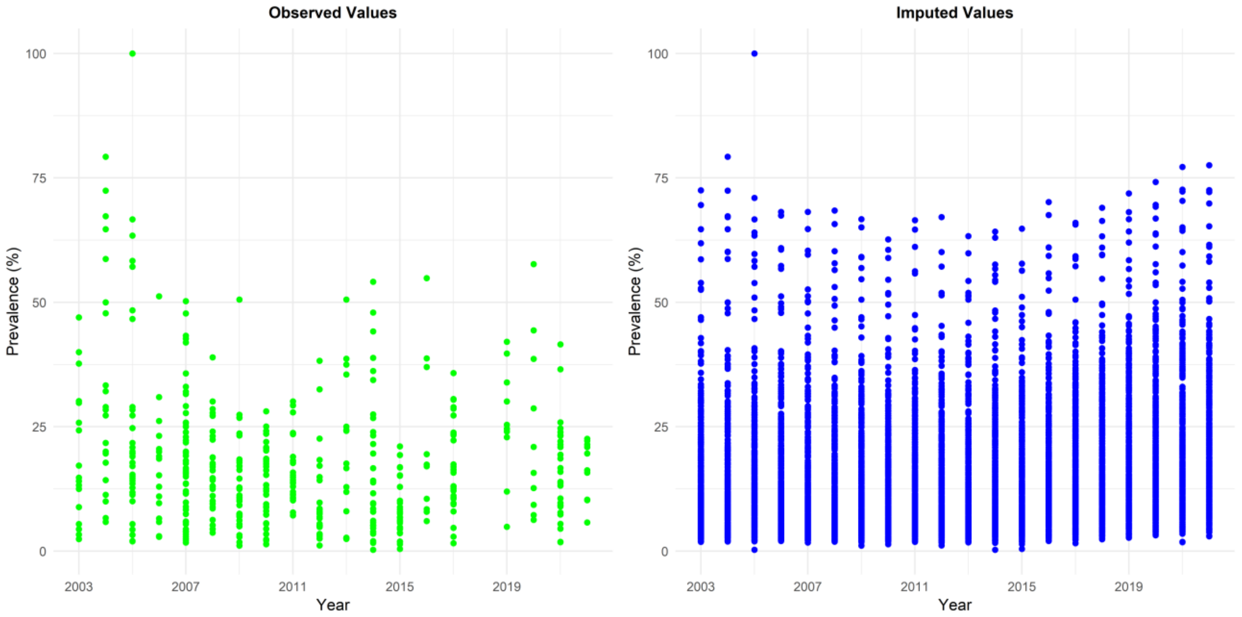

Supplemental Figure 35. Observed and imputed prevalence of hypertension treatment values from 2003 to 2022 in the WHO African region by sex and age group (18–24, 25–34, 35–44, 45–54, and 55–69 years) for each available survey for each year. Green dots represent observed values and blue dots represent imputed values. Data were imputed using the spatio-temporal model.[3]

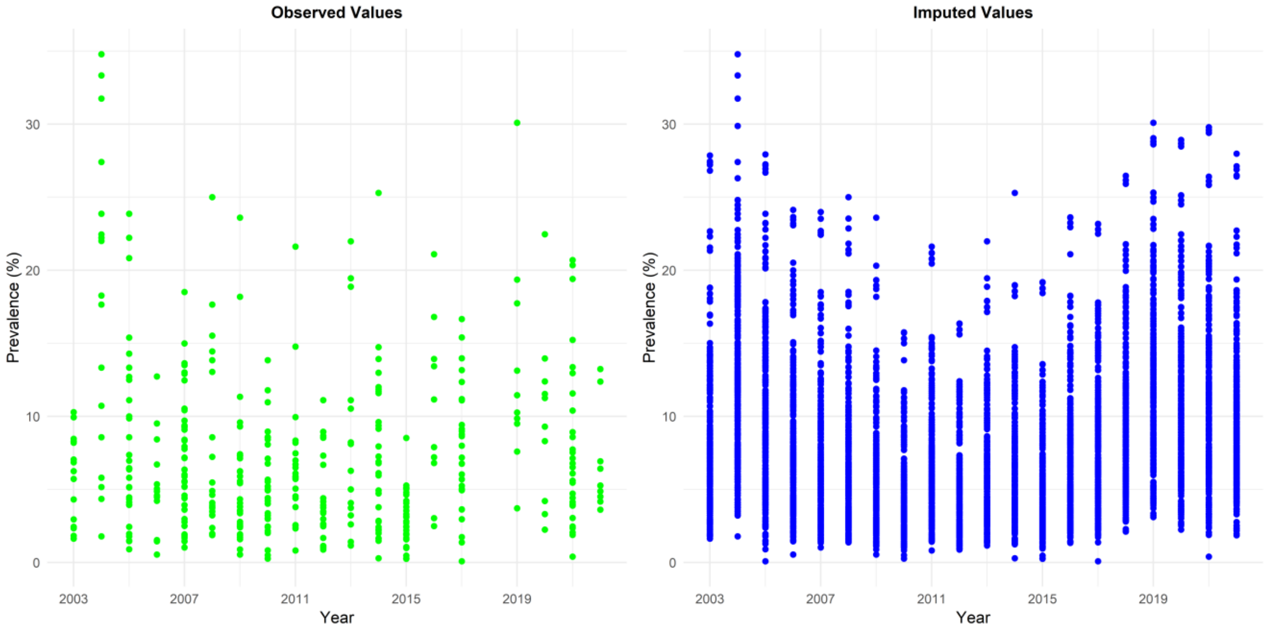

Supplemental Figure 36. Observed and imputed prevalence of hypertension control values from 2003 to 2022 in the WHO African region by sex and age group (18–24, 25–34, 35–44, 45–54, and 55–69 years) for each available survey for each year. Green dots represent observed values and blue dots represent imputed values. Data were imputed using the spatio-temporal model.[3]

References

1. WHO. WHO STEPS Surveillance Manual: The WHO STEPwise approach to noncommunicable disease risk factor surveillance [Internet]. Geneva: World Health Organization; 2017 [cited 2024 Jan 2]. Available from: https://www.who.int/teams/noncommunicable-diseases/surveillance/systems-tools/steps/manuals

2. Croft TN, Marshall AM, Allen CK, Zachary BW, et al. Guide to DHS statistics. Rockville, Maryland, USA: ICF [Internet]. 2023; Available from: https://dhsprogram.com/data/Guide-to-DHS-Statistics/index.cfm

3. Bakka H, Rue H, Fuglstad GA, Riebler A, Bolin D, Illian J, et al. Spatial modeling with R-INLA: A review. WIREs Computational Statistics [Internet]. 2018 [cited 2024 Aug 7];10(6):e1443. Available from: https://onlinelibrary.wiley.com/doi/abs/10.1002/wics.1443

4. Osgood-Zimmerman A, Wakefield J. A Statistical Review of Template Model Builder: A Flexible Tool for Spatial Modelling. International Statistical Review [Internet]. 2023 [cited 2024 Jun 28];91(2):318–42. Available from: https://onlinelibrary-wiley-com.ezproxy.u-paris.fr/doi/abs/10.1111/insr.12534

5. Index GI. Human development reports, United Nations development programme. URL: http://hdr undp org/en/composite/GII. 2015;

6. Mundial GB. World Bank Group: International Development, Poverty & Sustainability. World Bank Group; 2023.

7. Honaker J, King G, Blackwell M. Amelia II: A program for missing data. Journal of statistical software [Internet]. 2011 [cited 2024 Oct 10];45:1–47. Available from: https://www.jstatsoft.org/index.php/jss/article/view/v045i07

8. Bürkner PC. Advanced Bayesian Multilevel Modeling with the R Package brms. The R Journal [Internet]. 2018 [cited 2025 May 17];10(1):395–411. Available from: https://journal.r-project.org/archive/2018/RJ-2018-017/index.html

9. Dutta C, Ravishanker N, Basu S. Modeling multivariate positive-valued time series using R-INLA. Applied Stochastic Models in Business and Industry [Internet]. [cited 2024 Jun 28];n/a(n/a). Available from: https://onlinelibrary-wiley-com.ezproxy.u-paris.fr/doi/abs/10.1002/asmb.2834
